# Chloride Binding Modulated by Anion Receptors Bearing Tetrazine and Urea

**DOI:** 10.1002/cphc.202200524

**Published:** 2022-10-20

**Authors:** Romain Plais, Guy Gouarin, Amélie Bournier, Olfa Zayene, Vanessa Mussard, Flavien Bourdreux, Jérome Marrot, Arnaud Brosseau, Anne Gaucher, Gilles Clavier, Jean‐Yves Salpin, Damien Prim

**Affiliations:** ^1^ Institut Lavoisier de Versailles Université Paris-Saclay UVSQ CNRS 78000 Versailles France; ^2^ LAMBE Université Paris-Saclay Univ Evry CNRS 91025 Evry-Courcouronnes France; ^3^ LAMBE CY Cergy Paris Université CNRS 95000 Cergy France; ^4^ PPSM Université Paris-Saclay ENS Paris-Saclay CNRS 91190 Gif-sur-Yvette France

**Keywords:** anion-π interaction, fluorescence, hydrogen bonding, anion receptor

## Abstract

Modulation and fine‐tuning of the strength of weak interactions to bind anions are described in a series of synthetic receptors. The general design of the receptors includes both a urea motif and a tetrazine motif. The synthetic sequence towards three receptors is detailed. Impacts of H‐bond strength and linker length between urea and tetrazine on chloride complexation are studied. Binding properties of the chloride anion are examined in both the ground and excited states using a panel of analytical methods (NMR spectroscopy, mass spectrometry, UV/Visible spectroscopies, and fluorescence). A ranking of the receptors by complexation strength has been determined, allowing a better understanding of the structure‐properties relationship on these compounds.

## Graphical abstract

**Structural modifications** of a simple anion receptor bearing urea and tetrazine fragments were studied in both ground and excited states. Receptor **2** shows slightly improved chloride complexation compared to the published receptor **4**.

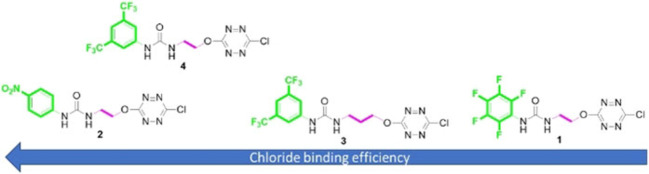

## Introduction

**Figure 1:**
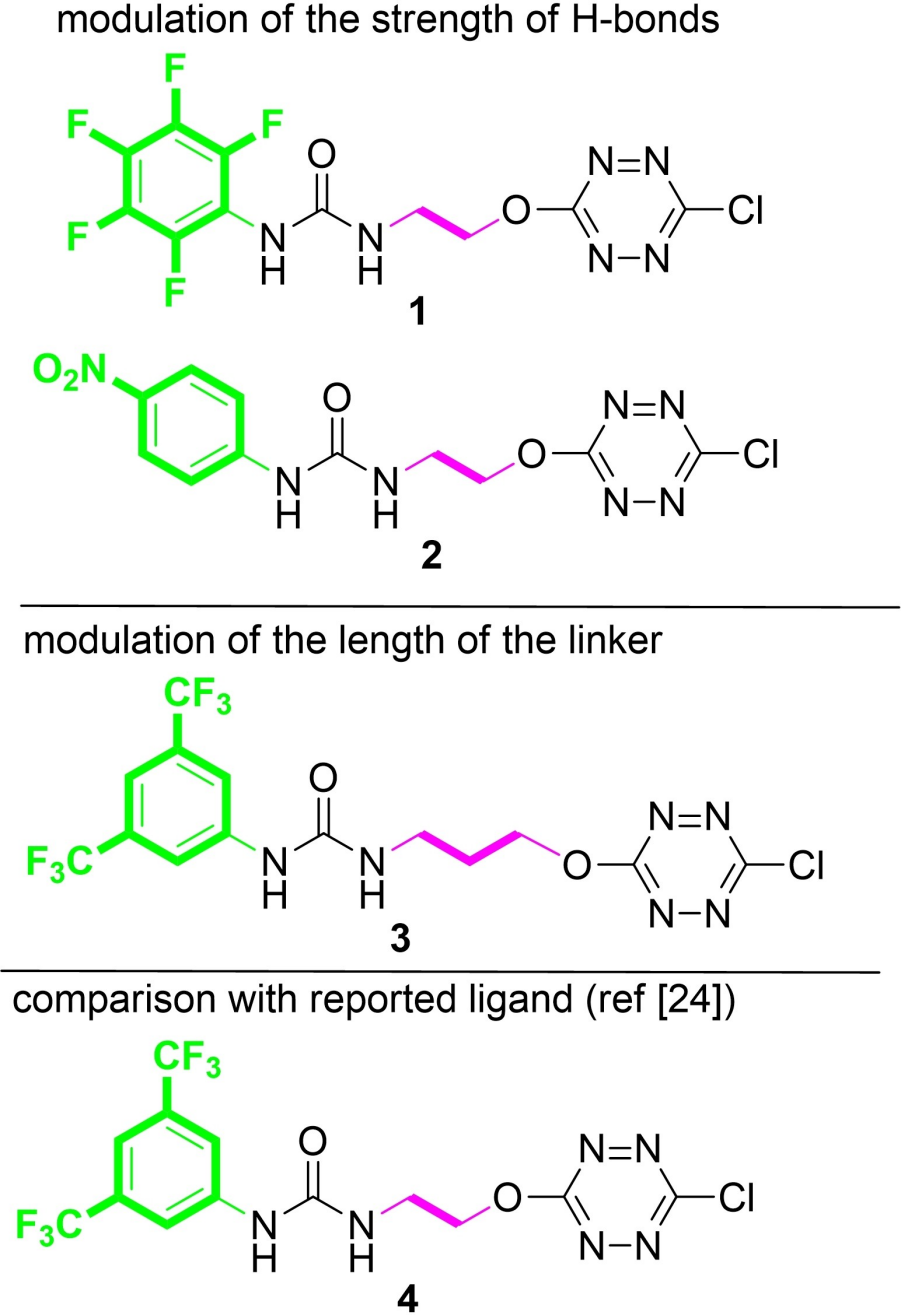
Ternary receptors **1**–**3**.

Noncovalent bond interactions play a significant role in the supramolecular processes involved in different scientific domains such as biological machineries, chemical, environmental and material sciences.[^1^, ^2^, ^3^, ^4^, ^5^, ^6^, ^7^, ^8^] Because of their involvement in vital biological systems, anion recognition is becoming of increasing interest.[^9^, ^10^, ^11^, ^12^, ^13^, ^14^] Understanding and quantifying weak interactions remains challenging and is crucial for the development of modular and selective synthetic receptors.[^15^, ^16^] These latter consist in the assembly of molecular building blocks capable of generating non‐covalent interactions. The organization of the various fragments has a significant impact on the overall shape and binding properties of the architecture. Thus, the design of new selective anion receptors is directly linked to the nature, number, and location of weak bond donor patterns.[^17^, ^18^, ^19^, ^20^, ^21^] Until recently, anion receptors contained mostly identical or similar types of weak interactions. The combination of several distinct weak interactions and their potential cooperativity in binding events is an emerging approach.[^22^, ^23^] Within the same receptor, the joint presence of both a urea and a tetrazine fragment proved meaningful, allowing (i) taking advantage of the tetrazine moiety acting as π‐anion acceptor and on/off fluorescence probe, (ii) combining several complementary experimental techniques to characterize anion complexes, and (iii) gaining insights into the complexation event.[^24^, ^25^] In this context, refining the strength, directionality and complementarity of key fragments is of major interest. Here we present the study of the chloride anion binding properties of a family of ternary receptors. We focused our attention on three prospect lines. We first examined the modulation and fine‐tuning of the strength of Hydrogen bonds arising from the urea motif. We next evaluated the remoteness of both the urea and the tetrazine fragments, and thus the flexibility of the link between both entities. In this context, the development of three new receptors **1**–**3** has been considered to evaluate the effect of both H‐bond strength and linker flexibility on the complexation of chloride anion. The current results were also compared with the already reported synthetic receptor **4** *(*Figure 1 *)*.^[24]^

## Results and Discussion

### Synthesis of Receptors

The synthetic approach to the unprecedented receptors **1**–**3** is based on a one pot‐two steps short sequence *(*Scheme 1 *)*. Receptors **1** and **2** were obtained from the condensation of ethanolamine to pentafluoro phenyl‐ and nitrophenyl‐isocyanate, respectively, leading to the corresponding urea intermediates using a recently reported procedure.[^24^, ^26^] The latter were next reacted with dichlorotetrazine, leading to receptors **1** and **2** in 23 % and 33 % yields, respectively.[^24^, ^27^] Moving from ethanol‐ to propanolamine in the same sequence led to the preparation of receptor **3** (53 % yield), in which both the urea and the tetrazine units are separated by one additional carbon atom (*ESI, See §3, Figures S1 to S38*).

**Scheme 1:**
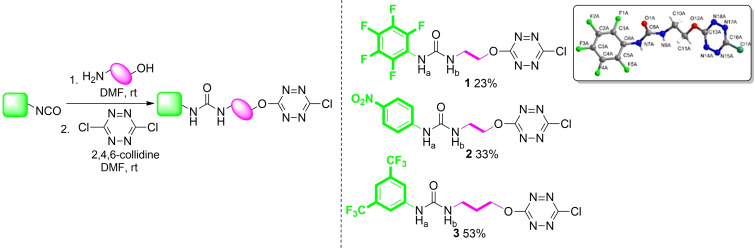
Synthesis of ternary receptors **1**–**3** and X‐Ray structure of **1**.

Single crystals suitable for X‐ray analysis were obtained by slow evaporation of acetone solution of receptor **1** *(Insert*, Scheme 1 *)*. It is worth noting that (i) receptor **1** adopts an almost linear conformation (ii) no π–π interaction could be characterized *(ESI, Figure S24)*. The tridimensional arrangement is set by the presence of intermolecular H‐bonds between urea fragments of two receptor molecules.

### Ground‐State‐Binding Studies by NMR, Mass and UV/Vis Spectroscopy

**Figure 2:**
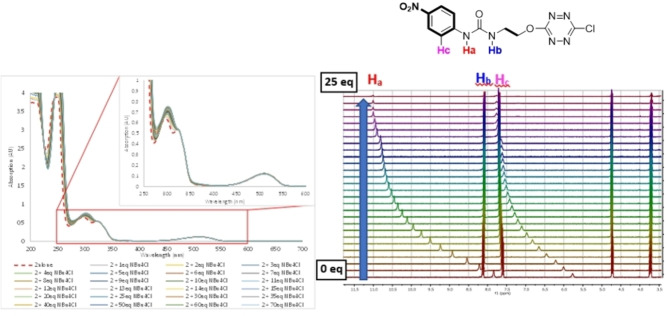
UV‐Visible and NMR titrations of **2** by NBu_4_Cl in acetonitrile.

The affinity of **1**–**3** towards Cl^−^ was assessed first by ^1^H NMR titrations in CD_3_CN *(ESI, Figures S42 to S48)*. In all receptors, urea protons **H_a_** and **H_b_** are privileged spectators of the complexation phenomena. Indeed, the downfield shifts of both urea protons between the receptor and the corresponding complex are indicative of an interaction with the anion. As a typical example, Figure 2 shows the downfield shifts of urea protons for receptor **2**. Furthermore, the comparison of **Δδ**H_a_ (**δ**H_a,complex_‐**δ**H_a,receptor_) represents a qualitative scaling tool to compare the strength of the complexation to chloride anion on similar receptors. In ternary complexes **1**–**3** and **4**, **Δδ**H_a_ ranges from 2.74 to 3.05, 3.16 and 3.23 for **1**‐Cl, **3**‐Cl, **2**‐Cl and **4**‐Cl respectively, allowing anticipating that (i) **2**‐Cl should exhibit an increased binding strength by comparison with **1**‐Cl and **3**‐Cl; (ii) the binding strength of **2** and **4** should be similar. Binding constants have been determined as recently described and gathered in Table 1.[^24^, ^28^, ^29^]

**Table 1:** Analysis of NMR titration data.

Entry	Complex	**Δδ**H_a_^[**c**]^	**Δδ**H_b_^[**c**]^	K_A, SPECFIT_^[a]^
1	**1**‐Cl	2.74	2.11	758±1 (0,01 %)
2	**2**‐Cl	3.16	1.99	4971±1 (0,5 %)
3	**3**‐Cl	3.05	1.86	982±1 (0.5 %)
4	**4**‐Cl	3.23	1.75	4892±1 (0.6 %)^[b]^

[a] Binding constants in L/mol. [b] ref [^[24]^] [c] Δδ between chemical shifts of the receptor and the corresponding Cl‐complex after addition of 25 eq. of chloride anion.

Examination of NMR data deserves some comments. As shown in table 1, receptors **2** and **4**, bearing either a nitro substituent or two CF_3_ groups respectively, turned out to be more efficient than ternary receptors **1** and **3**,in good agreement with literature.[^30^, ^31^] Strong electron withdrawing groups such as nitro or trifluoromethyl induce highest variation of chemical shifts of proton H_a_. A highest variation correlates with highest impact on association constants. The high electron withdrawing capacity of nitro or trifluoromethyl groups most likely impacts the N‐H_a_ bond by increasing the acidity of proton H_a_ and providing enhanced hydrogen bonding properties. H_b_ being further away from the aromatic moiety than H_a_, the impact of the substituents of the aromatic ring on H_b_ is reasonably less significant. Therefore, H_b_ becomes somewhat less informative than H_a_. However, it may allow sharply discriminate between receptors **2** and **4**. The fact that the receptor **2** exhibits a slightly higher binding constant by comparison with **4**, can be rationalized by the contributive impact of H_b_ to the overall binding process. Indeed, **Δδ**H_b_ for **2** (1.99) is higher than for **4** (1.75). Moving from ethylene to propylene linker in **3** turned to be less efficient. Increased linker flexibility (**3** *vs* **4**) induces a detrimental effect on the experimental binding constant (Table 1, compare entries 3 and 4).

Analyses of absorption spectra for all receptors and complexes have been performed (*ESI, Figures S49 to S63*). The attribution of the different electronic transitions to the corresponding absorption bands, was achieved by comparison with the theoretical UV‐Visible spectrum (PBE0/6‐311+G(d,p)//APFD/6‐31+G(d,p)).

In ternary receptors **1**, **2**, **3**, titration with tetrabutylammonium chloride in acetonitrile revealed that (i) the tetrazine n‐>π* and π‐>π* absorption bands are either weakly or not affected; (ii) π‐>π* absorption bands of aryl urea fragments undergo clear hyperchromic and bathochromic shifts (*ESI, Figures S64* [**1**], *S72* [**2**] and *S80* [**3**]). These observations show that hydrogen bonding with urea fragments represents the major interaction in both solution and the ground state, in good agreement with the behavior of **4**.^[24]^

### Mass Spectrometry Study

**Figure 3:**
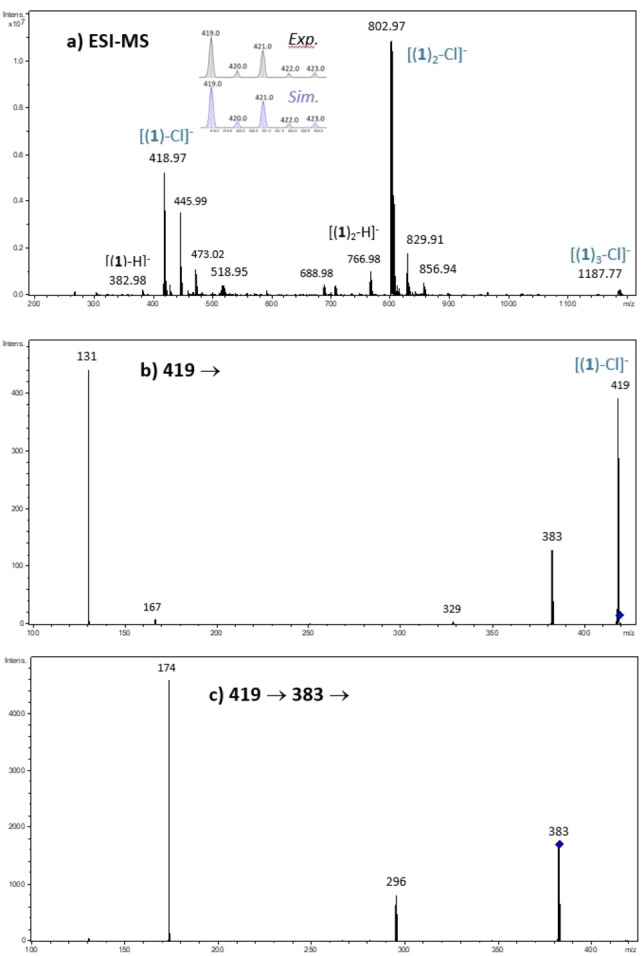
**a)** Electrospray mass spectrum of an equimolar (10^−4^ M) mixture of **1**/NBu_4_Cl (in insert comparison between the experimental and theoretical isotopic distributions of the 1 : 1 adduct) **b)** MS/MS spectrum of the [(**1**)+Cl]^−^ ion (m/z 419) – **c)** MS^3^ spectrum of the [(**1**)‐H]^−^ ion (m/z 383).

In order to determine the nature of the complexes that may arise in the gas phase from the intrinsic interaction of chloride with the different receptors, electrospray mass spectra (ESI) were recorded on a 3D ion trap mass spectrometer (Bruker amaZon Speed ETD). A typical ESI spectrum is given in Figure 3a for the receptor **1**. Those recorded for receptors **2** and **3** are given in the supporting information (ESI, *Figures S39 and S40*). Electrospray spectra are remarkably simple and are dominated by the Cl^−^/receptor complexes [(receptor)_n_+X]^−^ (n=1,2). The stoichiometry can be ascertained by examining the isotopic distribution, which clearly indicates the number of Cl atoms within the complexes. As compared to receptors having two arms on the tetrazine moiety,^[25]^ the intensity of the [(**receptor**)_2_+X]^−^ is sensibly higher, the rather small size of the receptors favoring the 1 : 2 stoichiometry. The 1 : 3 complexes are also observed but in very weak abundance. The deprotonated receptors are also systematically observed (presently *m/z* 383 and the dimeric form at *m/z* 767, Figure 3). Other remarkable ions are detected at *m/z* 446 and 830 on one hand, and *m/z* 473 and 857 on the other hand, which can be attributed to [(**receptor**)_m_,‐H.HCOONH_4_]^−^ (m=1,2), and [(**receptor**)_p_,‐H.NaCN.CH_3_CN]^−^ (p=1,2), respectively, based upon isotopic distributions showing that these ions incorporate one chlorine atom less, as compared to concomitant complex.

**Scheme 2:**
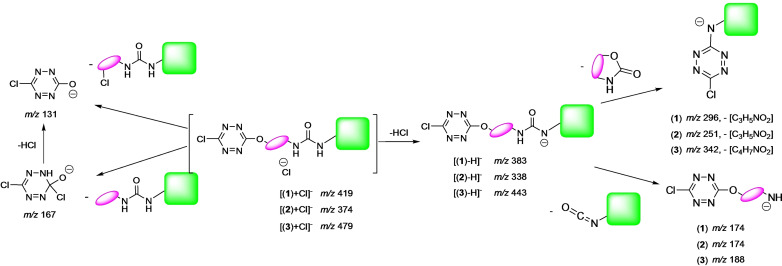
Common dissociation schemes observed with molecules **1**–**3** in interaction with chloride anion.

MS/MS spectra of the 1 : 1 and 1 : 2 complexes were recorded. The unimolecular dissociation upon collision induced dissociation (CID) of the [(**receptor**)_2_+Cl]^−^ ions is solely characterized by the elimination of the intact receptor (*Figures S39 and S40*). The fragmentation spectra of the 1 : 1 complexes are much more informative, and can be summarized by the following dissociation scheme (Scheme 2).

Starting from the [(**receptor**)_1_+Cl]^−^, fragmentations involving the cleavage of covalent bonds are systematically observed, therefore suggesting a strong interaction of Cl^−^ with the receptor. Elimination of HCl associated to deprotonation of the receptor is observed for all the receptors **1**–**3**. This process is associated with the removal of one of the urea proton, very likely Ha, as the resulting deprotonated receptor would be stabilized by the benzenic ring through mesomeric effect.^[24]^ This is also consistent with the downfield shift of the Ha chemical shift observed by NMR. This fragmentation therefore indicates an interaction with the urea motif. The two other fragment ions observed, namely *m/z* 131 and 167, both involve the tetrazine group, and are most likely generated by nucleophilic addition of chloride anion to one of the tetrazine carbon atoms, and therefore suggest the interaction of Cl^−^ with the tetrazine unit. Similar dissociation channels have been already reported for the receptor **4**.^[24]^

We also performed a series of MS^3^ experiments in order to record the MS/MS spectrum of the deprotonated receptor. Two main processes were systematically observed. The first one involves the cleavage of a C−N bond within the urea moiety, leading to a fragment ion including the tetrazine moiety and the alkyl chain (*m/z* 174, Figure 3c) and concomitant elimination of a NCO‐benzenic ring neutral fragment, hence consistent with the deprotonation of the urea motif. The second process is characterized by the loss of the alkyl chain and part of the urea group, possibly through the elimination of a cyclic lactam neutral fragment, by an internal nucleophilic attack (presently *m/z* 296, Figure 3c). Some dissociation mechanisms are proposed in the supporting information, to account for the observed fragment ions (*ESI, §4*). In order to ascertain the structure of both the complexes and fragment ions, experiments have also been performed on a high resolution Q‐TOF instrument (Waters Xevo‐G2S), allowing accurate mass measurements. Results are gathered in Table S1 of the supporting information. An excellent agreement is observed between the experimental *m/z* values measured and the monoisotopic values of both the complexes and the fragment ions postulated, thereby confirming their chemical formula. CID spectra do not show the presence of Cl^−^, indicating that the elimination on the intact receptor is precluded, whereas it has been observed for receptor **4** with PF_6_^−^, Br^−^ and I^−^.^[24]^ This information could not be deduced from ion trap experiments due to low mass limitations of the instrument.

We also tried to record with the QTOF instrument the breakdown diagrams associated with the fragmentation of the three [(**receptor**)+Cl]^−^ ions. These curves are given in the supporting information (*Figure S41*). First, we could observe that fragmentation already occurred with the lowest collision energy applied, showing that 1 : 1 complexes were readily prone to dissociate. Furthermore, the *m/z* 131 ion ([C_2_ClN_4_O]^−^) is clearly overwhelming, indicating that its intensity during ion trap experiments was in fact underestimated due to the low mass cutoff limitation of the ion trap. If one excepts *m/z* 131, the deprotonated receptor is the only additional fragment ion showing a noticeable intensity evolution. Its intensity slightly increases and then decreases. This was not unexpected because the deprotonated receptor further dissociates to generate other fragment ions (Figure 3). Globally, the three curves are similar and show a very fast dissociation process for the three complexes. The complex with receptor **2** does not exhibit a peculiar stability with respect to the two other compounds.

To summarize this gas‐phase study, mass spectrometry experiments suggest strong interactions of Cl^−^ with both the urea and the tetrazine units in the gas phase, but do not allow highlighting a peculiar strong affinity of Cl^−^ towards receptor **2**.

### Excited‐State Binding Studies by Means of Steady State and Time‐Resolved Fluorescence Emission

Excited state interactions in solution were next evaluated. Binding of chloride anion by ternary receptors was further assessed by steady state fluorescence spectroscopy and time‐resolved fluorescence (*ESI, Figures S64 to S87)*. Fluorescence quantum yields ranges from 30 % for **2** to 49 % for **1**, which are globally similar to receptor **4** (*ESI, Figures S49 to S51*). As a typical example, the fluorescence evolution of receptor **3** by iterative addition of chloride is shown in Figure 4. Despite the addition of a large excess of chloride, only a partial quenching of the emission is obtained. A residual fluorescence phenomenon is observed for all ternary receptors (**1**–**3**) in good agreement with reported studies.^[24]^

**Figure 4:**
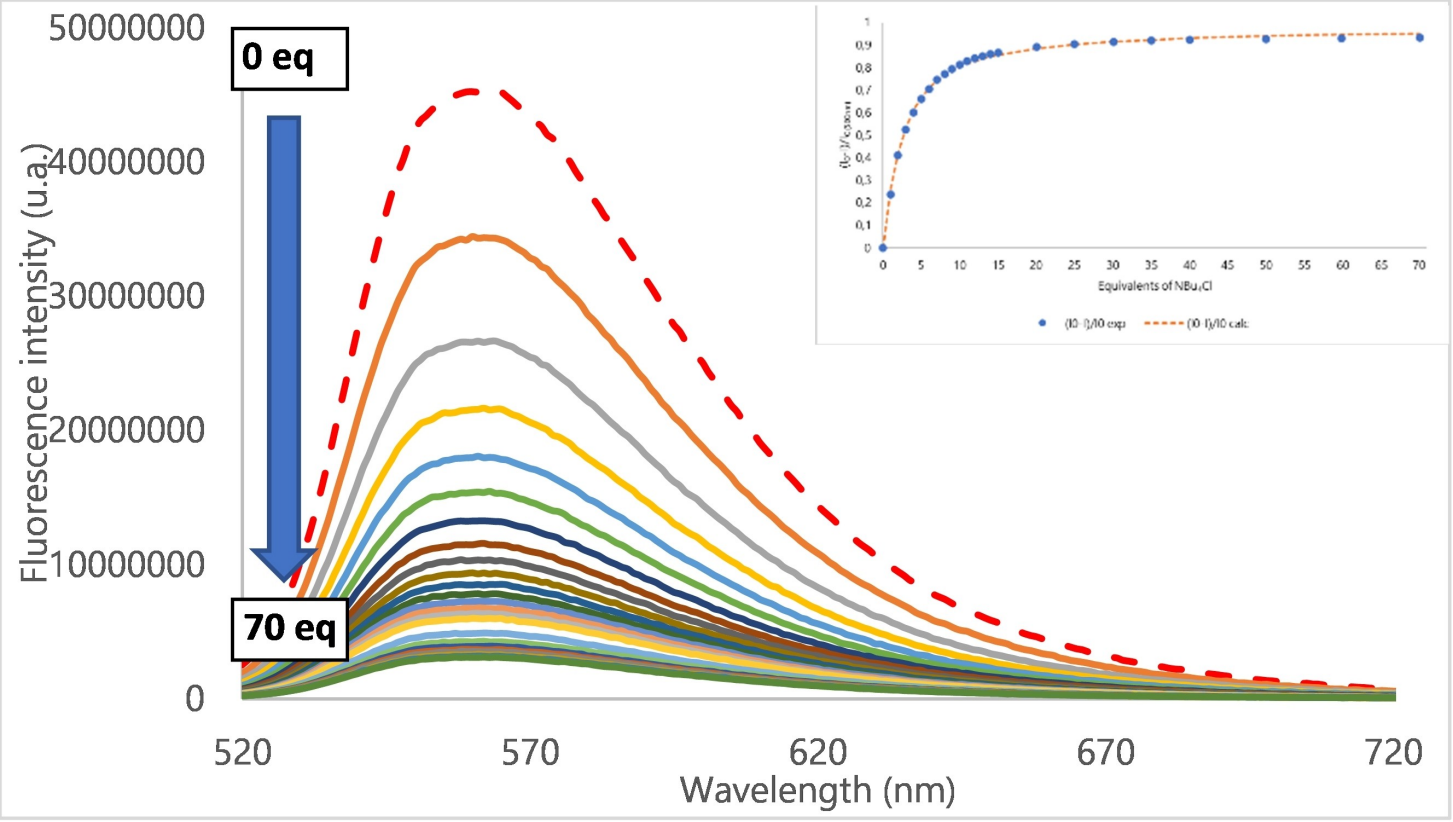
Fluorescence titration of **3** by NBu_4_Cl (insert: determination of association constant).

**Figure 5:**
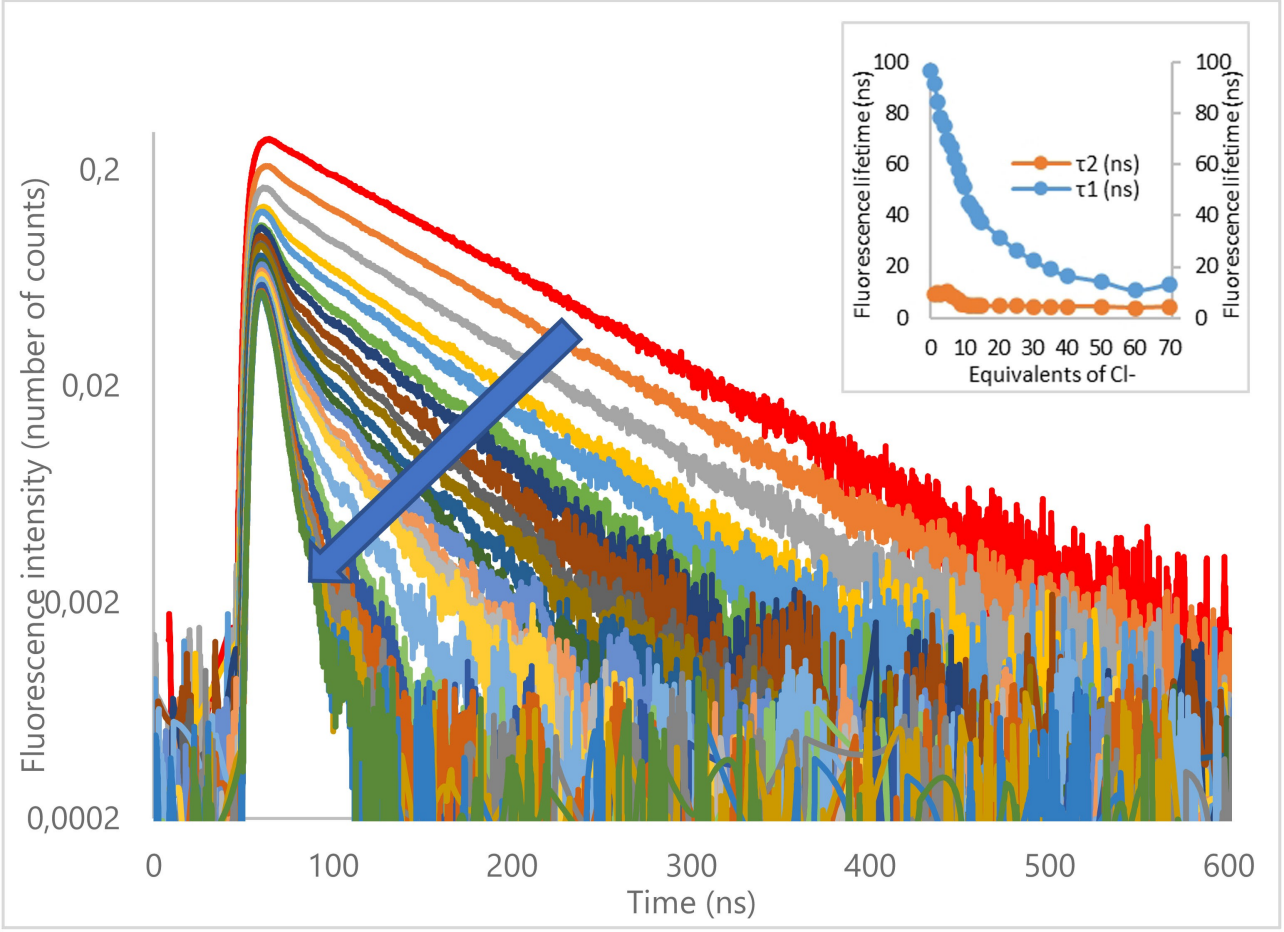
Fluorescence decay titration of **3** by NBu_4_Cl (insert: evolution of fluorescence lifetimes).

As it can be seen from fluorescence decays recorded upon gradual addition of chloride anion, at first the initial fluorescence intensity is affected (for **3**‐Cl, see Figure 5, for other complexes, see *ESI, §6.4 and 6.5*). Upon further addition of chloride anions, the slope of the decay rapidly decreases. Similar trends can be observed for all ternary receptors. In each case, the lifetime of the receptor decreases and is accompanied by the appearance of the corresponding weakly fluorescent complex (see *Insert* Figure 5 for **3**‐Cl), which exhibits a second steady short lifetime.

The maximum fluorescence intensity was also analyzed using a modified Stern‐Volmer equation to take into account the residual fluorescence of the complex (see *ESI §6.7 for details*).[^32^, ^33^] It is clear from the graph in Figure 5, that the fluorescence intensity ratio deviates upward from linearity, which is the sign of the coexistence of two quenching mechanisms (*ESI, Figure S88*). These observations are in good agreement with a quenching mechanism involving both static and dynamic quenchings.

Our data suggest that the interaction of chloride with the tetrazine moiety of the receptor predominantly occurs in the excited state. Both urea motif and the tetrazine unit are thus involved in the complexation process.

Association constants were determined from absorption and fluorescence experiments (Table 2 *)*. In absorption experiments, a higher association constant was obtained with **2**‐Cl than other complexes. High relative errors of fits (>9 %) prevent us from having a significant classification of anion receptors. From fluorescence experiments, a higher association constant was also obtained with **2**‐Cl (3475 L/mol), confirming results obtained from NMR experiments. Then, a slightly higher association constant is observed for **3**‐Cl (2575 L/mol) than **4**‐Cl (1875 L/mol), followed by **1**‐Cl (759 L/mol). In the excited state, chloride ions show a surprisingly higher affinity for receptor **3** than for receptor **4**. The increase in the overall flexibility of the receptor is likely to impact the molecular shape of the complex but also the double quenching phenomenon and thus partly explain this evolution of Ka

**Table 2:** Analysis of photophysical data.

Entry		K_A, abs, SPECFIT_^[a]^	K_A, fluo SPECFIT_^[a]^
1	**1**‐Cl	983±1 (2 %^[b]^)	759±1 (2 %^[b]^)
2	**2**‐Cl	2108±1 (9 %^[b]^)	3475±1 (2 %^[b]^)
3	**3**‐Cl	22±2 (13 %^[b]^)	2575±1 (3 %^[b]^)
4	**4**‐Cl	108±1 (10 %^[b]^)	1875±1 (1 %^[b]^)

^[a]^All association constants are expressed in L/mol ^[b]^ Relative error of fit.

### Molecular Modelling Approach

**Figure 6:**
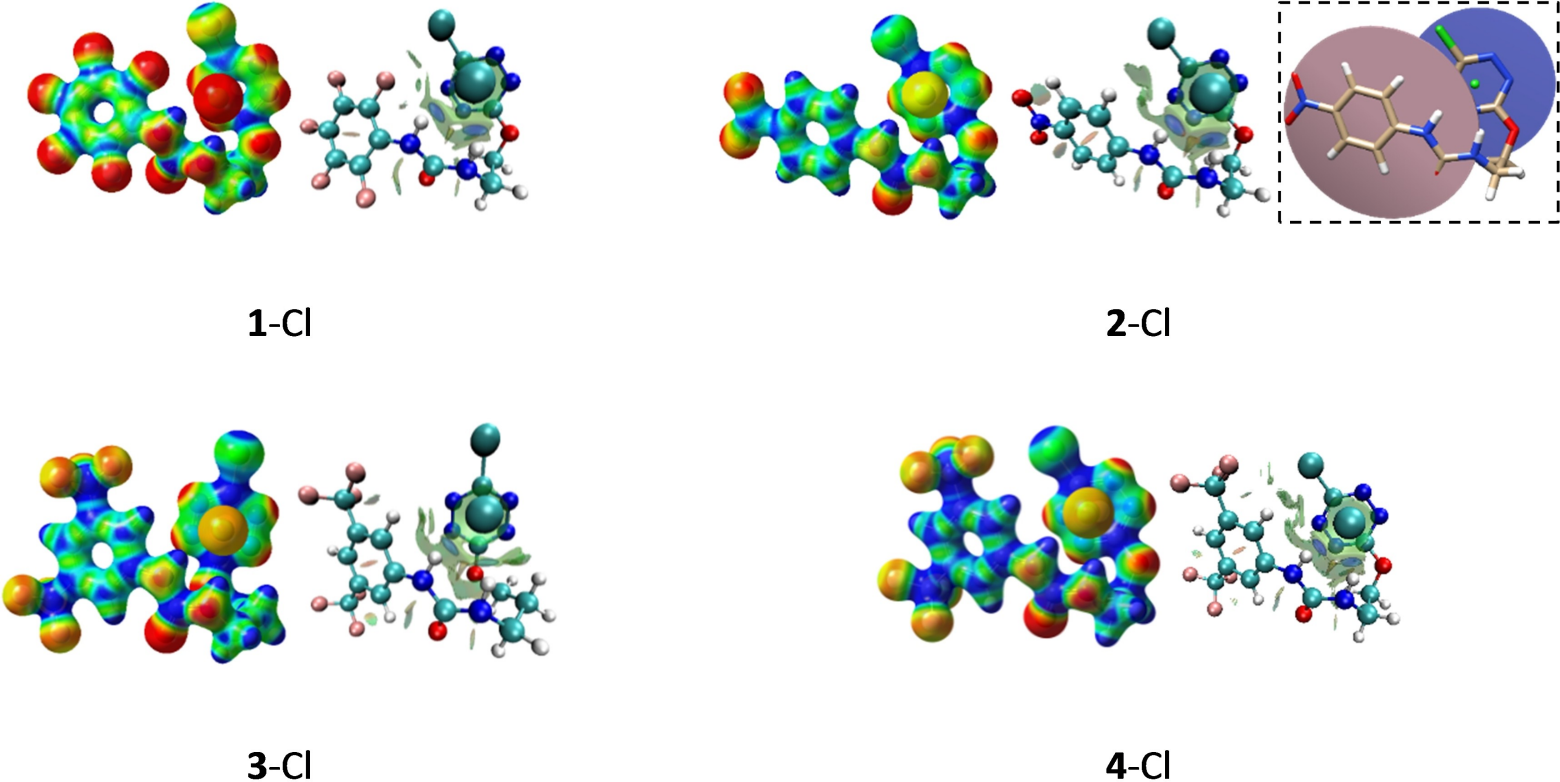
Molecular modelling results: ESP maps (Parameters : MO=0.02, isovalue=0.08, Laplacian=0) and NCIplot (Parameters : NCI Analysis^[34]^ on MultWFN,^[35]^ medium quality, isovalue : 0.45, material: translucent) (insert: Visualisation of planes).

In addition, the dual role and contribution of the two fragments in the formation of chloride anion complexes have been highlighted by a complementary theoretical approach. Indeed, the geometries of both receptors and the corresponding complexes have been optimized. Electrostatic potential surfaces (ESP) as well as Non‐Covalent Interactions plots (NCI‐Plot) have been calculated in order to visualize non covalent interactions in real space. As evidenced in Figure 6 (and *ESI §2*), the presence of the chloride anion enforces a peculiar geometry of the receptor within the complex. In ternary receptors **1**–**3** and **4**, the overall shape is governed by the relative spatial arrangement of both the urea and the tetrazine moieties. Planes defined by these two structural motifs almost parallel each other, maximizing H‐bond and anion‐π interaction with chloride anion (*see Insert*, Figure 6 *)*. All NCI‐Plots exhibit well‐defined dish‐shaped surfaces which account for effective weak interaction surfaces and a contribution of both urea and the tetrazine motifs. Significant weakening of interaction energy is visible when evolving from **4**‐Cl to **3**‐Cl (see Table 1 *)*. Main difference between **3**‐Cl and **4**‐Cl arises from the tridimensional arrangement of all fragments within the receptor. Indeed, the respective spatial orientation of the tetrazine and the urea motifs around the anion, sets the overall shape of the receptor and thus the spatial proximity and efficiency of weak interaction donor fragments. A shorter alkyl chain in receptor **4** (ethyl chain) compared to receptor **3** (propyl chain) results in a parallel relative orientation of the tetrazine and urea planes. The increase in chain length in receptor **3** allows more flexibility and induces a significant deformation of the respective orientation of these planes. The two planes no longer face each other, which leads to a modification of the distances between weak interaction donors within receptors **3** and **4**. Indeed, the evolution of distances between the anion and the urea motif on one hand, and the centroid of the tetrazine on the other hand (see SI) are the following: H_urea_‐Cl bonds evolve from 2.084 and 2.188 Å for **4**‐Cl, to 2.130 and 2.175 Å for **3**‐Cl. Cl‐centroid of tetrazine evolve from 3.12 Å for **4**‐Cl to 3.09 Å for **3**‐Cl. Both set of distances are in agreement with a decrease in the strength of the H‐bonds during an almost constant Cl‐tetrazine interaction moving from **4**‐Cl to **3**‐Cl.

## Conclusion

In this communication, the influence of three structural modifications compared to reference receptor **4** already published, on chloride binding was examined. Our approach focused on the modulation of the urea motif and the flexibility of the spacer, leading to the synthesis of three new anion receptors. In the gas phase, the interaction of chloride anion with both the tetrazine and the urea units was assessed .However, mass spectrometry experiments do not point a stronger affinity of a particular receptor. On the other hand, NMR spectroscopy, based on the observed downfield shifts of the urea protons, show the remarkable affinity of receptor **2**, indicating that the presence of a nitro group on the benzenic ring has a significant effect onto the chloride affinity. In the ground state, the H‐bond was determined as the major interaction and the following classification of receptors obtained as: **2**‐Cl >**4**‐Cl >**3**‐Cl >**1**‐Cl. Excited state studies by fluorescence spectroscopy showed the coexistence of two quenching mechanisms and corroborates the highest affinity of **2** for chloride anion. Finally, a molecular modelling approach supported the results obtained by experimental methods. Moreover theoretical results show that increasing the length of the spacer has a significant effect on the geometry of the receptor within the complex and subsequently onto the chloride affinity. Extensions to other anion geometries and receptors are currently under investigation and results will be reported in due time.

## Experimental Section

### *General procedure for the synthesis of 1, 2 and 3* according to a previously published procedure.^[24]^

5.6 mL of DMF, arylisocyanate derivative (2.39 mmol, 1.0 eq) and 151 μL of ethanolamine (2.49 mmol, 1.04 eq) were stirred at rt overnight. Volatiles were evaporated and the crude product was purified by flash chromatography eluting with a Cyclohexane/EtOAc 3/7 mixture to afford the desired 1‐(2‐hydroxyethyl)‐3‐(aryl)urea intermediate as white solids. The latter (1.0 eq), dichlorotetrazine (1.0 eq) and a magnetic stirrer were transfered into a round bottom flask under argon atmosphere. Distilled dichloromethane was added, followed by 2,4,6‐collidine (1.05 eq). The mixture was stirred at rt until disappearance of the starting material, followed by dilution with water and HCl 1 M solution, and extraction using ethyl acetate. Organic layers were combined, dried over MgSO4 and volatiles were evaporated. The crude product was directly purified by flash chromatography eluting with a mixture of Cyclohexane/EtOAc 8/2 to afford the target receptor

(2‐((6‐chloro‐1,2,4,5‐tetrazin‐3‐yl)oxy)ethyl)‐3‐(perfluorophenyl)urea **1** Yield: 23 %. ^1^H NMR (300 MHz, Acetone d6, 25 °C, TMS) δ 7.86 (broad s, 1H, ‐N‐*H*), 6.66 (broad s, 1H, −N−*H*), 4.77 (t, 2H, 3JH−H=6 Hz), 3.79 (q, 2H, 3JH−H=6 Hz). ^13^C NMR (75 MHz, Acetone d6, 25 °C, TMS) δ 167.9, 164.8, 155.4, 144.2 (large d, m, 1JC‐F=173 Hz, *Carom*‐F), 139.7 (large d, m, 1JC‐F=248 Hz, *Carom*‐F), 138.5 (large d, m, 1JC‐F=248 Hz, *Carom*‐F), 115.4 (t, m, 2JC‐F=14 Hz, *Carom*‐F), 70.3, 39.7. ^19^F NMR (282 MHz, MeOD, 25 °C) (not calibrated) δ 29.4‐29.3 (m, 2F), 15.1 (t, 1F, 2JC‐F=23 Hz), 11.2‐11.0 (m, 2F). UV‐Visible (acetonitrile) λmax (ϵ)=220 nm (3.67 AU), 265 nm (0.25 AU), 324 nm (0.47 AU), 511 nm (0.11 AU). Fluorescence (acetonitrile) λexc=511 nm, λem,max=563 nm. HRMS (ESI+‐TOF) *m/z [M+H]+* calcd for C_11_H_7_N_6_O_2_F_5_Cl 385.0239, found 385.0232. Rf 0.57 (eluent: Cyclohexane/EtOAc 1 : 1). Melting point 171.5–172.5 °C

1‐(2‐((6‐chloro‐1,2,4,5‐tetrazin‐3‐yl)oxy)ethyl)‐3‐(4‐nitrophenyl)urea **2** Yield: 33 %. ^1^H NMR (300 MHz, Acetone d6, 25 °C, TMS) δ 8.76 (broad s, 1H, N‐*H*), 8.15 (d, 2H, 3JH−H=9 Hz, −C‐*Harom*), 7.72 (d, 2H, 3JH−H=9 Hz, −C‐*Harom*), 6.48 (broad s, 1H, ‐N‐*H*), 4.80 (t, 2H, 3JH−H=5 Hz, ‐ CH_2_‐), 3.81 (q, 2H, 3JH−H=5 Hz, −CH_2−_). ^13^C NMR (75 MHz, Acetone d6, 25 °C, TMS) δ 168.0, 164.8, 155.5, 147.8, 142.4, 125.7, 118.1, 70.4, 39.4. UV‐Visible (Acetonitrile) λmax (ϵ)=510 nm (0.12 A.U.), 329 nm (3.70 A.U.), 219 nm (4.17 A.U.). Fluorescence (Acetonitrile) λexc=510 nm, λem,max=561 nm. HRMS (ESI+‐TOF) *m/z [M+NO3]‐* calcd for C_11_H_10_ClN_8_O_7_‐ 401.0361, found 401.0358. Rf 0.26 (eluent: Cyclohexane/EtOAc 6/4). Melting point 149.5–150 °C.

1‐(3,5‐bis(trifluoromethyl)phenyl)‐3‐(3‐((6‐chloro‐1,2,4,5‐tetrazin‐3‐yl)oxy)propyl)urea **3**. Yield: 53 %. ^1^H NMR (300 MHz, Acetonitrile d3, 25 °C, TMS) δ 7.98 (s, 2H, Harom), 7.72 (broad s, 1H, ‐N−H), 7.52 (s, 1H, Harom), 5.60 (broad s, 1H, −N−H), 4.71 (t, 2H, 3JH−H=6 Hz, −CH_2−_), 3.42 (q, 2H, 3JH−H=6 Hz, −CH_2−_), 2.12 (quint, 2H, 3JH−H=6 Hz, −CH_2−_). ^13^C NMR (75 MHz, Acetone d6, 25 °C, TMS) δ 168.0, 164.7, 155.8, 143.6, 133.0‐131.7 (q, 2 C, 2JC‐F=33 Hz, C−CF3), 129.9–119.1 (q, 2 C, 1JC‐F=270 Hz, ‐CF3), 118.4, 114.8, 69.2, 37.2, 30.1. ^19^F NMR (282 MHz, MeOD, 25 °C) (not calibrated) δ 113.9 (6F, −CF_3_). UV‐Visible (Acetonitrile) λmax (ϵ)=212 nm (3.72 AU), 249 nm (3.46 AU), 297 nm (0.64 AU), 324 nm (0.53 AU), 511 nm (0.12 AU). Fluorescence (Acetonitrile) λexc=511 nm, λexc=560 nm. HRMS (ESI+‐TOF) m/z [M+H]+ calcd for C_14_H_12_N_6_O_2_F_6_Cl 445.0627, found 445.0614. Rf 0.17 (eluent: Cyclohexane/EtOAc 3/7). Melting point 131.5–133 °C

### NMR Titration Experiments

^1^H NMR spectra were recorded with Bruker AV−I 300 MHz spectrometer at 298 K, referenced to TMS signal and were calibrated using residual proton in Acetone d6 (δ=2.05 ppm) or Acetonitrile d3 (δ=1.94 ppm), according to the literature.^[36]^ ^19^F NMR spectra were recorded with Bruker AV‐I 300 MHz spectrometer at 282 MHz and 298 K and were not calibrated. 13 C NMR spectra were recorded with a Bruker AV‐I 300 MHz spectrometer at 75 MHz and 298 K and were calibrated using Acetone d6 (δ=30.60 ppm). NMR titrations were performed by adding aliquots of a solution containing the anionic guest (0.09 M for NBu_4_Cl and **2** and 0.14 M for NBu_4_Cl and **3**, 0.14 M for NBu_4_Br, 0.81 M for NBu_4_I and 1.83 M for NBu_4_SCN) and the receptor (3.5 mM) in MeCN‐d_3_ to the NMR tube. After each addition, a ^1^H NMR and eventually a ^19^F spectrum were recorded.^[36]^

### Mass Spectrometry Experiments

Interactions of receptors with Cl^−^ occurring in the gas phase were studied with a 3D ion trap instrument (Bruker Amazon Speed ETD). Complexes were generated in the gas phase by electrospray. To this end, equimolar mixtures of receptors/NBu_4_Cl were prepared. Starting from 5 10–2 M stock solutions of receptors and NBu4Cl solubilized in acetonitrile (ACN) and purified water, respectively, 10–4 M mixtures of receptors/ NBu4Cl (90/10 ACN/H2O) were introduced in the electrospray source by a syringe pump (3 μL/min). Typical experimental conditions were as followed: Capillary voltage: −4500 V; End plate offset : −500 V; Dry gas: 4 L/min / Dry gas temperature: 180 °C, Nebuliser gas : 7.3 PSI ; Cap exit: −140 V; Trap Drive 49.5.

### Photophysical Experiments

UV‐Visible spectra were recorded at 25 °C on a Cary 400 (Agilent) double‐beam spectrometer using a 10 mm path quartz cell. Emission spectra were measured on a Fluoromax‐3 (Horiba) or a Fluorolog‐3 (Horiba) spectrofluorometer. An angle configuration of 90° was used. Optical density of the samples was checked to be less than 0.1 to avoid reabsorption artifacts. Fluorescence decay curves were obtained using an Edinburgh instrument LP920 laser flash photolysis spectrometer combined with an Nd:YAG laser (Continuum) doubled at 530 nm via non linear crystals. This second harmonic is optimized to pump an OPO. The fluorescence photons were detected at 90° through a long pass filter (GG385 SCHOTT) and a monochromator by means of a Hamamatsu R928 photomultiplier. The Levenberg‐Marquardt algorithm was used for non‐linear least square fit (tail fit) as implemented in the L900 software (Edinburgh instrument). In order to estimate the quality of the fit, the weighted residuals were calculated.

Preparation of samples: 10 mL of a stock solution of the anion receptor were prepared (10^−3^ mol/L) in acetonitrile. 2 mL of this solution was taken and diluted at 2.10^−4^ mol/L. 2 mL of this solution at 2.10^−4^ mol/L were introduced into a quartz cuvette. 2 mL of the stock solution was taken, desired amount of salt was added (135 mg for TBACl,) and diluted at 2.10^−4^ mol/L.

Binding constants were also determined using SPECFIT/32 ^TM^ Global Analysis System software.[^10^, ^11^] This software allows global analysis of equilibrium and kinetic systems with Expanded SVD and nonlinear regression modeling by the Levenberg‐Marquardt method.

### Molecular Modeling

Computed structures were preoptimized with a MM2 forcefield using Chem3D®. Then, optimizations were calculated at APFD/6‐31G+(d,p) calculation level using Gaussian® software without any solvent correction. Stationary points were verified by a harmonic vibrational frequencies calculation. None of the predicated geometry has any imaginary frequency implying that the optimized geometry of each of the molecules under study lay at a minimum local point on the potential energy surface. Effect of solvent (acetonitrile) on geometries was evaluated by proceeding optimization calculations at APFD/6‐31G+(d,p) by adding the Polarizable Continuum Model (PCM) using the integral equation formalism variant (IEFPCM). Once again, stationary points were checked by a harmonic vibrational frequencies′ calculation. None of the predicated geometry has any imaginary frequency implying that the optimized geometry of each of the molecules under study lay at a minimum local point on the potential energy surface.

Theoretical UV‐Visible spectra were calculated on optimized geometries structures by an energy calculation using time dependant DFT calculation at TD PBE0/6‐311+g(d,p)//APFD/6‐31+g(d,p) level and solving on 24 first singlet states. A standard solvation model (IEFPCM) for acetonitrile was used. PBE0 was chosen for evaluation of the absorption properties because it gives good estimate for the vertical transition values for a broad range of organic dyes.^[37]^

**Supporting Information**: Synthetic procedures, characterization data, mass spectrometry experiments, NMR titrations, photophysical data, computational data, Molecular graphics images were produced using the UCSF Chimera package from the Resource for Biocomputing, Visualization, and Informatics at the University of California, San Francisco (supported by NIH P41 RR‐01081).

## Conflict of interest

The authors declare no conflict of interest.

1

## Supporting information

As a service to our authors and readers, this journal provides supporting information supplied by the authors. Such materials are peer reviewed and may be re‐organized for online delivery, but are not copy‐edited or typeset. Technical support issues arising from supporting information (other than missing files) should be addressed to the authors.

Supporting Information

## Data Availability

The data that support the findings of this study are available in the supplementary material of this article.
