# Supplementary material for: Chloride Binding Modulated by Anion Receptors Bearing Tetrazine and Urea
Source: Chemphyschem. 2022 Oct 20;24(2):e202200524. doi: 10.1002/cphc.202200524 (PMC10091995; doi:10.1002/cphc.202200524)
Supplement: Supplementary file 1 — Supporting Information [file CPHC-24-0-s001.pdf]

ChemPhysChem

Supporting Information

Chloride Binding Modulated by Anion Receptors Bearing Tetrazine and Urea

Romain Plais, Guy Gouarin, Amélie Bournier, Olfa Zayene, Vanessa Mussard, Flavien Bourdreux, Jérome Marrot, Arnaud Brosseau, Anne Gaucher, Gilles Clavier, Jean-Yves Salpin, and Damien Prim*

Supplementary information

Table of contents

1. General procedures, Material and Instrumentation 2
   1.1 General experimental procedures and Materials 2
   1.2 Instrumentation 2
   1.3 Molecular modelling and software 3
2. Computational Data 3
   2.1 Coordinates of computed structures for **1** and **1-**Cl complexes 3
   2.2 Coordinates of computed structures for **2** and **2-**Cl complexes 7
   2.3 Coordinates of computed structures for **3** and **3-**Cl complexes 11
3. Synthetic procedures and characterization data 15
   3.1 General procedure A for the synthesis of **S1, S2** and **S3** 15
   3.2 General procedure B for the synthesis of **1, 2** and **3** 15
   3.3 Preparation of 1-(2-hydroxyethyl)-3-(perfluorophenyl)urea **S1** 16
   3.4 Preparation of 1-(2-hydroxyethyl)-3-(4-nitrophenyl)urea **S2** 19
   3.5 Preparation of 1-(3,5-bis(trifluoromethyl)phenyl)-3-(3-hydroxypropyl)urea **S3** 21
   3.6 Preparation of 1-(2-((6-chloro-1,2,4,5-tetrazin-3-yl)oxy)ethyl)-3-(perfluorophenyl)urea **1** 25
   3.7 Preparation of 1-(2-((6-chloro-1,2,4,5-tetrazin-3-yl)oxy)ethyl)-3-(4-nitrophenyl)urea **2** 30
   3.8 Preparation of 1-(3,5-bis(trifluoromethyl)phenyl)-3-(3-((6-chloro-1,2,4,5-tetrazin-3-yl)oxy)propyl)urea **3** 34
4. Mass Spectrometry Experiments 39
5. NMR Titrations 46
   5.1 Practical analysis procedure 46
   5.2 Titration of **1** with NBu_4Cl 47
   5.3 Titration of **2** with NBu_4Cl 50
   5.4 Titration of **3** with NBu_4Cl 52
6. Photophysical analysis and procedures 54
   6.1 General practical analysis procedure 54
   6.2 Determination of quantum yields 54
   6.3 Time dependant DFT analysis 56
   6.4 Titration of **1** with NBu_4Cl 64
   6.5 Titration of **2** with NBu_4Cl 69
   6.6 Titration of **3** with NBu_4Cl 74
   6.7 Stern-Volmer analysis 79
7. Bibliography 80

1. General procedures, Material and Instrumentation

1.1 General experimental procedures and Materials

Unless otherwise noted, all starting materials were obtained from commercial suppliers and used without purification. N,N-Dimethylformamide (100mL, Anhydrous, 99.8%) was purchased at Sigma-Aldrich. Dichloromethane was distilled over Sodium and under argon. For NMR titrations, deuterated acetonitrile (99.80% D) was purchased in 0.75mL precoated bulbs from Eurisotop®. For photophysical analysis, acetonitrile RS –SPECTROSOL – For optical spectroscopy was purchased from Carlo Erba®. Dichlorotetrazine reagent was synthesized according to a published procedure. [1]

Reaction progress was carried out using pre-coated TLC sheets ALUGRAM® Xtra SIL G/UV_{254} (0.20mm) from Macherey-Nagel® and visualized under 254 and 365 nm UV lamp from Fisher Bioblock Scientific®. Flash chromatography were proceeded using Silica 60M (0.04-0.063mm) for column chromatography silica gel from Macherey-Nagel®.

Crystals suitable for X-ray analysis were obtained by slow evaporation in an NMR tube of a saturated solution of the desired compound in deuterated acetone.

1.2 Instrumentation

^{1}H NMR spectra were recorded with Bruker AV-I 300MHz spectrometer at 298K, referenced to TMS signal and were calibrated using residual proton in Acetone d6 (δ=2.05ppm) or Acetonitrile d3 (δ=1.94ppm), according to the literature. [2] ^{19}F NMR spectra were recorded with Bruker AV-I 300MHz spectrometer at 282MHz and 298K and were not calibrated. ^{13}C NMR spectra were recorded with a Bruker AV-I 300MHz spectrometer at 75MHz and 298 K and were calibrated using Acetone d6 (δ = 30.60 ppm). [2] ^{1}H NMR spectroscopic data are reported as follow: chemical shift δ [parts per million] (multiplicity, coupling constants in Hertz, integration). Multiplicities are reported as follow: s = singlet, d = doublet, t = triplet, q = quadruplet, quint = quintuplet, sext = sextuplet, hept = heptuplet, dd = doublet of doublet, td = triplet of doublet, tt = triplet of triplet, ddd = doublet of doublet of doublet, m = multiplet. ^{13}C NMR spectroscopic data are reported in terms of chemical shifts δ [ppm] and when it is necessary multiplicity and coupling constant in Hertz.

To check the structure of the product obtained during the synthesis, high resolution mass spectra (HRMS) were obtained with a Waters Xevo QTOF instrument fitted with an electrospray ionization source (ESI+), using Leucine Enkephaline solution as internal calibrant.

Interactions of receptors with the various anions occurring in the gas phase were studied with a 3D ion trap instrument (Bruker Amazon Speed ETD). Complexes were generated in the gas phase by electrospray. To this end, equimolar mixtures of **receptors**/NBu_4Cl were prepared. Starting from 5 10^{-2}M stock solutions of **receptors** and NBu_4Cl solubilized in acetonitrile (ACN) and purified water, respectively, 10^{-4} M mixtures of **receptors**/ NBu_4Cl (90/10 ACN/H_2O) were introduced in the electrospray source by a syringe pump (3 µL/min). Typical experimental conditions were as followed: Capillary voltage: - 4500 V; End plate offset : -500 V; Dry gas: 4 L/min / Dry gas temperature: 180 °C, Nebuliser gas : 7.3 PSI ; Cap exit: -140 V; Trap Drive 49.5.

All spectra were recorded in the "Maximum Resolution mode"
MS analysis : ICC mode : "off" and acquisition time : manual.
MS^n analysis : ICC mode off / accumulation time 1 to 5 ms / Isolation window 1 to 7 Da / Fragmentation delay 40 ms/ amplitude of fragmentation : 0.20-1.0 depending on the ions.

UV-Visible spectra were recorded at 25°C on a Cary 400 (Agilent) double-beam spectrometer using a 10 mm path quartz cell.

Emission spectra were measured on a Fluoromax-3 (Horiba) or a Fluorolog-3 (Horiba) spectrofluorometer. An angle configuration of 90° was used. Optical density of the samples was checked to be less than 0.1 to avoid reabsorption artifacts.

Fluorescence decay curves were obtained using an Edinburgh instrument LP920 laser flash photolysis spectrometer combined with an Nd:YAG laser (Continuum) doubled at 530 nm via non linear crystals. This second harmonic is optimized to pump an OPO. The fluorescence photons were detected at 90° through a long pass filter (GG385 SCHOTT) and a monochromator by means of a Hamamatsu R928 photomultiplier. The Levenberg-Marquardt algorithm was used for non-linear least square fit (tail fit) as implemented in the L900 software (Edinburgh instrument). In order to estimate the quality of the fit, the weighted residuals were calculated.

1.3 Molecular modelling and software

All calculations were carried out using Gaussian 09® program:

Gaussian 09, Revision D.01, M. J. Frisch, G. W. Trucks, H. B. Schlegel, G. E. Scuseria, M. A. Robb, J. R. Cheeseman, G. Scalmani, V. Barone, B. Mennucci, G. A. Petersson, H. Nakatsuji, M. Caricato, X. Li, H. P. Hratchian, A. F. Izmaylov, J. Bloino, G. Zheng, J. L. Sonnenberg, M. Hada, M. Ehara, K. Toyota, R. Fukuda, J. Hasegawa, M. Ishida, T. Nakajima, Y. Honda, O. Kitao, H. Nakai, T. Vreven, J. A. Montgomery, Jr., J. E. Peralta, F. Ogliaro, M. Bearpark, J. J. Heyd, E. Brothers, K. N. Kudin, V. N. Staroverov, T. Keith, R. Kobayashi, J. Normand, K. Raghavachari, A. Rendell, J. C. Burant, S. S. Iyengar, J. Tomasi, M. Cossi, N. Rega, J. M. Millam, M. Klene, J. E. Knox, J. B. Cross, V. Bakken, C. Adamo, J. Jaramillo, R. Gomperts, R. E. Stratmann, O. Yazyev, A. J. Austin, R. Cammi, C. Pomelli, J. W. Ochterski, R. L. Martin, K. Morokuma, V. G. Zakrzewski, G. A. Voth, P. Salvador, J. J. Dannenberg, S. Dapprich, A. D. Daniels, O. Farkas, J. B. Foresman, J. V. Ortiz, J. Cioslowski, and D. J. Fox, Gaussian, Inc., Wallingford CT, 2013.

Computed structures were preoptimized with a MM2 forcefield using Chem3D®. Then, optimizations were calculated at APFD/6-31G+(d,p) calculation level using Gaussian® software without any solvent correction. Stationary points were verified by a harmonic vibrational frequencies calculation. None of the predicated geometry has any imaginary frequency implying that the optimized geometry of each of the molecules under study lay at a minimum local point on the potential energy surface.

Effect of solvent (acetonitrile) on geometries was evaluated by proceeding optimization calculations at APFD/6-31G+(d,p) by adding the Polarizable Continuum Model (PCM) using the integral equation formalism variant (IEFPCM). Once again, stationary points were checked by a harmonic vibrational frequencies' calculation. None of the predicated geometry has any imaginary frequency implying that the optimized geometry of each of the molecules under study lay at a minimum local point on the potential energy surface.

Theoretical UV-Visible spectra were calculated on optimized geometries structures by an energy calculation using time dependant DFT calculation at TD PBE0/6-311+g(d,p)//APFD/6-31+g(d,p) level and solving on 24 first singlet states. A standard solvation model (IEFPCM) for acetonitrile was used. PBE0 was chosen for evaluation of the absorption properties because it gives good estimate for the vertical transition values for a broad range of organic dyes. [3] We have verified previously that it is performing accurately on the tetrazine and urea receptors. [4] Electrostatic Potentials Surfaces (ESP) were thus calculated using Gaussview® software from optimized structures using a fine grid for Total Density and a medium grid of ESP. NCIplots were generated using the NCI method [5] implemented into the MultiWfn Software using a fine grid. [6] Visualization of NCIplots was performed using the Visual Molecular Dynamics VMD Software. [7]

2. Computational Data

Energies reported, unless noted, are expressed in Hartree.

2.1 Coordinates of computed structures for **1** and **1-**Cl complexes

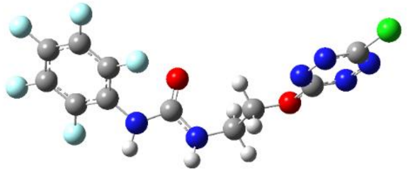

1 *(in vacuum)*

APFD/6-31+G(d,p)
Charge: 0
Spin Multiplicity: Singlet
Imaginary frequencies: 0
Electronic Energy (RAPFD): -1859.917737 Hartree
Zero-point correction= 0.188802 (Hartree/Particle)
Thermal correction to Energy= 0.211143
Thermal correction to Enthalpy= 0.212087
Thermal correction to Gibbs Free Energy= 0.132576
Sum of electronic and zero-point Energies= -1859.728935
Sum of electronic and thermal Energies= -1859.706594
Sum of electronic and thermal Enthalpies= -1859.705650
Sum of electronic and thermal Free Energies= -1859.785162

Symbol	X	Y	Z
C	3.97750500	1.84069400	-0.02868700
C	5.25323700	1.37575300	0.28275500
C	5.52467300	0.01118500	0.22805000
C	4.50790600	-0.87169700	-0.11502900
C	3.22306900	-0.43075200	-0.43257100
C	2.97975500	0.94448800	-0.39900500
N	2.22721100	-1.33973000	-0.79608100
C	1.03390600	-1.38667300	-0.05939900
N	0.15240800	-2.32423000	-0.53270700
O	0.81588100	-0.66955900	0.90130500
C	-1.13930300	-2.46834500	0.10388600
C	-2.08057800	-1.33560800	-0.28034500
O	-3.31497000	-1.60931600	0.40051300
C	-4.31209500	-0.75698500	0.24253600
N	-5.42582800	-1.07961300	0.93012200
N	-6.43826400	-0.26854800	0.81980000
C	-6.28257200	0.80847000	0.03385600
N	-5.19527400	1.12098900	-0.66108500
N	-4.16697700	0.30661200	-0.55450800
Cl	-7.62533000	1.87206000	-0.08754900
H	2.56344900	-2.21729800	-1.16971800
H	0.25412200	-2.64201100	-1.48535300
H	-1.56602600	-3.43424600	-0.18093400
H	-0.99325700	-2.46966100	1.18705900
H	-1.68098200	-0.36961400	0.04165000
H	-2.25914600	-1.30062400	-1.36117800
F	1.78655700	1.41960400	-0.75036300
F	3.72440400	3.14848000	0.00231600
F	6.21372100	2.23507500	0.61946500
F	6.74426300	-0.44233200	0.51915800
F	4.76652300	-2.18883800	-0.15200100

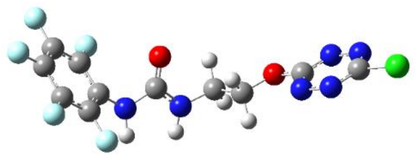

***1** (in acetonitrile)*

APFD/6-31+G(d,p), scrf=(iefpcm, solvent=acetonitrile)
Charge: 0
Spin Multiplicity: Singlet
Imaginary frequencies: 0
Electronic Energy (RAPFD): -1859.938910 Hartree
Zero-point correction= 0.188288 (Hartree/Particle)
Thermal correction to Energy= 0.210821
Thermal correction to Enthalpy= 0.211765
Thermal correction to Gibbs Free Energy= 0.131370
Sum of electronic and zero-point Energies= -1859.750621
Sum of electronic and thermal Energies= -1859.728089
Sum of electronic and thermal Enthalpies= -1859.727145
Sum of electronic and thermal Free Energies= -1859.807540

Symbol	X	Y	Z
C	-4.91326900	-0.24349700	-1.14019300
C	-5.33477200	1.01311500	-0.71747500
C	-4.71299100	1.61944400	0.36891800
C	-3.66161300	0.97161400	1.00553300
C	-3.22284000	-0.29192300	0.60459500

C	-3.88070100	-0.89200500	-0.47218800
N	-2.18198000	-0.93274800	1.27527400
C	-1.01858400	-1.29019200	0.59796000
N	-0.06649600	-1.85606000	1.38668900
O	-0.88762600	-1.12081700	-0.61309800
C	1.21414600	-2.23942100	0.83885800
C	2.14703600	-1.03977700	0.76977800
O	3.36948500	-1.52094600	0.17646500
C	4.35357700	-0.66574700	-0.00327500
N	5.45421100	-1.20369000	-0.56735500
N	6.46229000	-0.40669600	-0.77446300
C	6.32008500	0.87456300	-0.40978800
N	5.24238400	1.41068900	0.14923900
N	4.21843200	0.61523300	0.36260400
Cl	7.66008700	1.91233000	-0.69246200
H	-2.14050300	-0.79876100	2.27684100
H	-0.22816600	-1.97977200	2.37515600
H	1.64399100	-3.02089800	1.46982100
H	1.06541800	-2.65049500	-0.16315100
H	1.72603200	-0.24901400	0.14225000
H	2.36127100	-0.63461100	1.76323600
F	-3.54439300	-2.12329200	-0.86168800
F	-5.52299600	-0.83625400	-2.17082600
F	-6.33734500	1.63002300	-1.34612600
F	-5.11214100	2.82627000	0.77977300
F	-3.05498200	1.57813000	2.03448000

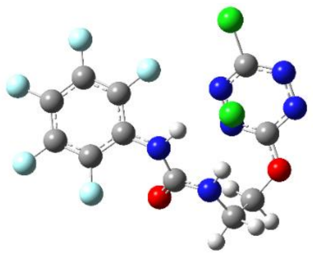

1-Cl *(in vacuum)*
APFD/6-31+G(d,p)
Charge: -1
Spin Multiplicity: Singlet
Imaginary frequencies: 0
Electronic Energy (RAPFD): -2320.146366 Hartree
Zero-point correction= 0.190057 (Hartree/Particle)
Thermal correction to Energy= 0.213490
Thermal correction to Enthalpy= 0.214434
Thermal correction to Gibbs Free Energy= 0.134883
Sum of electronic and zero-point Energies= -2319.956309
Sum of electronic and thermal Energies= -2319.932876
Sum of electronic and thermal Enthalpies= -2319.931932
Sum of electronic and thermal Free Energies= -2320.011483

Symbol	X	Y	Z
C	-4.04028300	-0.65069500	-0.13890300
C	-4.16515500	0.71829700	-0.34445000
C	-3.06425500	1.53986700	-0.12830800
C	-1.84847900	0.98641800	0.25625400
C	-1.68599600	-0.39414100	0.44591500
C	-2.82805100	-1.19263000	0.27598700
N	-0.46920300	-0.90898400	0.83698800
C	0.09112800	-2.01780500	0.20525700
N	1.29046000	-2.36838000	0.75415400
O	-0.44249700	-2.61383000	-0.72797900
C	2.12764600	-3.30254600	0.05237300
C	2.78065800	-2.70979900	-1.19014000
O	3.70374000	-1.66428600	-0.82704900

C	3.28892300	-0.40504500	-0.81113600
N	2.06502600	-0.07679900	-1.23304500
N	1.68192500	1.16362300	-1.05589200
C	2.55343900	1.96731800	-0.45712200
N	3.85766200	1.71384900	-0.31213400
N	4.24983200	0.48834400	-0.51237700
Cl	2.01164300	3.54246500	-0.03044600
H	0.19703000	-0.29870100	1.34955100
H	1.73088100	-1.67666200	1.38118100
H	2.90107000	-3.65334500	0.74359400
H	1.52400000	-4.16240300	-0.26010200
H	2.02183300	-2.32801800	-1.87707900
H	3.40086700	-3.45628300	-1.69590700
Cl	2.06685800	0.26568400	2.22013000
F	-0.81302400	1.80237700	0.45493200
F	-3.17741600	2.86315900	-0.31063000
F	-5.33947200	1.24325500	-0.72866300
F	-5.11123400	-1.44399100	-0.29729800
F	-2.80190900	-2.49315400	0.57456300

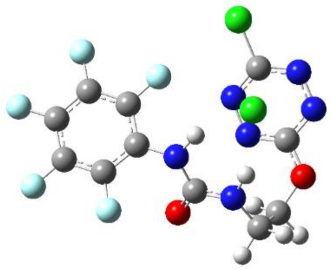

***1-Cl** (in acetonitrile)*
APFD/6-31+G(d,p), scrf=(iefpcm, solvent=acetonitrile)
Charge: -1
Spin Multiplicity: Singlet
Imaginary frequencies: 0
Electronic Energy (RAPFD): -2320.222590 Hartree
Zero-point correction= 0.189884 (Hartree/Particle)
Thermal correction to Energy= 0.213592
Thermal correction to Enthalpy= 0.214536
Thermal correction to Gibbs Free Energy= 0.134048
Sum of electronic and zero-point Energies= -2320.032706
Sum of electronic and thermal Energies= -2320.008998
Sum of electronic and thermal Enthalpies= -2320.008054
Sum of electronic and thermal Free Energies= -2320.088542

Symbol	X	Y	Z
C	-4.05184800	-0.68410100	-0.12555200
C	-4.19050000	0.68719200	-0.30719500
C	-3.09235800	1.51523500	-0.10420300
C	-1.86652400	0.96397900	0.24681300
C	-1.69251900	-0.41387100	0.41886200
C	-2.82658300	-1.21932200	0.25616000
N	-0.46153600	-0.92826900	0.79107400
C	0.12633300	-1.99386600	0.11888600
N	1.31561000	-2.36637300	0.66565800
O	-0.39280900	-2.54992100	-0.85054800
C	2.17362300	-3.30471100	-0.01690100
C	2.90833000	-2.69758500	-1.20392700
O	3.76869900	-1.62032800	-0.76021800
C	3.32068800	-0.38005800	-0.76045500
N	2.10303900	-0.07899500	-1.22722100
N	1.69714600	1.15931100	-1.09705500
C	2.53115800	2.00349900	-0.50100400

N	3.80849500	1.75219500	-0.20303700
N	4.22738600	0.52780800	-0.34955900
Cl	1.95693100	3.59372400	-0.18955700
H	0.16589300	-0.33914200	1.35514800
H	1.72626600	-1.74071100	1.36281700
H	2.89472000	-3.69121100	0.70850200
H	1.57627200	-4.14628300	-0.38061200
H	2.21363000	-2.32855900	-1.96001500
H	3.58967400	-3.42449300	-1.64990700
Cl	2.02024600	0.25211700	2.39780000
F	-0.82643600	1.78533700	0.43277900
F	-3.21435600	2.83769700	-0.26474800
F	-5.37150600	1.20593200	-0.65880500
F	-5.11268300	-1.48504600	-0.28011600
F	-2.77006000	-2.52892000	0.51779500

2.2 Coordinates of computed structures for **2** and **2-**Cl complexes

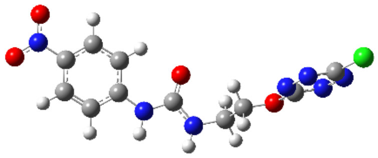

2 *(in vacuum)*

APFD/6-31+G(d,p)
Charge: 0
Spin Multiplicity: Singlet
Imaginary frequencies: 0
Electronic Energy (RAPFD): -1568.453193 Hartree
Zero-point correction= 0.232468 (Hartree/Particle)
Thermal correction to Energy= 0.252918
Thermal correction to Enthalpy= 0.253862
Thermal correction to Gibbs Free Energy= 0.177501
Sum of electronic and zero-point Energies= -1568.220725
Sum of electronic and thermal Energies= -1568.200275
Sum of electronic and thermal Enthalpies= -1568.199331
Sum of electronic and thermal Free Energies= -1568.275692

Symbol	X	Y	Z
C	4.61564200	1.07934800	0.75397200
C	5.72917500	0.77035100	-0.02191500
C	5.71121400	-0.29562600	-0.91866100
C	4.55877800	-1.05351200	-1.03280400
C	3.42192600	-0.75800700	-0.25821900
C	3.45928800	0.31923000	0.64282100
N	2.30326600	-1.56730000	-0.44739000
C	1.08664000	-1.51130100	0.21765200
N	0.16420700	-2.41527500	-0.25896600
O	0.84513400	-0.75810300	1.15034200
C	-1.15893100	-2.45395700	0.32580900
C	-2.01930800	-1.29733500	-0.16212800
O	-3.29773700	-1.46856100	0.46806600
C	-4.23370800	-0.56915000	0.21904300
N	-5.39764100	-0.79165800	0.86143900
N	-6.35215300	0.07057900	0.65997700
C	-6.09245300	1.09641300	-0.16616800
N	-4.95475700	1.30998900	-0.81654800
N	-3.98473900	0.44294600	-0.61802300
Cl	-7.36018900	2.22866600	-0.40767300

H	4.53796600	-1.88608200	-1.73308900
H	2.59091400	0.54347000	1.24716100
H	2.43375000	-2.32535300	-1.10123400
H	0.29163600	-2.83510600	-1.16739000
H	-1.62573200	-3.41038700	0.07348400
H	-1.05986100	-2.40224800	1.41298800
H	-1.58426400	-0.33727900	0.12998000
H	-2.14385400	-1.31082400	-1.25104100
H	6.59237900	-0.51558400	-1.51086700
H	4.66257900	1.91450100	1.44421400
N	6.93808900	1.57311200	0.10445000
O	6.92226000	2.50435700	0.90213200
O	7.89837200	1.26756900	-0.59613500

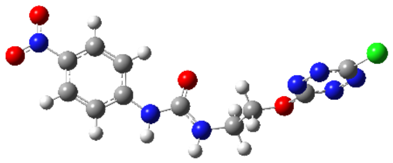

2 *(in acetonitrile)*

APFD/6-31+G(d,p), scrf=(iefpcm,solvent=acetonitrile)
Charge: 0
Spin Multiplicity: Singlet
Imaginary frequencies: 0
Electronic Energy (RAPFD): -1568.480746 Hartree
Zero-point correction= 0.232335 (Hartree/Particle)
Thermal correction to Energy= 0.252708
Thermal correction to Enthalpy= 0.253652
Thermal correction to Gibbs Free Energy= 0.177358
Sum of electronic and zero-point Energies= -1568.248411
Sum of electronic and thermal Energies= -1568.228038
Sum of electronic and thermal Enthalpies= -1568.227094
Sum of electronic and thermal Free Energies= -1568.303388

Symbol	X	Y	Z
C	4.79718100	0.92537500	0.88222200
C	5.83671100	0.71196100	-0.02351300
C	5.69752900	-0.19183400	-1.07994400
C	4.50844700	-0.88044100	-1.22167000
C	3.44397700	-0.67992900	-0.31733400
C	3.60357200	0.23555000	0.74142200
N	2.29112700	-1.41431100	-0.53657100
C	1.10898200	-1.39580100	0.19283600
N	0.16005100	-2.24856400	-0.28707700
O	0.92935500	-0.68960000	1.18485400
C	-1.14055600	-2.32645500	0.33673400
C	-2.04691300	-1.21436300	-0.17018700
O	-3.28994200	-1.35976900	0.54498200
C	-4.26653300	-0.52005700	0.27368500
N	-5.38940900	-0.73071300	0.99059500
N	-6.39467800	0.06420500	0.76264200
C	-6.22802500	1.01968900	-0.16170800
N	-5.12495500	1.23711900	-0.86708600
N	-4.10353500	0.44075600	-0.64465000
Cl	-7.56698300	2.05625400	-0.45246900
H	4.39579000	-1.58478500	-2.04165800
H	2.79376300	0.39428100	1.43938300
H	2.32800200	-2.03920400	-1.33089200

H	0.31323500	-2.76548200	-1.14027000
H	-1.57573100	-3.30385100	0.11507300
H	-1.02089200	-2.24160200	1.41987100
H	-1.61994100	-0.22991900	0.04131500
H	-2.23393800	-1.30187800	-1.24470300
H	6.51401800	-0.34699500	-1.77571700
H	4.92634100	1.63074200	1.69542400
N	7.07505700	1.43501500	0.13196000
O	7.17819400	2.22107200	1.07514500
O	7.97442000	1.23400700	-0.68620700

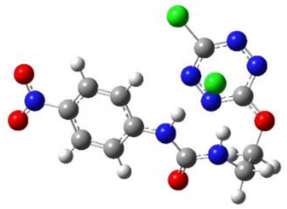

2-Cl *(in vacuum)*

APFD/6-31+G(d,p)
Charge: -1
Spin Multiplicity: Singlet
Imaginary frequencies: 0
Electronic Energy (RAPFD): -2028.693140 Hartree
Zero-point correction= 0.234508 (Hartree/Particle)
Thermal correction to Energy= 0.255764
Thermal correction to Enthalpy= 0.256709
Thermal correction to Gibbs Free Energy= 0.181135
Sum of electronic and zero-point Energies= -2028.458632
Sum of electronic and thermal Energies= -2028.437376
Sum of electronic and thermal Enthalpies= -2028.436431
Sum of electronic and thermal Free Energies= -2028.512005

Symbol	X	Y	Z
C	-3.47324400	-1.45607600	-0.49114200
C	-3.99362100	-0.23907700	-0.04308000
C	-3.20571100	0.64237600	0.70666500
C	-1.90511900	0.29679000	1.00980500
C	-1.36277300	-0.93417600	0.57163100
C	-2.16879900	-1.80789400	-0.19023300
N	-0.06916400	-1.19994300	0.93435400
C	0.76661200	-2.18823700	0.43766800
N	2.03899700	-2.05719400	0.92705800
O	0.42675800	-3.08091700	-0.34001200
C	3.11745200	-2.70864600	0.23422600
C	3.44106000	-2.04754300	-1.10235500
O	3.91328900	-0.69925500	-0.90729900
C	3.04780400	0.30596100	-0.92766300
N	1.76066300	0.10308400	-1.21749600
N	0.94254700	1.11422700	-1.05134600
C	1.48556800	2.24310400	-0.61305700
N	2.79306400	2.52240300	-0.62632700
N	3.60906000	1.52316700	-0.80212000
Cl	0.41363900	3.51881000	-0.18877200
N	-5.35356800	0.11272400	-0.35755400
H	-1.27291600	0.97645000	1.57594300

H	-1.74694100	-2.74193800	-0.53777100
H	0.42348600	-0.44939100	1.45542300
H	2.24804700	-1.18892100	1.44267600
H	4.00049100	-2.69168300	0.88108100
H	2.84739100	-3.75283000	0.04035900
H	2.56347000	-2.05106700	-1.75341700
H	4.27365100	-2.55675800	-1.59690500
Cl	1.91715400	0.81508700	2.19245500
H	-4.10383700	-2.11777300	-1.07492000
H	-3.62399400	1.58801600	1.03279700
O	-5.78955200	1.18909500	0.05884100
O	-6.02285900	-0.67881200	-1.02761100

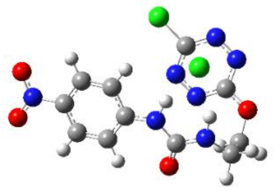

***2-Cl** (in acetonitrile)*

APFD/6-31+G(d,p), scrf=(iefpcm,solvent=acetonitrile)
Charge: -1
Spin Multiplicity: Singlet
Imaginary frequencies: 0
Electronic Energy (RAPFD): -2028.766867 Hartree
Zero-point correction= 0.234081 (Hartree/Particle)
Thermal correction to Energy= 0.255625
Thermal correction to Enthalpy= 0.256569
Thermal correction to Gibbs Free Energy= 0.179942
Sum of electronic and zero-point Energies= -2028.532787
Sum of electronic and thermal Energies= -2028.511242
Sum of electronic and thermal Enthalpies= -2028.510298
Sum of electronic and thermal Free Energies= -2028.586926

Symbol	X	Y	Z
C	-3.39823400	-1.51380300	-0.49604500
C	-3.93849700	-0.31713700	-0.01855200
C	-3.17528000	0.55490700	0.76614500
C	-1.87088200	0.22080800	1.06679600
C	-1.30326900	-0.98363500	0.59191400
C	-2.08932900	-1.85099700	-0.19497300
N	0.00126700	-1.23160200	0.94719800
C	0.86938300	-2.16531100	0.40005500
N	2.14192900	-2.01619800	0.87245400
O	0.54125700	-3.03498400	-0.40961300
C	3.24812200	-2.62677400	0.17274800
C	3.56727500	-1.93052600	-1.14518300
O	3.93428000	-0.54788500	-0.91165600
C	3.00908000	0.39210900	-0.93412900
N	1.73588200	0.11238000	-1.23380500
N	0.85754700	1.07400200	-1.09076000
C	1.31616600	2.24464300	-0.66574100
N	2.60532500	2.58530800	-0.58118300
N	3.48476000	1.63687700	-0.73149100
Cl	0.15689400	3.46127900	-0.30709800
N	-5.29794700	0.02425000	-0.33786400
H	-1.26368700	0.89479600	1.66509800
H	-1.66099700	-2.77359300	-0.56214500

H	0.45955900	-0.49196300	1.49849400
H	2.33538600	-1.18233500	1.43257700
H	4.12009000	-2.60262700	0.83147700
H	3.01560800	-3.67404000	-0.04229700
H	2.72327900	-1.97575600	-1.83541200
H	4.45043200	-2.37014800	-1.61190500
Cl	1.91233900	0.86027300	2.33597900
H	-4.00858500	-2.17589900	-1.10006800
H	-3.60729000	1.48223700	1.12476200
O	-5.75391600	1.08563600	0.09651600
O	-5.95278400	-0.75750400	-1.03294800

2.3 Coordinates of computed structures for **3** and **3-**Cl complexes

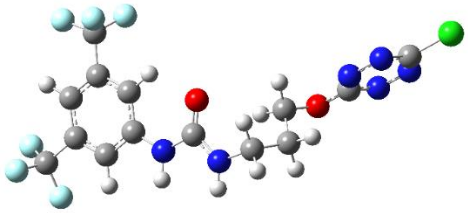

***3** (in vacuum)*

APFD/6-31+G(d,p)
Charge: 0
Spin Multiplicity: Singlet
Imaginary frequencies: 0
Electronic Energy (RAPFD): -2077.036443 Hartree
Zero-point correction= 0.267432 (Hartree/Particle)
Thermal correction to Energy= 0.293980
Thermal correction to Enthalpy= 0.294924
Thermal correction to Gibbs Free Energy= 0.201762
Sum of electronic and zero-point Energies= -2076.769011
Sum of electronic and thermal Energies= -2076.742463
Sum of electronic and thermal Enthalpies= -2076.741519
Sum of electronic and thermal Free Energies= -2076.834681

Symbol	X	Y	Z
C	4.87896500	-0.56445900	0.45191400
C	3.78984700	-1.35144900	0.10611100
C	2.62022700	-0.76028800	-0.39427700
C	2.56851100	0.63017300	-0.54182900
C	3.67872500	1.39320600	-0.18524600
C	4.84281200	0.82148000	0.31186900
N	1.56253200	-1.61405100	-0.71393000
C	0.31468100	-1.26694700	-1.21190500
N	-0.52179200	-2.33840800	-1.38930200
O	-0.01618200	-0.11738700	-1.47791300
C	-1.86394900	-2.15613100	-1.91105200
C	-2.93603500	-1.99738300	-0.83256600
C	-2.74706600	-0.72475100	-0.01979600
O	-3.69400600	-0.65388200	1.06994700
C	-4.92888300	-0.26620900	0.81334600
N	-5.30039100	0.04848500	-0.43369900
N	-6.53759400	0.46025800	-0.60834300
C	-7.30928100	0.51786600	0.47021600
N	-6.95407100	0.16917200	1.71617000
N	-5.73003900	-0.23477900	1.89905500
Cl	-8.92154600	1.07068900	0.25486900
C	6.14441400	-1.22513200	0.93196400
F	6.99725400	-1.46123700	-0.09215100
F	5.90410900	-2.41715400	1.52057600
F	6.80544400	-0.46169700	1.82370000

C	3.57335900	2.88861600	-0.35029600
F	2.59595400	3.40319400	0.42827900
F	3.27460700	3.22582400	-1.62332600
F	4.71731300	3.52272900	-0.02587600
H	3.84960500	-2.42915600	0.23448500
H	1.67350800	1.09740700	-0.93161600
H	5.69409200	1.43273600	0.58668600
H	1.76498300	-2.59760200	-0.61410500
H	-0.28081700	-3.24110800	-1.00893700
H	-1.83787400	-1.27284500	-2.55501700
H	-2.10119200	-3.01748000	-2.54371000
H	-2.92373500	-2.86208300	-0.15601500
H	-3.91735300	-1.97586800	-1.32070900
H	-2.83377900	0.16817800	-0.64453200
H	-1.78026500	-0.70496500	0.48322000

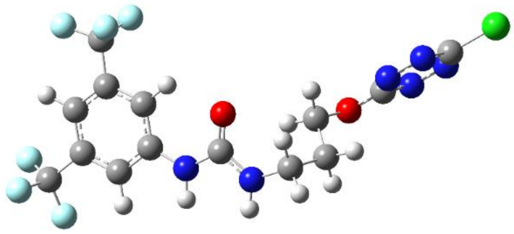

3 *(in acetonitrile)*

APFD/6-31+G(d,p) IEPFCM=(solvent=acetonitrile)
Charge: 0
Spin Multiplicity: Singlet
Imaginary frequencies: 0
Electronic Energy (RAPFD): -2077.059447 Hartree
Zero-point correction= 0.267592 (Hartree/Particle)
Thermal correction to Energy= 0.293919
Thermal correction to Enthalpy= 0.294863
Thermal correction to Gibbs Free Energy= 0.203376
Sum of electronic and zero-point Energies= -2076.791855
Sum of electronic and thermal Energies= -2076.765528
Sum of electronic and thermal Enthalpies= -2076.764584
Sum of electronic and thermal Free Energies= -2076.856071

Symbol	X	Y	Z
C	4.86625200	-0.56375200	0.45524700
C	3.78285400	-1.35391100	0.10062900
C	2.61315900	-0.76381100	-0.40876500
C	2.56676700	0.62821500	-0.55425400
C	3.67460200	1.39235200	-0.19085700
C	4.83557200	0.82386200	0.31570600
N	1.56166300	-1.61346200	-0.73328800
C	0.32048600	-1.27150000	-1.24866200
N	-0.52073800	-2.33088700	-1.39626200
O	0.00758500	-0.11704400	-1.55142500
C	-1.86096500	-2.17125900	-1.92740300
C	-2.93581000	-2.03715800	-0.84741300
C	-2.74133600	-0.78104400	-0.01437300
O	-3.70446700	-0.71349200	1.06965800
C	-4.92372100	-0.29133800	0.81519600
N	-5.28136900	0.08439000	-0.42110900
N	-6.51012200	0.51674000	-0.58670900
C	-7.29548200	0.53836200	0.48331000
N	-6.95179900	0.14931800	1.71730100
N	-5.73444800	-0.27736900	1.89427100
Cl	-8.90322600	1.10790300	0.26969800
C	6.12857200	-1.21519100	0.95071100
F	7.03254700	-1.37706200	-0.04839300

F	5.91010900	-2.43879200	1.47545700
F	6.74016200	-0.47681500	1.90254100
C	3.56573200	2.88465200	-0.35034300
F	2.60907600	3.40641900	0.45610200
F	3.22597800	3.23190700	-1.61437300
F	4.71492100	3.52653700	-0.06052200
H	3.83981500	-2.43153200	0.22272900
H	1.67343900	1.09486200	-0.94717600
H	5.68568100	1.43459600	0.59540700
H	1.73745600	-2.59530800	-0.57196800
H	-0.25592300	-3.24214800	-1.05076500
H	-1.85459300	-1.28872300	-2.57231800
H	-2.08211600	-3.03882800	-2.55540100
H	-2.91723000	-2.91463800	-0.18968500
H	-3.91631700	-2.01151400	-1.33649700
H	-2.81522500	0.12652700	-0.61839900
H	-1.78262800	-0.78508800	0.50423600

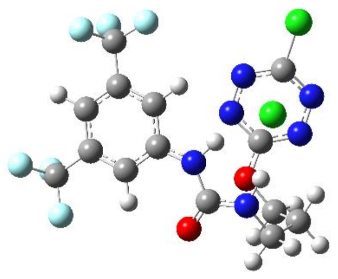

***3-Cl** (in vacuum)*
APFD/6-31+G(d,p)
Charge: -1
Spin Multiplicity: Singlet
Imaginary frequencies: 0
Electronic Energy (RAPFD): -2537.271871 Hartree
Zero-point correction= 0.269808 (Hartree/Particle)
Thermal correction to Energy= 0.297196
Thermal correction to Enthalpy= 0.298141
Thermal correction to Gibbs Free Energy= 0.206012
Sum of electronic and zero-point Energies= -2537.002063
Sum of electronic and thermal Energies= -2536.974674
Sum of electronic and thermal Enthalpies= -2536.973730
Sum of electronic and thermal Free Energies= -2537.065858

Symbol	X	Y	Z
C	-2.34750300	1.43463600	0.40569500
C	-1.17608000	0.83762400	0.84483100
C	-1.04494000	-0.56359700	0.81566100
C	-2.11263200	-1.33837700	0.33288700
C	-3.27320600	-0.70445400	-0.10098600
C	-3.41671300	0.67933300	-0.07493400
N	0.13969700	-1.09105700	1.27883800
C	0.61654300	-2.37713100	1.08351700
O	-0.02038500	-3.28783100	0.55097700
N	1.88957100	-2.51546500	1.57029900
C	2.74726700	-3.54332500	1.03313600
C	3.59867700	-3.04392400	-0.14013600
C	2.80792100	-2.72126900	-1.40265100
O	1.88493500	-1.62570300	-1.25603100
C	2.34799200	-0.38905000	-1.24073800
N	1.38784800	0.55590100	-1.20437200
N	1.78382900	1.77749200	-1.00548200
C	3.10183100	1.97511000	-0.88054900

N	4.04185400	1.10390600	-1.22141300
N	3.64872800	-0.13510500	-1.41177800
Cl	3.61196600	3.55711300	-0.43828800
C	-2.49414400	2.92936200	0.42408100
F	-3.58589400	3.31580000	1.14028700
F	-1.43549200	3.56301900	0.95653700
F	-2.67576400	3.43524300	-0.82238200
C	-4.41274300	-1.51766000	-0.64475800
F	-4.57825300	-1.33232500	-1.98107800
F	-5.59889900	-1.17308700	-0.07650800
F	-4.26186200	-2.84292600	-0.45467500
H	-0.34559300	1.44424600	1.19410800
H	-2.00840500	-2.41441300	0.29421100
H	-4.32991400	1.15600600	-0.41513000
H	0.85802200	-0.40783800	1.57505800
H	2.35741100	-1.63628400	1.83193700
H	3.40961600	-3.90401600	1.83020200
H	2.11140000	-4.38002800	0.72555700
H	4.17879600	-2.16490700	0.16571400
H	4.32409900	-3.82377100	-0.41540500
H	3.48316500	-2.49762000	-2.23541300
H	2.15323700	-3.55362100	-1.67307800
Cl	2.75409000	0.50086600	1.91652800

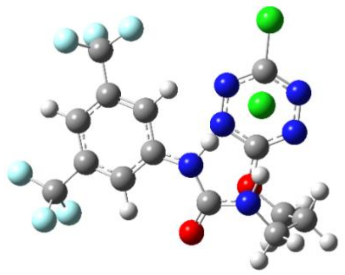

3-Cl *(in acetonitrile)*
APFD/6-31+G(d,p), scrf=(IEFPCM,solvent=acetonitrile)
Charge: -1
Spin Multiplicity: Singlet
Imaginary frequencies: 0
Electronic Energy (RAPFD): -2537.343673 Hartree
Zero-point correction= 0.268886 (Hartree/Particle)
Thermal correction to Energy= 0.296673
Thermal correction to Enthalpy= 0.297618
Thermal correction to Gibbs Free Energy= 0.203888
Sum of electronic and zero-point Energies= -2537.074787
Sum of electronic and thermal Energies= -2537.047000
Sum of electronic and thermal Enthalpies= -2537.046056
Sum of electronic and thermal Free Energies= -2537.139786

Symbol	X	Y	Z
C	-2.43465900	1.30312200	0.40617300
C	-1.22526100	0.80687500	0.86132600
C	-0.97126200	-0.57784200	0.83045500
C	-1.96246800	-1.43653500	0.34076700
C	-3.16955200	-0.90216900	-0.11176700
C	-3.43177100	0.46058100	-0.09216100
N	0.26346600	-0.99130500	1.29206700
C	0.86231500	-2.22642200	1.11046500
O	0.28908700	-3.20666700	0.62505500
N	2.15365900	-2.23503900	1.54928500
C	3.08849700	-3.25693200	1.12444600
C	3.99845700	-2.78486700	-0.01248900

C	3.31207000	-2.62063000	-1.35967300
O	2.19543300	-1.69451000	-1.34318600
C	2.42340300	-0.39999200	-1.28549600
N	1.30204100	0.34966400	-1.30612500
N	1.44583400	1.63271500	-1.15089700
C	2.69251700	2.09487200	-1.00199300
N	3.80449200	1.38543100	-1.13643700
N	3.66990100	0.08715900	-1.28128800
Cl	2.86221300	3.77908400	-0.70090200
C	-2.69486500	2.78315900	0.41288500
F	-3.83082300	3.09165000	1.08592600
F	-1.69831800	3.49158100	0.97889600
F	-2.85854700	3.26884300	-0.84321300
C	-4.19707200	-1.86530100	-0.63803200
F	-5.33524300	-1.25675700	-1.02961700
F	-4.54541000	-2.79056100	0.28980600
F	-3.73595300	-2.56018700	-1.70728300
H	-0.45855400	1.48214400	1.22836300
H	-1.77675400	-2.50221200	0.30860400
H	-4.37452100	0.85768700	-0.45030500
H	0.89433800	-0.24108300	1.60611000
H	2.54816700	-1.33277000	1.82178000
H	3.70513100	-3.54871800	1.98153100
H	2.50755800	-4.13287800	0.82354800
H	4.49864400	-1.85204400	0.27283400
H	4.79141800	-3.52909700	-0.16317600
H	4.02032100	-2.29466200	-2.12666000
H	2.84403400	-3.55411700	-1.67735200
Cl	2.59336800	0.97316200	2.10522300

3. Synthetic procedures and characterization data

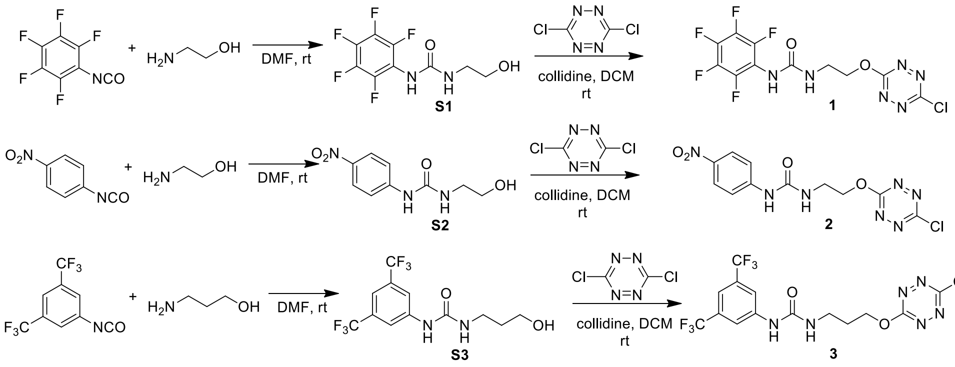

*Figure S1 : General procedure for the synthesis of **1, 2, 3, S1**, **S2** and **S3** compounds*

3.1 General procedure A for the synthesis of **S1, S2** and **S3**

Inspired by our previously published procedure.[4]

Solids (if any) and a magnetic stirrer were added to a round bottom flask under argon atmosphere, followed by dry N,N-dimethylformamide (DMF). Then, liquid reagents were added dropwise to the solution cooled down to 0°C using an ice-bath. Mixture was stirred for a night. Then, the mixture was diluted with water and HCl 1M solution, followed by extraction using ethyl acetate. Organic layers were gathered, dried over MgSO4 and volatiles were evaporated. The crude product was directly purified by flash chromatography.

3.2 General procedure B for the synthesis of **1, 2** and **3**

Inspired by our previously published procedure.[4]

Compound **S** (1.0eq), dichlorotetrazine (1.0eq) and a magnetic stirrer were added to a round bottom flask under argon atmosphere. Distilled dichloromethane was added to the flask, followed by 2,4,6-collidine (1.05eq). The mixture was stirred at rt. Volatiles was evaporated and the crude product was directly purified by flash chromatography.

3.3 Preparation of 1-(2-hydroxyethyl)-3-(perfluorophenyl)urea **S1**

General procedure A : 5.6 mL of DMF, 312µL of pentafluorophenylisocyanate (2.39mmol, 1.0eq) and 151µL of ethanolamine (2.49mmol, 1.04eq) were stirred at rt for a night. The product was eluted by a Cyclohexane/EtOAc 3/7 mixture to afford 447mg of desired 1-(2-hydroxyethyl)-3-(perfluorophenyl)urea **S1** as a white solid. Yield: 447mg, 69%.

^{1}H NMR (300MHz, Acetone d6, 25°C, TMS) δ 7.83 (broad s, 1H, -N-*H*), 6.36 (broad s, 1H, -N-*H*), 4.03 (broad s, 1H, -O-*H*), 3.62 (q, 2H, $^{3}J_{H\text{-}H}$= 5Hz, -C*H*$_2$-), 3.32 (q, 2H, $^{3}J_{H\text{-}H}$= 6Hz, -C*H*$_2$-).

^{13}C NMR (75MHz, Acetone d6, 25°C, TMS) δ 155.70 (s, 1C, *C*=O), 144.2 (large d, m, $^{1}J_{C\text{-}F}$ = 243 Hz, *C*$_{arom}$-F), 139.5 (large d, m, $^{1}J_{C\text{-}F}$ = 247 Hz, *C*$_{arom}$-F), 138.6 (large d, m, $^{1}J_{C\text{-}F}$ = 249 Hz, *C*$_{arom}$-F), 115.9 (t, m, $^{2}J_{C\text{-}F}$ = 17 Hz, *C*$_{arom,quaternary}$), 62.2 (s, -*C*H$_2$-), 43.8 (s, -*C*H$_2$-).

^{19}F NMR (282MHz, MeOD, 25°C, CFCl$_3$) δ -147.3 (dd, 2F), -161.8 (t, 1F, $^{3}J_{F\text{-}F}$= 20Hz), -165.5 (td, 2F)

HRMS (ESI$^+$-TOF) *m/z [M+H]$^+$* calcd for $C_9H_7F_5O_2N_2$ 271.0505, found 271.0506

Rf 0.17 (eluent : Cyclohexane/EtOAc 3/7)

Melting point 173-173.5°C

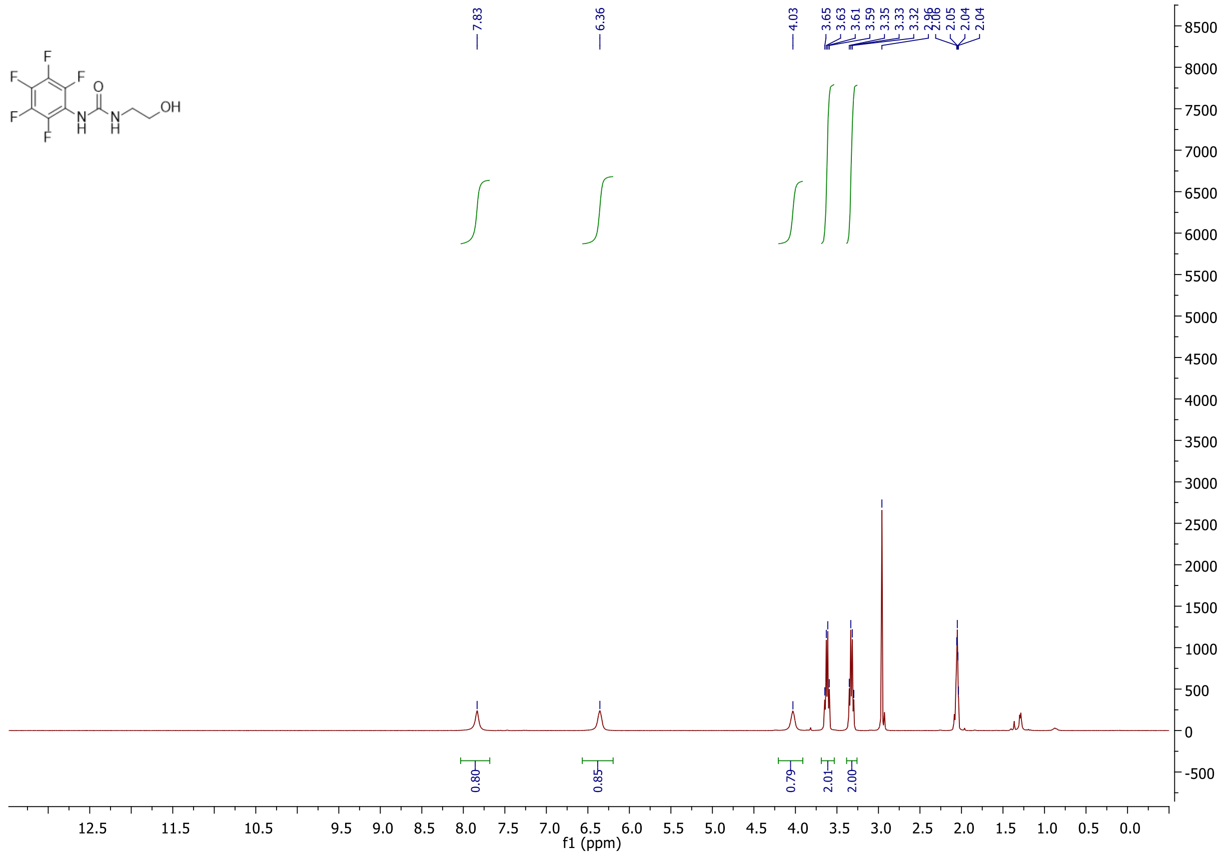

*Figure S2: ^{1}H NMR (300 MHz) spectrum of **S1** in Acetone d6*

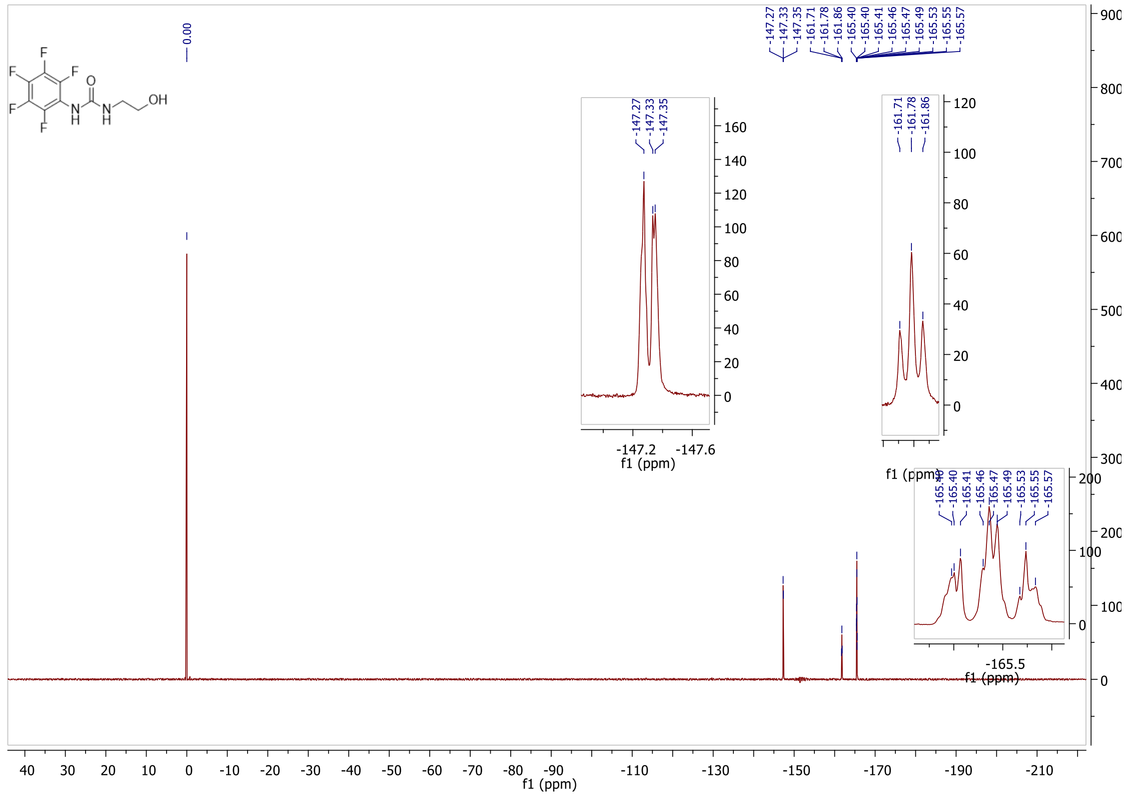

Figure S3:^{19}F NMR (282 MHz) spectrum of ***S1*** *in Acetone d6*

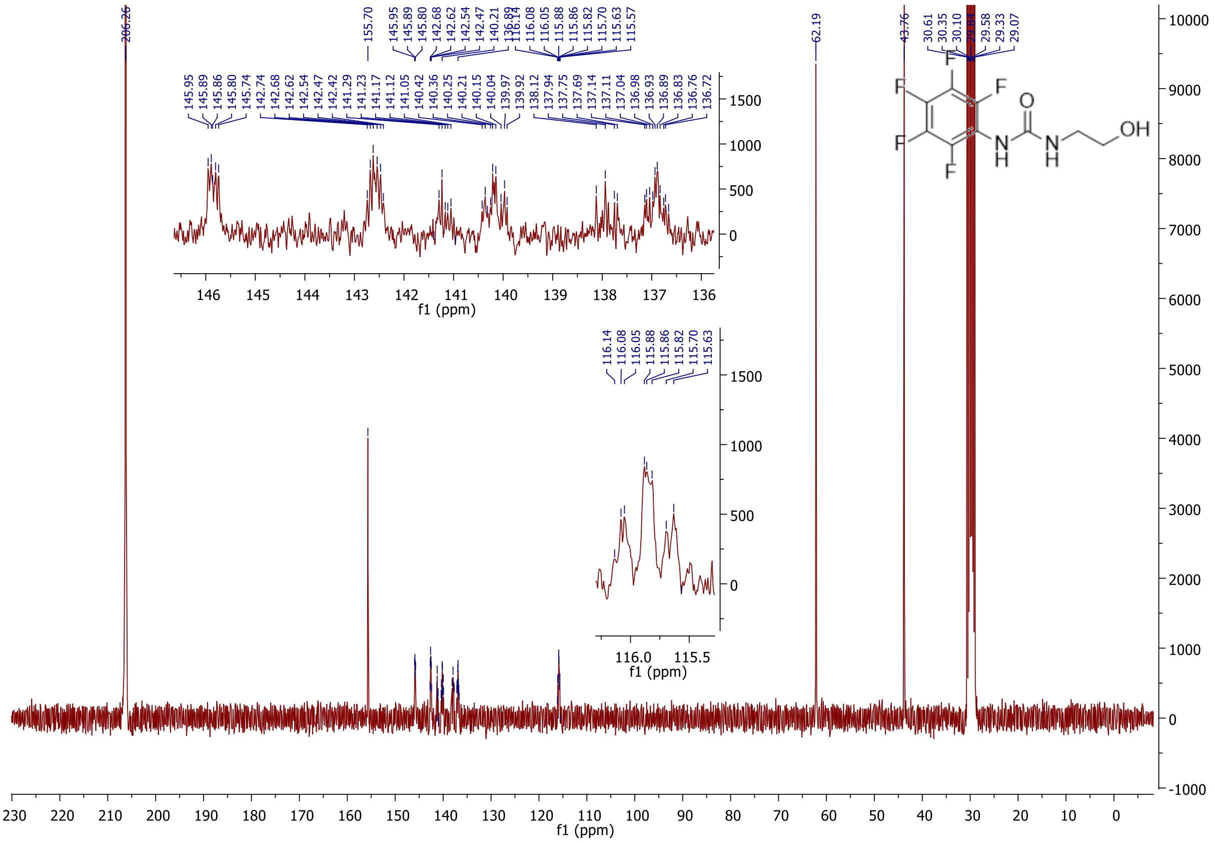

Figure S4:^{13}C NMR (75 MHz) spectrum of ***S1*** *in Acetone d6*

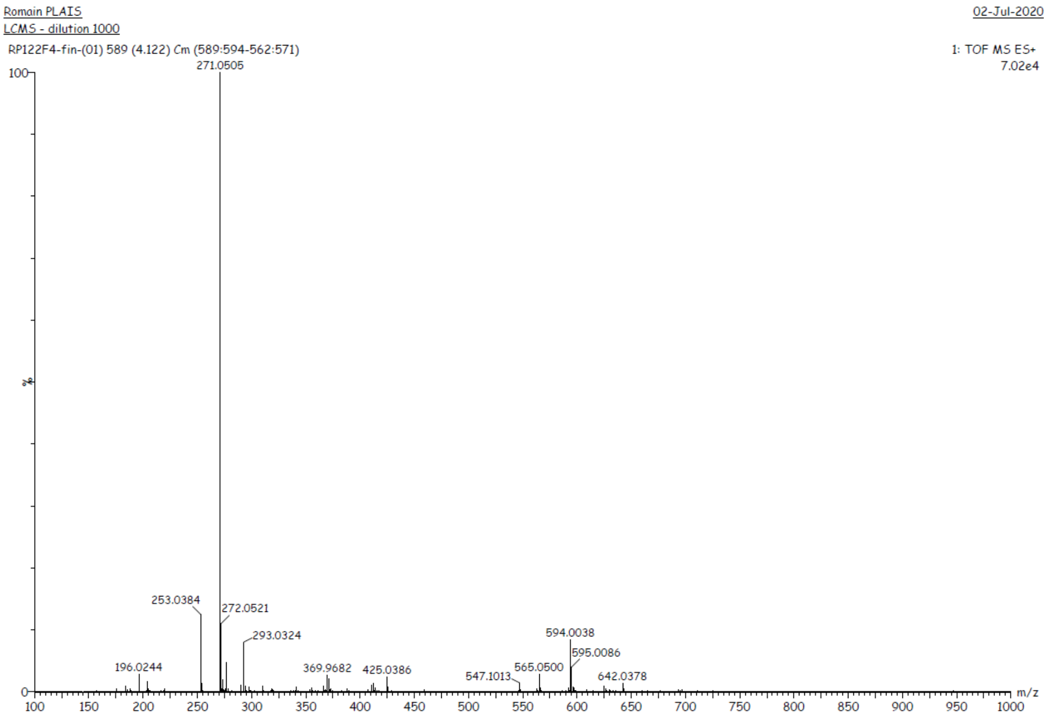

*Figure S5: Mass spectrum of **S1***

Elemental Composition Report

Page 1

Single Mass Analysis
Tolerance = 5.0 PPM / DBE: min = -1.5, max = 150.0
Element prediction: Off
Number of isotope peaks used for i-FIT = 3

Monoisotopic Mass, Even Electron Ions
1484 formula(e) evaluated with 9 results within limits (all results (up to 1000) for each mass)
Elements Used:
C: 0-100 H: 0-100 N: 0-10 O: 0-10 F: 0-6

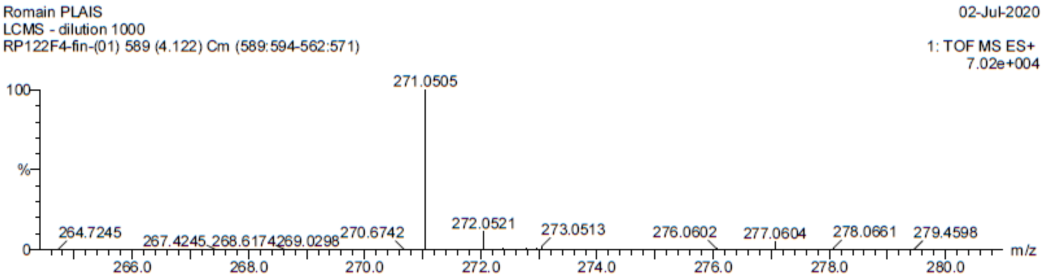

Mass	Calc. Mass	mDa	PPM	DBE	i-FIT	i-FIT (Norm)	Formula
Minimum:				-1.5			
Maximum:		5.0	5.0	150.0			
271.0505	271.0506	-0.1	-0.4	4.5	330.2	2.2	C9 H8 N2 O2 F5
	271.0504	0.1	0.4	8.5	328.3	0.3	C7 H5 N8 O2 F2
	271.0502	0.3	1.1	-0.5	332.6	4.6	C3 H10 N4 O7 F3
	271.0508	-0.3	-1.1	15.5	335.6	7.6	C17 H7 N2 O2
	271.0499	0.6	2.2	3.5	334.7	6.8	C H7 N10 O7
	271.0495	1.0	3.7	8.5	332.2	4.2	C12 H7 N2 O F4
	271.0515	-1.0	-3.7	4.5	331.2	3.3	C4 H6 N8 O3 F3
	271.0517	-1.2	-4.4	0.5	332.4	4.4	C6 H9 N2 O3 F6
	271.0492	1.3	4.8	12.5	330.5	2.6	C10 H4 N8 O F

*Figure S6: Single Mass Analysis of **S1** (TOF ES+)*

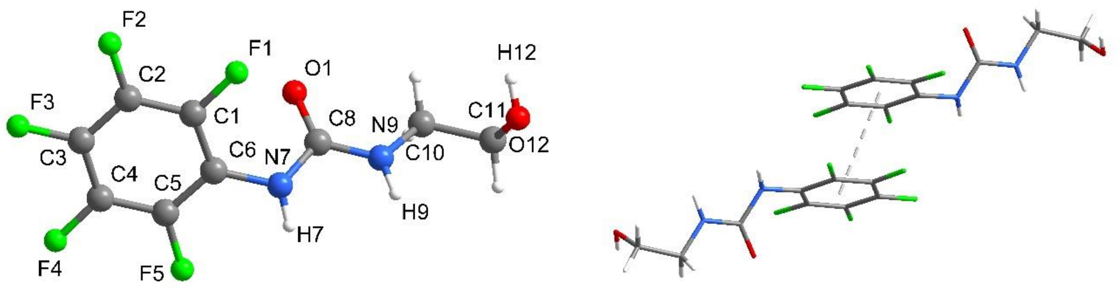

*Figure S7: X-Ray Structure and 3D arrangement in solid state of **S1***

3.4 Preparation of 1-(2-hydroxyethyl)-3-(4-nitrophenyl)urea **S2**

General procedure A was applied. 1g of 4-nitrophenylisocyanate (6.09mmol, 1.0eq), 15mL of DMF and 372mg of ethanolamine (6.09mmol, 1.0eq) were stirred at 40°C. The product was eluted by a dichloromethane/methanol (95:5) mixture to afford 900mg of desired **S2** as a yellow solid. Yield: 900mg, 66%

^{1}H NMR (300MHz, MeOD, 25°C, TMS) δ 8.16 (d, 2H, $^{3}J_{H-H}$= 9Hz, H_{arom}), 7.60 (d, 2H, $^{3}J_{H-H}$= 9Hz, H_{arom}), 3.65 (t, 2H, $^{3}J_{H-H}$= 5 Hz, -C*H*$_2$-), 3.35 (t, 2H, $^{3}J_{H-H}$= 5 Hz, -C*H*$_2$-).

^{13}C NMR (75MHz, MeOD, 25°C, TMS) δ 157.3 (s, 1C, -*C*=O), 147.8 (s, 1C, NO_2-C_{quat}), 143.1 (s, 1C, -C_{quat}), 126.0 (s, 1C, -*C*-H_{arom}), 118.5 (s, 1C, -*C*-H_{arom}), 62.0 (s, 1C, -*C*H_2-), 43.2 (s, 1C, -*C*H_2-).

HRMS (ESI$^+$-TOF) *m/z [M+H]$^+$* calcd for $C_9H_{12}N_3O_4$ 226.0828, found 226.0818.

Rf 0.6 (eluent DCM/MeOH : 95/5)

Melting point 170.5-171.5°C

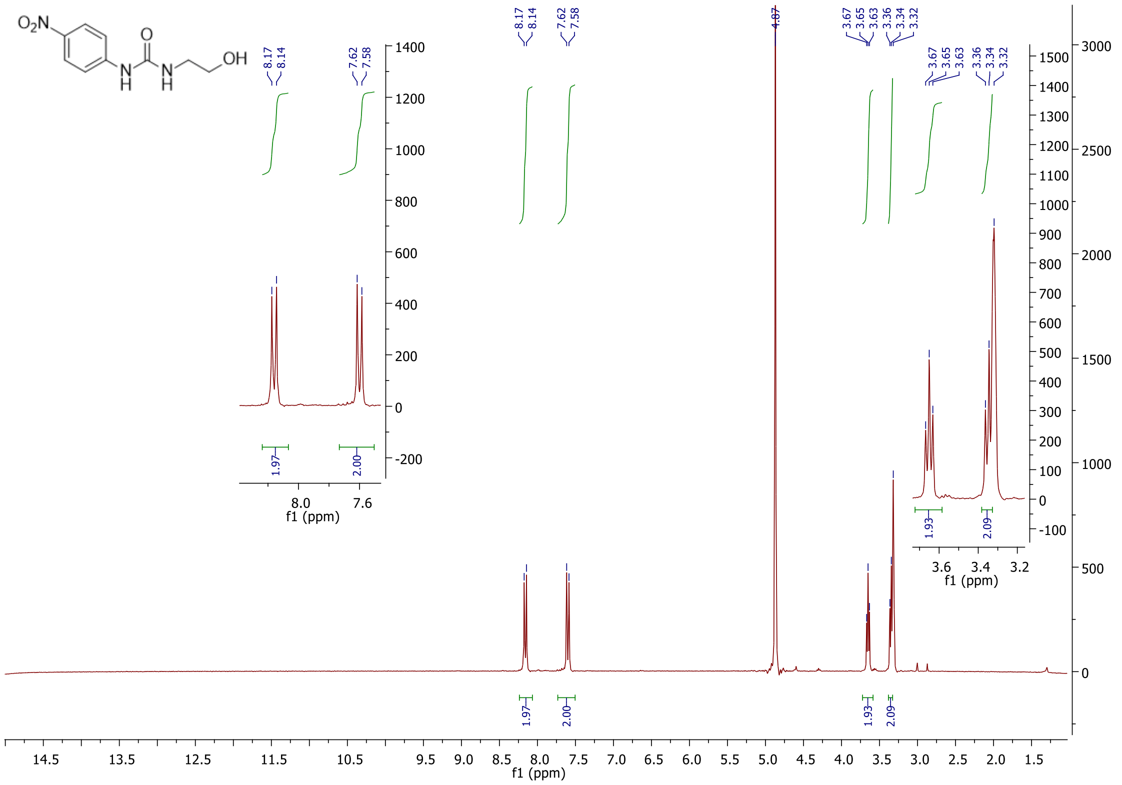

*Figure S8:^{1}H NMR (300 MHz) spectrum of **S2** in MeOD*

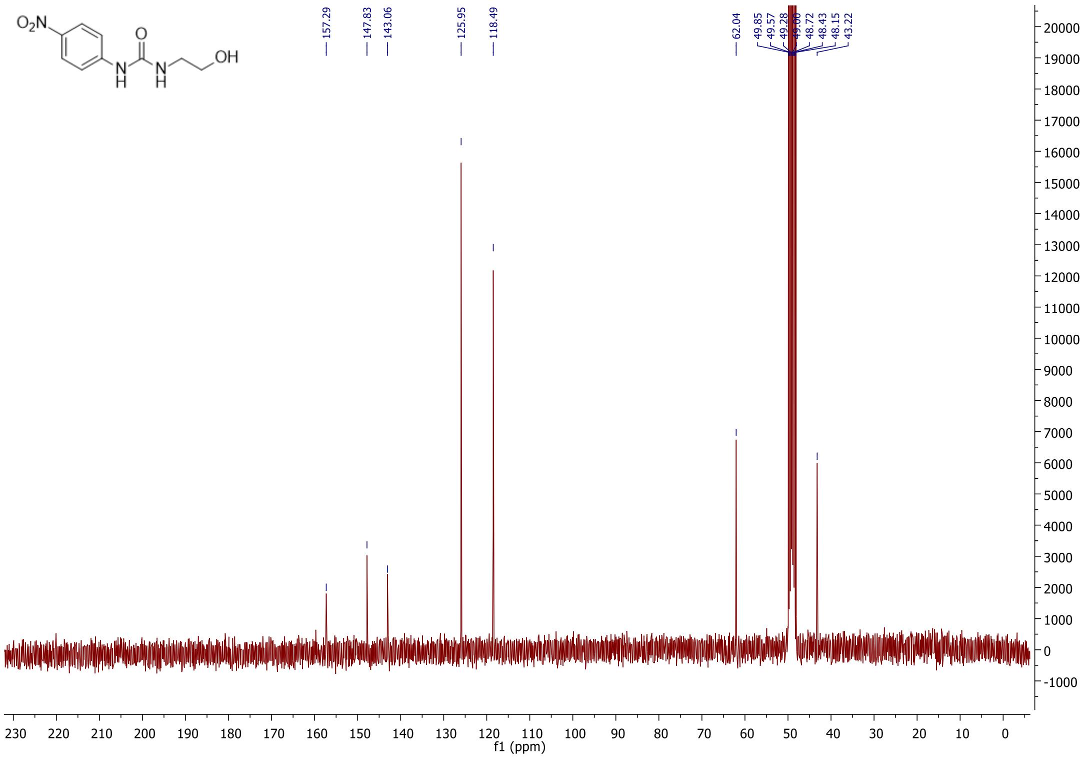

*Figure S9: ^{13}C NMR (75 MHz) spectrum of **S2** in MeOD*

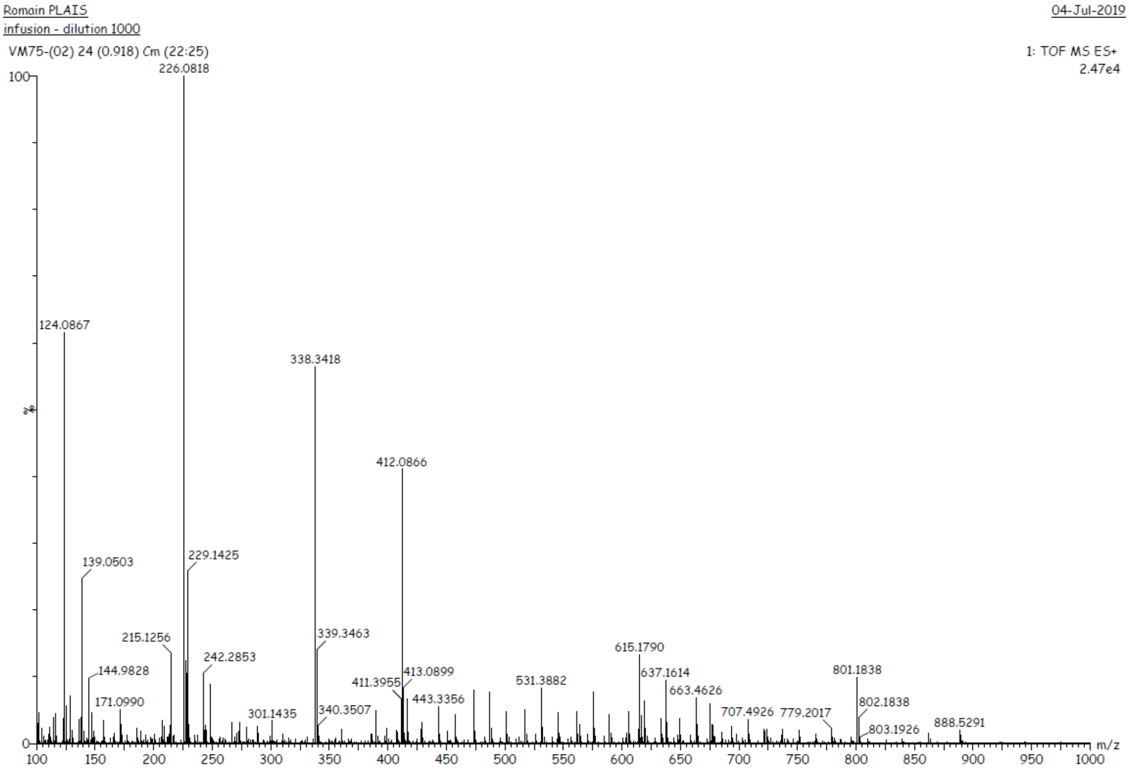

*Figure S10: Mass spectrum of **S2***

Elemental Composition Report **Page 1**

Single Mass Analysis
Tolerance = 5.0 PPM / DBE: min = -1.5, max = 150.0
Element prediction: Off
Number of isotope peaks used for i-FIT = 3

Monoisotopic Mass, Even Electron Ions
515 formula(e) evaluated with 1 results within limits (all results (up to 1000) for each mass)
Elements Used:
C: 0-110 H: 0-150 N: 0-15 O: 0-15 Si: 0-1
Romain PLAIS 04-Jul-2019
infusion - dilution 1000
VM75-(02) 24 (0.918) Cm (22:25) 1: TOF MS ES+
2.47e+004

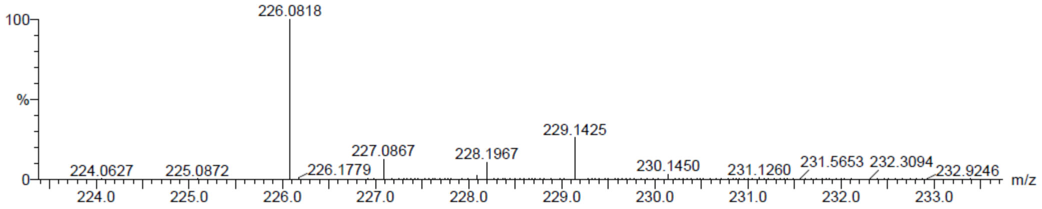

	Calc. Mass	mDa	PPM	DBE	i-FIT	i-FIT (Norm)	Formula
Minimum:				-1.5			
Maximum:		5.0	5.0	150.0			
Mass	Calc. Mass	mDa	PPM	DBE	i-FIT	i-FIT (Norm)	Formula
226.0818	226.0828	-1.0	-4.4	5.5	317.8	0.0	C9 H12 N3 O4

*Figure S11: Single Mass Analysis of **S2** (TOF ES+)*

3.5 Preparation of 1-(3,5-bis(trifluoromethyl)phenyl)-3-(3-hydroxypropyl)urea **S3**

General procedure A : 1.36mL (7.84mmol, 1.0eq) of (3,5)-bistrifluoromethylphenylisocyanate, 18mL of DMF, 623µL of 3-aminopropanol (8.15mmol, 1.04eq) were stirred at rt. The product was eluted by a

dichloromethane/methanol (95:5) mixture to afford 2g of desired 1,(3,5-bis(trifluoromethyl)phenyl)-3-(3-hydroxypropyl)urea **S3** as a white solid. Yield: 2g, 77%

^{1}H NMR (300MHz, MeOD, 25°C, TMS) δ 8.00 (s, 2H, Ar-*H*), 7.46 (s, 1H, Ar-*H*), 3.66 (t, 2H, $^{3}J_{H-H}$ = 6 Hz, -C*H2*-), 3.33 (t, 2H, $^{3}J_{H-H}$ = 7 Hz, -C*H2*-), 1.76 (quint, $^{3}J_{H-H}$ = 6 Hz, -C*H2*-).

^{13}C NMR (75MHz, MeOD, 25°C, TMS) δ 157.6 (s, 1C, *C*=O), 143.5 (s, 1C, *C*$_{arom, quat}$), 132.5-133.8 (q, 2*C*, $^{2}J_{C-F}$ = 33 Hz, *C*-CF$_3$), 119.4-133.3 (q, 2*C*, $^{1}J_{C-F}$ = 269 Hz, -*C*F$_3$), 118.9 (multiplet, 1C, C$_{arom,para}$), 115.4 (quint, 2C, $^{3}J_{C-F}$ = 4 Hz, C$_{arom}$), 60.5 (s, 1C, -*C*H$_2$-), 38.1 (s, 1C, -*C*H$_2$-), 33.7 (s, 1C, -*C*H$_2$-).

^{19}F NMR (282MHz, MeOD, 25°C) δ (not calibrated) -64.6 (s, 6F, -C*F3*).

HRMS (ESI$^+$-TOF) *m/z [M+H]$^+$* calcd for $C_{12}H_{13}F_6O_2N_2$ 331.0881, found 331.0887.

Rf 0.17 (eluent : DCM/MeOH 5%)

Melting point 106-110°C

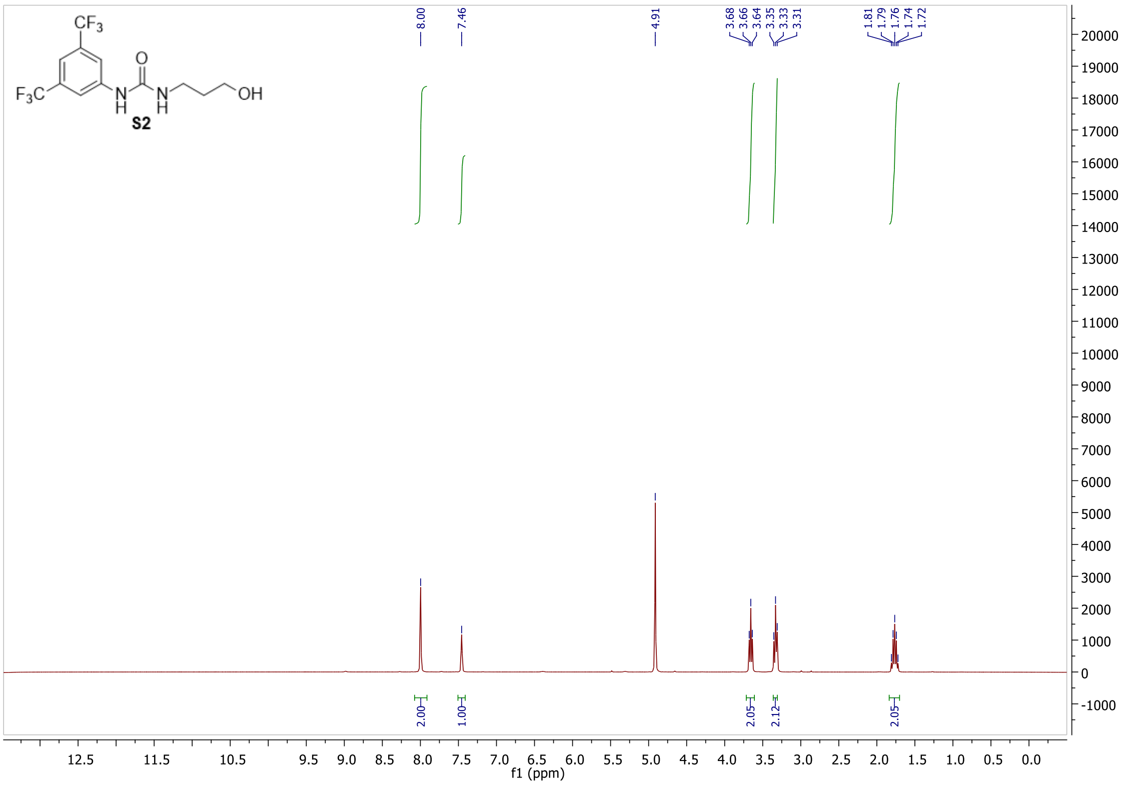

*Figure S12:^{1}H NMR (300 MHz) spectrum of **S3** in MeOD*

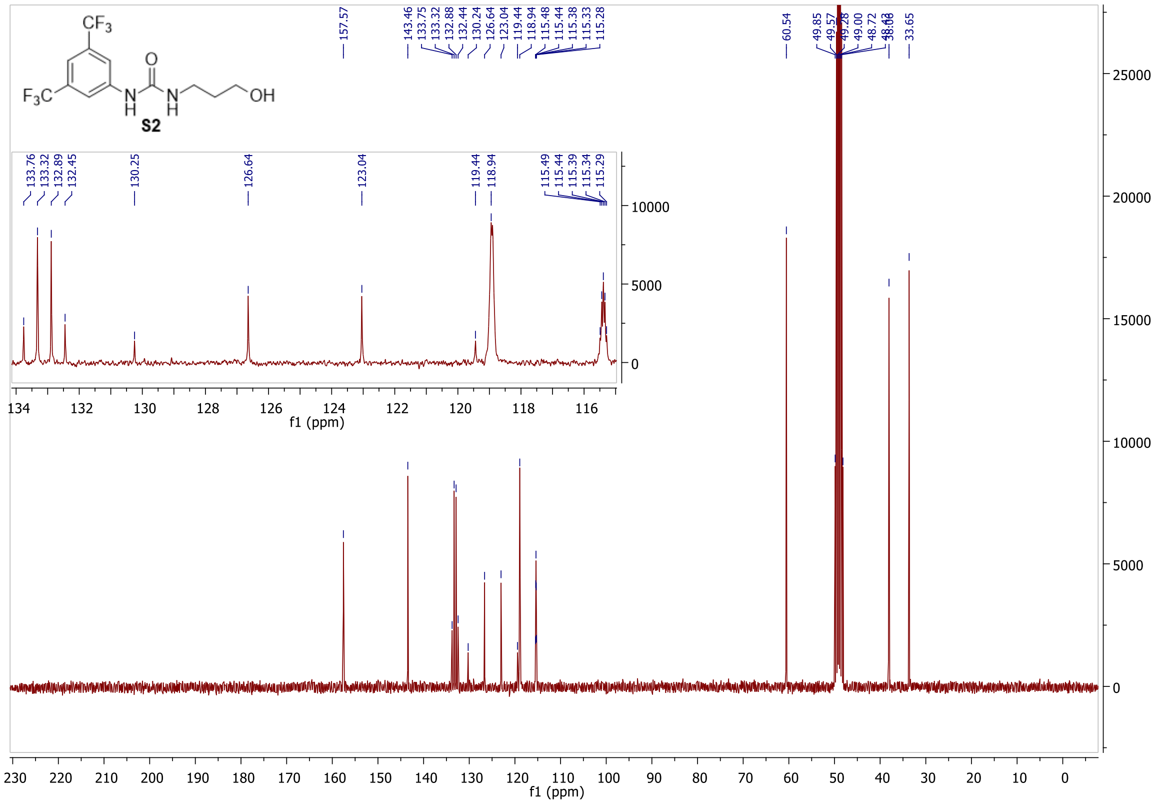

*Figure S13: ^{13}C NMR (75 MHz) spectrum of **S3** in MeOD*

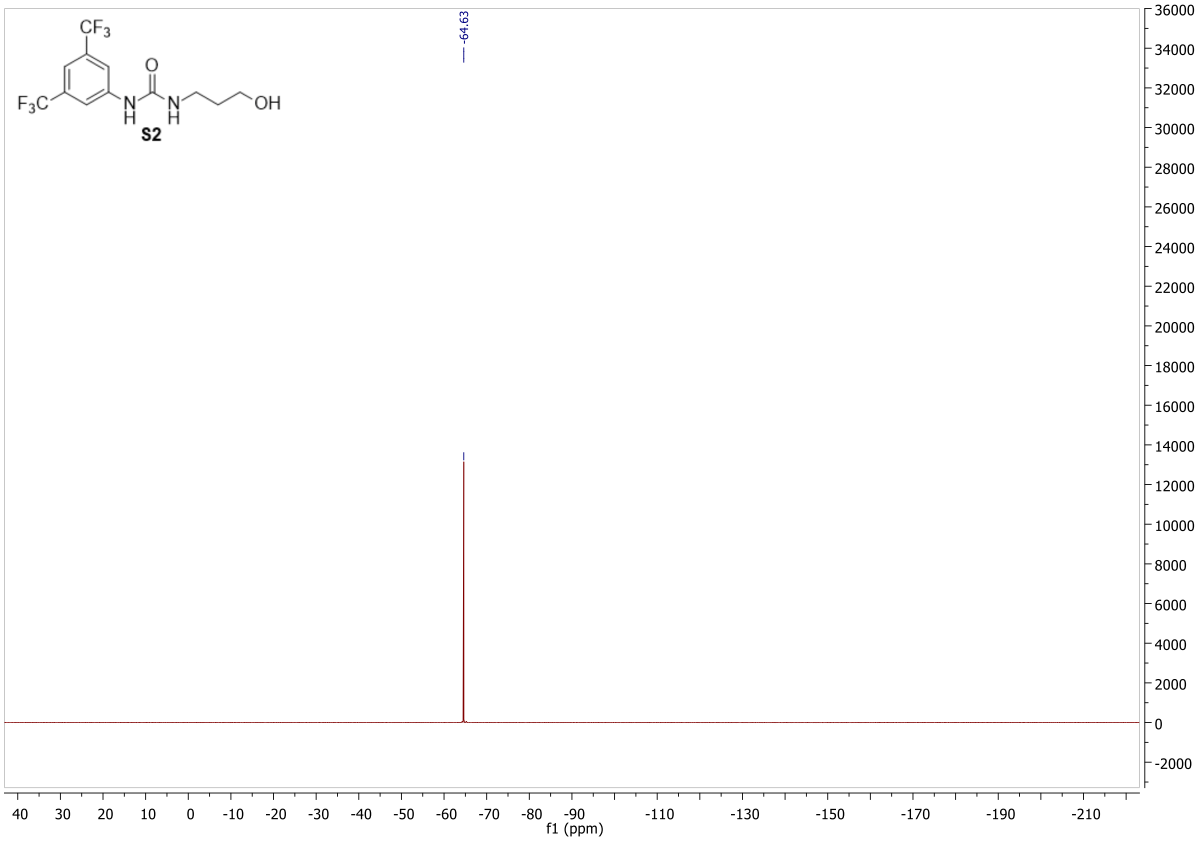

*Figure S14: ^{19}F NMR (282 MHz) spectrum of **S3** in MeOD*

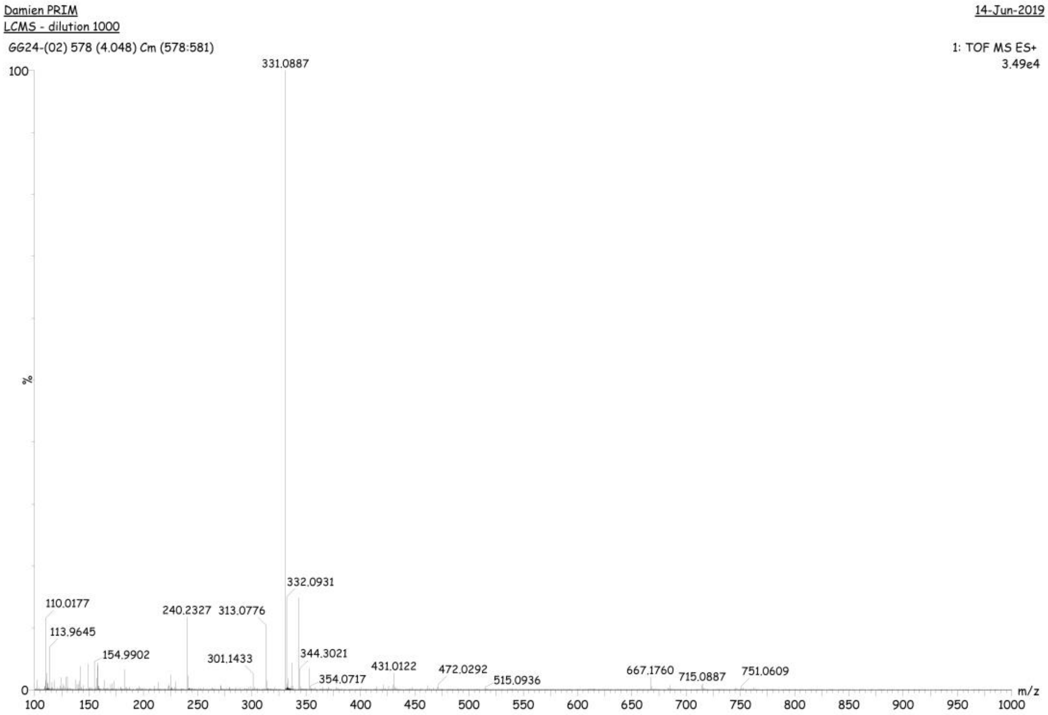

*Figure S15: Mass spectrum of **S3***

Elemental Composition Report **Page 1**

Single Mass Analysis
Tolerance = 5.0 PPM / DBE: min = -1.5, max = 150.0
Element prediction: Off
Number of isotope peaks used for i-FIT = 3

Monoisotopic Mass, Even Electron Ions
2703 formula(e) evaluated with 17 results within limits (all results (up to 1000) for each mass)
Elements Used:
C: 0-110 H: 0-150 N: 0-15 O: 0-10 F: 0-6

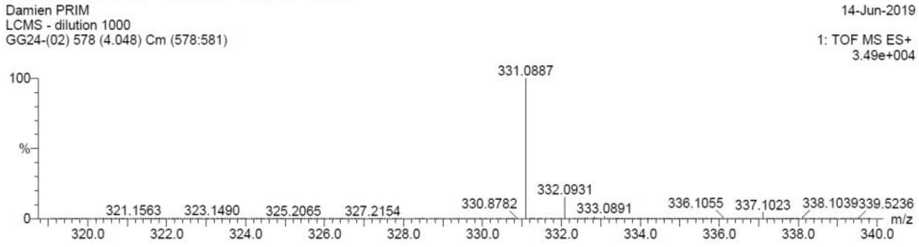

Mass	Calc. Mass	mDa	PPM	DBE	i-FIT	i-FIT (Norm)	Formula
Minimum:				-1.5			
Maximum:		5.0	5.0	150.0			
331.0887	331.0881	0.6	1.8	4.5	279.5	1.0	C12 H13 N2 O2 F6
	331.0879	0.8	2.4	8.5	279.8	1.2	C10 H10 N8 O2 F3
	331.0876	1.1	3.3	12.5	280.4	1.9	C8 H7 N14 O2
	331.0903	-1.6	-4.8	11.5	280.6	2.1	C12 H11 N8 O4
	331.0890	-0.3	-0.9	6.5	281.4	2.9	C11 H15 N4 O8
	331.0901	-1.4	-4.2	2.5	284.0	5.5	C8 H16 N4 O9 F
	331.0890	-0.3	-0.9	4.5	284.3	5.7	C7 H11 N8 O3 F4
	331.0894	-0.7	-2.1	11.5	284.6	6.1	C17 H13 N2 O3 F2
	331.0888	-0.1	-0.3	8.5	285.2	6.7	C5 H8 N14 O3 F
	331.0877	1.0	3.0	-0.5	285.7	7.2	C6 H15 N4 O7 F4
	331.0883	0.4	1.2	15.5	286.7	8.1	C20 H12 N2 O2 F
	331.0874	1.3	3.9	3.5	287.1	8.6	C4 H12 N10 O7 F
	331.0902	-1.5	-4.5	0.5	287.2	8.7	C4 H12 N8 O4 F5
	331.0871	1.6	4.8	19.5	288.2	9.7	C23 H11 N2 O
	331.0899	-1.2	-3.6	4.5	288.4	9.9	C2 H9 N14 O4 F2
	331.0886	0.1	0.3	-0.5	289.8	11.3	C H13 N10 O8 F2
	331.0875	1.2	3.6	1.5	291.0	12.5	H8 N14 O2 F5

*Figure S16: Single Mass Analysis of **S3** (TOF ES+)*

3.6 Preparation of 1-(2-((6-chloro-1,2,4,5-tetrazin-3-yl)oxy)ethyl)-3-(perfluorophenyl)urea **1**

General procedure B : 100mg of **S1** (0.37mmol), 56mg of dichlorotetrazine (0.37mmol), 12mL of DCM and 51µL of 2,4,6-collidine (0.39mmol). The mixture was stirred at rt for a night and purified by flash chromatography, eluting with a mixture of Cyclohexane/EtOAc 8/2 to afford 32mg of **1** as a pink fluorescent solid. Yield: 23%.

^{1}H NMR (300MHz, Acetone d6, 25°C, TMS) δ 7.86 (broad s, 1H, -N-*H*), 6.66 (broad s, 1H, -N-*H*), 4.77 (t, 2H, $^{3}J_{H-H}$=6Hz), 3.79 (q, 2H, $^{3}J_{H-H}$=6Hz).

^{13}C NMR (75MHz, Acetone d6, 25°C, TMS) δ 167.9 (s, 1C, $C_{Tetrazine}$), 164.8 (s, 1C, $C_{Tetrazine}$), 155.4 (s, 1C, *C*=O), 144.2 (large d, m, $^{1}J_{C-F}$ = 173 Hz, C_{arom}-F), 139.7 (large d, m, $^{1}J_{C-F}$ = 248 Hz, C_{arom}-F), 138.5 (large d, m, $^{1}J_{C-F}$ = 248 Hz, C_{arom}-F), 115.4 (t, m, $^{2}J_{C-F}$ = 14 Hz, C_{arom}-F), 70.3 (s, -CH_2-), 39.7 (s, -CH_2-).

^{19}F NMR (282MHz, MeOD, 25°C) (not calibrated) δ 29.4-29.3 (m, 2F), 15.1 (t, 1F, $^{2}J_{C-F}$ = 23 Hz), 11.2-11.0 (m, 2F).

UV-Visible (Acetonitrile) λ_{max} (ε)= 220 nm (3.67 AU), 265 nm (0.25 AU), 324 nm (0.47 AU), 511 nm (0.11 AU).

Fluorescence (Acetonitrile) λ_{exc}= 511 nm, $\lambda_{em,max}$= 563 nm

HRMS (ESI^{+}-TOF) *m/z [M+H]$^{+}$* calcd for $C_{11}H_7N_6O_2F_5Cl$ 385.0239, found 385.0232

Rf 0.57 (eluent: Cyclohexane/EtOAc 1:1)

Melting point 171.5-172.5°C

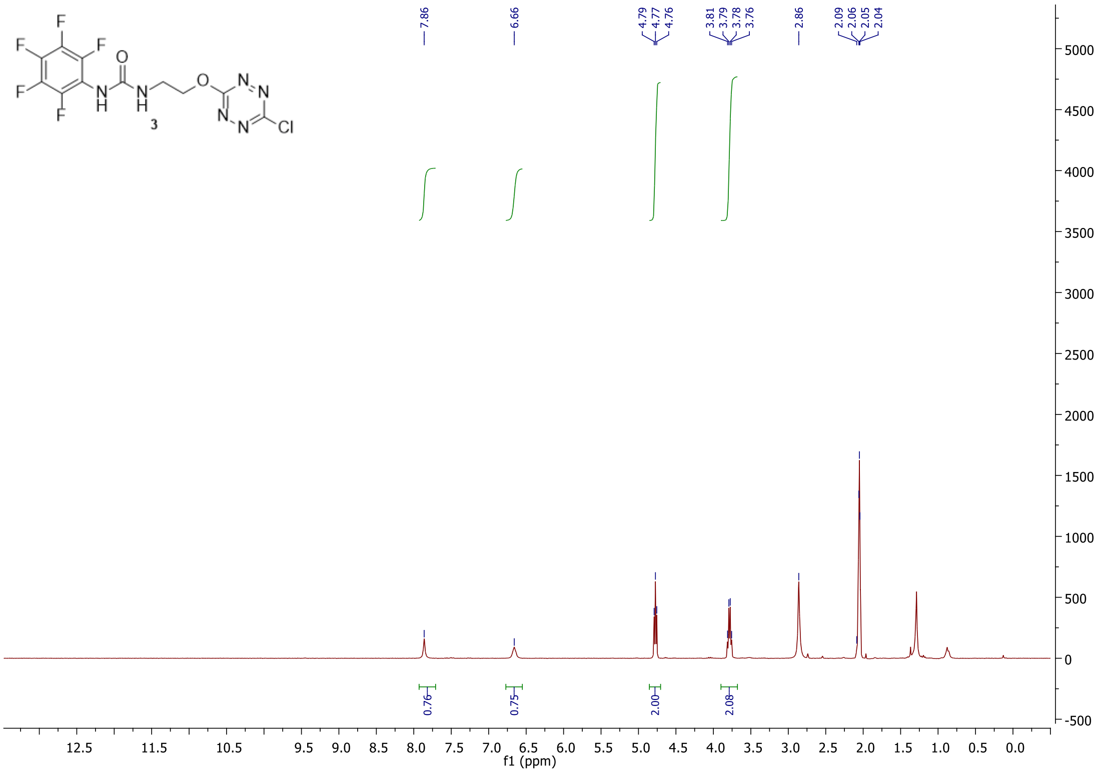

*Figure S17:^{1}H NMR (300 MHz) spectrum of **1** in Acetone d6*

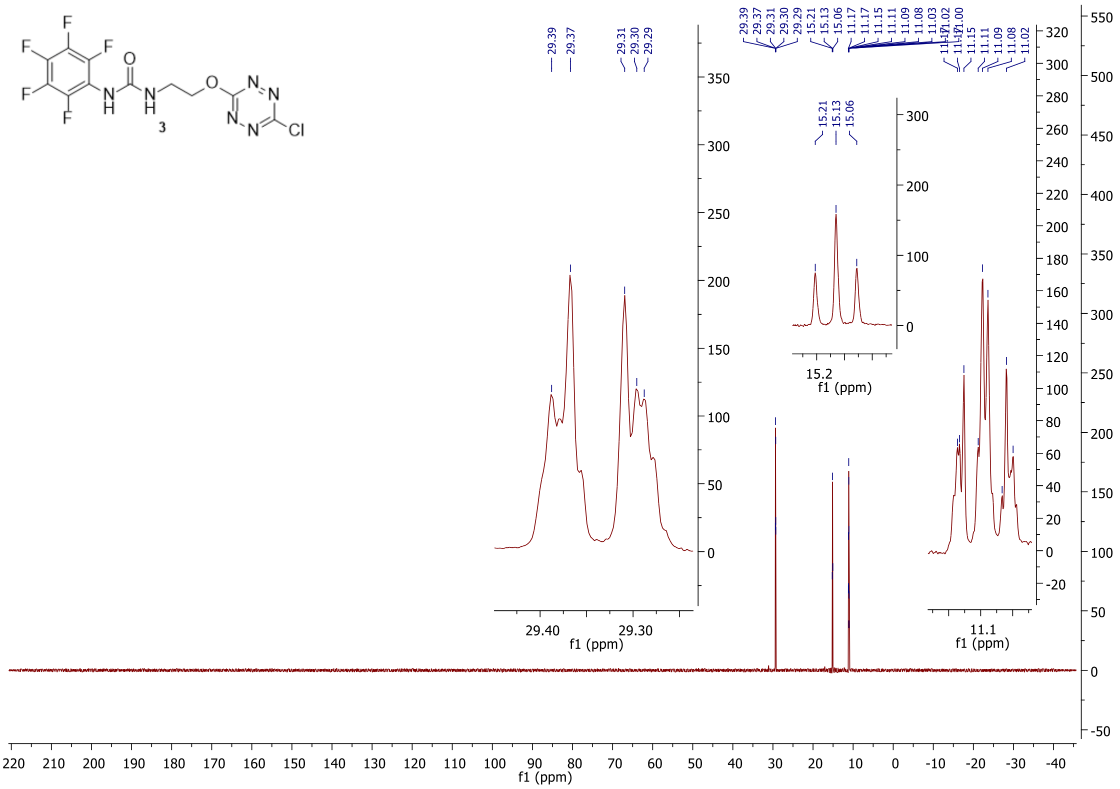

*Figure S18:^{19}F NMR (282 MHz) spectrum of **1** in Acetone d6*

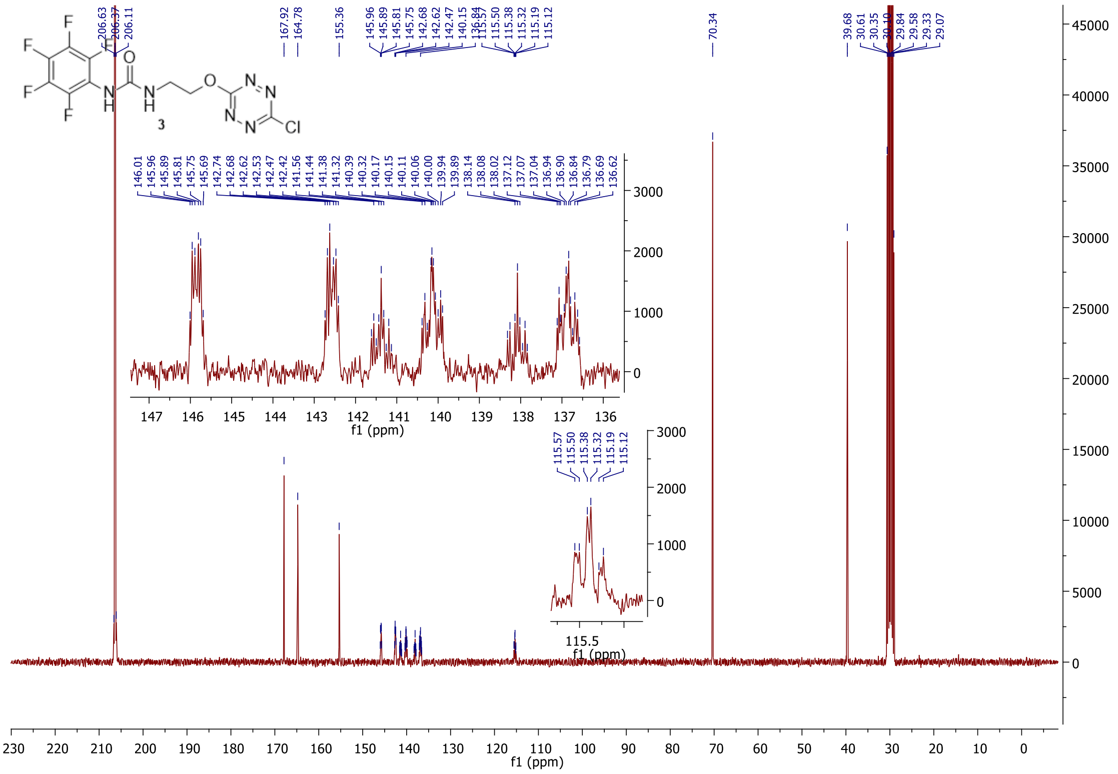

*Figure S19:^{13}C NMR (75 MHz) spectrum of **1** in Acetone d6*

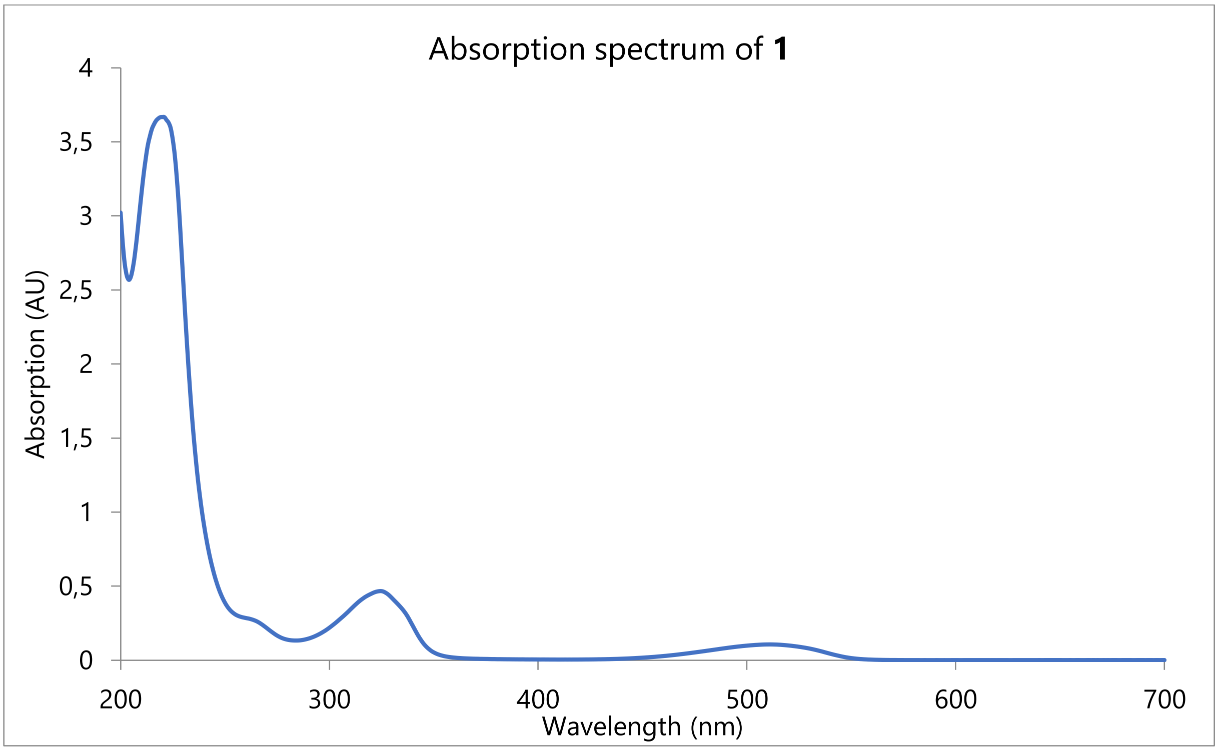

*Figure S20: Absorption spectrum of **1** in Acetonitrile*

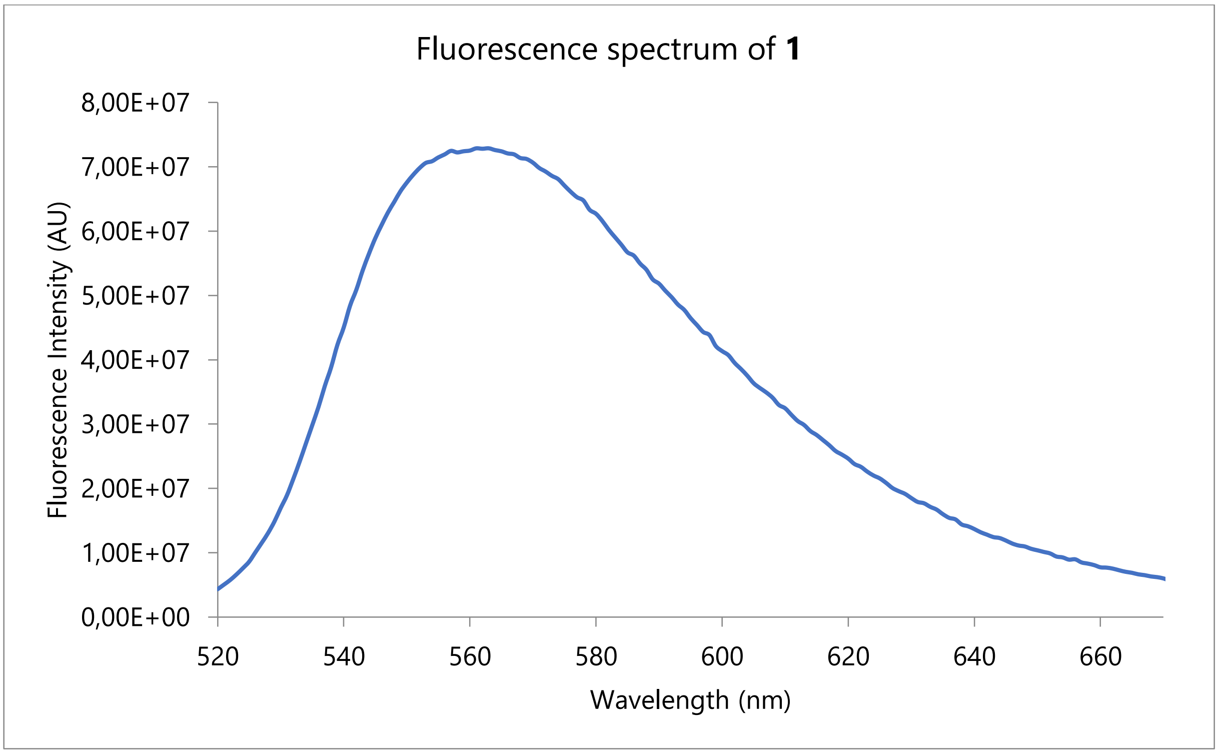

*Figure S21: Fluorescence spectrum of **1** in Acetonitrile*

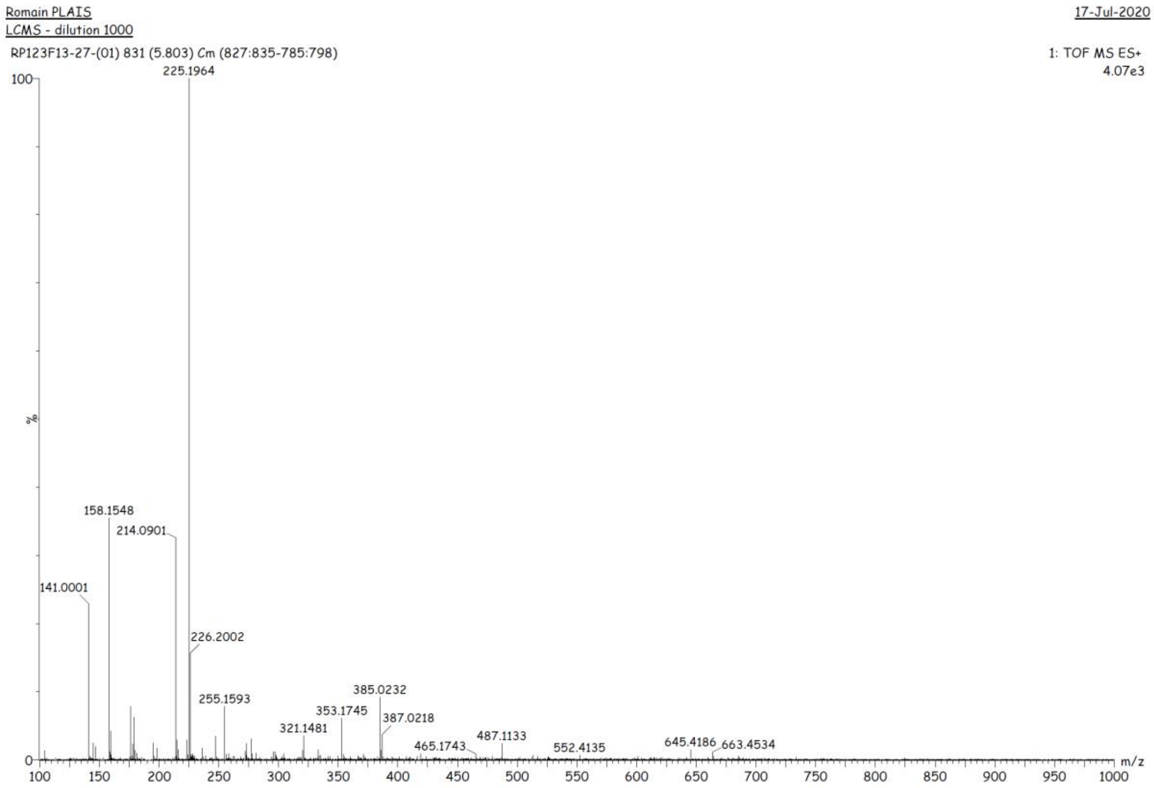

Figure S22: Mass spectrum of **1**

Elemental Composition Report

Page 1

Single Mass Analysis

Tolerance = 5.0 PPM / DBE: min = -1.5, max = 150.0
Element prediction: Off
Number of isotope peaks used for i-FIT = 3

Monoisotopic Mass, Even Electron Ions
5525 formula(e) evaluated with 39 results within limits (all results (up to 1000) for each mass)
Elements Used:
C: 0-100 H: 0-100 N: 0-10 O: 0-10 F: 0-7 Cl: 0-1

Romain PLAIS 17-Jul-2020
LCMS - dilution 1000
RP123F13-27-(01) 831 (5.803) Cm (827:835-785:798) 1: TOF MS ES+
3.74e+002

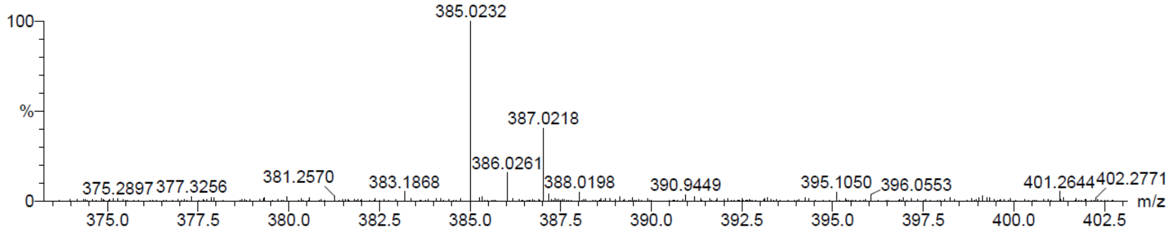

Mass	Calc. Mass	mDa	PPM	DBE	i-FIT	i-FIT (Norm)	Formula
Minimum:				-1.5			
Maximum:		5.0	5.0	150.0			
385.0232	385.0239	-0.7	-1.8	8.5	51.4	1.8	C11 H7 N6 O2 F5 Cl
	385.0239	-0.7	-1.8	10.5	51.5	1.9	C15 H11 N2 O7 F Cl
	385.0230	0.2	0.5	8.5	51.9	2.3	C16 H9 O F7 Cl
	385.0250	-1.8	-4.7	6.5	51.9	2.3	C12 H12 N2 O8 F2 Cl
	385.0226	0.6	1.6	3.5	52.0	2.4	C10 H11 N2 O6 F5 Cl
	385.0228	0.4	1.0	12.5	52.0	2.4	C14 H6 N6 O F4 Cl
	385.0223	0.9	2.3	7.5	52.3	2.7	C8 H8 N8 O6 F2 Cl
	385.0214	1.8	4.7	7.5	52.6	2.9	C13 H10 N2 O5 F4 Cl
	385.0227	0.5	1.3	14.5	52.8	3.2	C18 H10 N2 O6 Cl
	385.0251	-1.9	-4.9	4.5	53.0	3.4	C8 H8 N6 O3 F6 Cl
	385.0237	-0.5	-1.3	-0.5	53.2	3.6	C7 H12 N2 O7 F6 Cl
	385.0235	-0.3	-0.8	3.5	53.3	3.7	C5 H9 N8 O7 F3 Cl
	385.0219	1.3	3.4	12.5	53.9	4.3	C19 H8 F6 Cl
	385.0241	-0.9	-2.3	19.5	53.9	4.3	C19 H6 N6 O2 Cl
	385.0243	-1.1	-2.9	15.5	54.2	4.5	C21 H9 O2 F3 Cl
	385.0216	1.6	4.2	16.5	54.2	4.6	C17 H5 N6 F3 Cl
	385.0232	0.0	0.0	19.5	54.8	5.2	C24 H8 O F2 Cl
	385.0246	-1.4	-3.6	-0.5	55.2	5.6	C2 H10 N8 O8 F4 Cl
	385.0220	1.2	3.1	23.5	56.1	6.5	C27 H7 F Cl
	385.0217	1.5	3.9	8.5	60.6	11.0	C6 H4 N10 O7 F3
	385.0221	1.1	2.9	13.5	60.8	11.2	C12 H2 N8 O2 F5
	385.0219	1.3	3.4	4.5	60.9	11.3	C8 H7 N4 O7 F6
	385.0232	0.0	0.0	9.5	61.0	11.4	C9 H3 N8 O3 F6
	385.0228	0.4	1.0	4.5	61.1	11.5	C3 H5 N10 O8 F4
	385.0221	1.1	2.9	15.5	61.2	11.5	C16 H6 N4 O7 F
	385.0230	0.2	0.5	0.5	61.3	11.7	C5 H8 N4 O8 F7
	385.0241	-0.9	-2.3	11.5	61.4	11.8	C8 H5 N10 O9
	385.0232	0.0	0.0	11.5	61.4	11.8	C13 H7 N4 O8 F2
	385.0222	1.0	2.6	24.5	61.4	11.8	C20 H N8 O2
	385.0234	-0.2	-0.5	20.5	61.6	12.0	C17 H2 N8 O3 F
	385.0244	-1.2	-3.1	5.5	61.7	12.1	C6 H4 N8 O4 F7
	385.0243	-1.1	-2.9	7.5	61.9	12.3	C10 H8 N4 O9 F3
	385.0245	-1.3	-3.4	16.5	62.1	12.5	C14 H3 N8 O4 F2
	385.0225	0.7	1.8	20.5	62.1	12.5	C22 H4 N2 O2 F3
	385.0213	1.9	4.9	24.5	62.1	12.5	C25 H3 N2 O F2
	385.0239	-0.7	-1.8	0.5	62.2	12.6	H6 N10 O9 F5
	385.0236	-0.4	-1.0	16.5	62.3	12.7	C19 H5 N2 O3 F4
	385.0248	-1.6	-4.2	12.5	62.7	13.1	C16 H6 N2 O4 F5
	385.0249	-1.7	-4.4	23.5	63.2	13.6	C24 H5 N2 O4

*Figure S23: Single Mass Analysis of **1** (TOF ES+)*

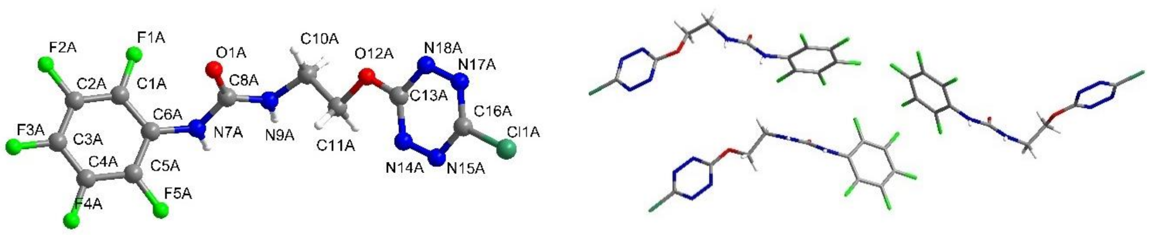

*Figure S24: X-Ray Structure and 3D arrangement in solid state of **1***

3.7 Preparation of 1-(2-((6-chloro-1,2,4,5-tetrazin-3-yl)oxy)ethyl)-3-(4-nitrophenyl)urea **2**

General procedure B : 100mg of **S2** (0.44mmol), 66mg of dichlorotetrazine (0.44mmol), 15mL of DCM and 56µL of 2,4,6-collidine (0.46mmol). The mixture was stirred at 40°C for a night and purified by flash chromatography, eluting with a gradient of Cyclohexane/EtOAc 6/4 to 4/6 to afford 50mg of **2** as a pink fluorescent solid. Yield: 33%.

^{1}H NMR (300MHz, Acetone d6, 25°C, TMS) δ 8.76 (broad s, 1H, N-*H*), 8.15 (d, 2H, $^{3}J_{H-H}$= 9 Hz, -C-H_{arom}), 7.72 (d, 2H, $^{3}J_{H-H}$= 9 Hz, -C-H_{arom}), 6.48 (broad s, 1H, -N-*H*), 4.80 (t, 2H, $^{3}J_{H-H}$= 5 Hz, -CH_2-), 3.81 (q, 2H, $^{3}J_{H-H}$= 5 Hz, -CH_2-).

^{13}C NMR (75MHz, Acetone d6, 25°C, TMS) δ 168.0 (s, 1C, $C_{Tétrazine}$), 164.8 (s, 1C, $C_{Tétrazine}$), 155.5 (s, 1C, *C*=O), 147.8 (s, 1C, C_{arom}-NO_2), 142.4 (s, 1C, NH-C_{arom}), 125.7 (s, 1C, C_{arom}), 118.1 (s, 1C, C_{arom}), 70.4 (s, 1C, -*C*H_2-), 39.4 (s, 1C, -*C*H_2-).

UV-Visible (Acetonitrile) λ_{max} (ε)= 510 nm (0.12 A.U.), 329 nm (3.70 A.U.), 219 nm (4.17 A.U.).

Fluorescence (Acetonitrile) λ_{exc}= 510 nm, $\lambda_{em,max}$= 561 nm.

HRMS (ESI^{+}-TOF) *m/z [M+NO_3]$^{-}$* calcd for $C_{11}H_{10}ClN_8O_7^{-}$ 401.0361, found 401.0358

Rf 0.26 (eluent: Cyclohexane/EtOAc 6/4)

Melting point 149.5-150°C

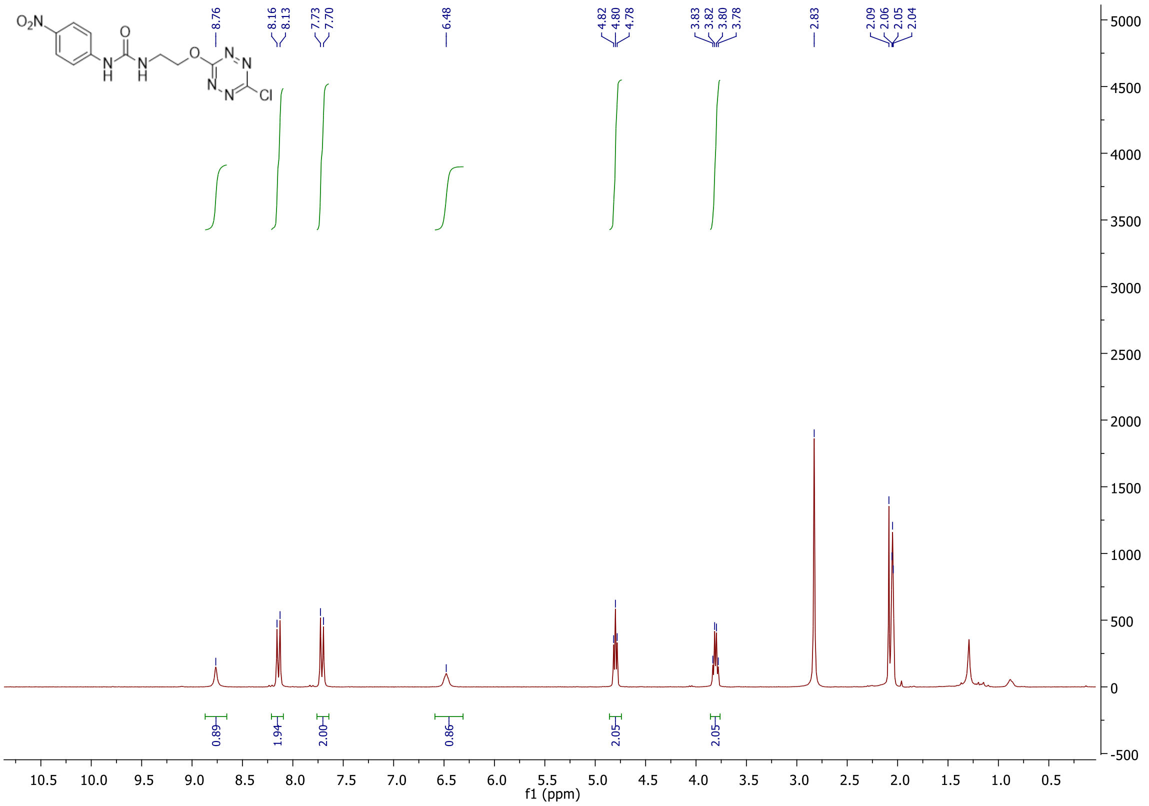

*Figure S25:^{1}H NMR (300 MHz) spectrum of **2** in Acetone d6*

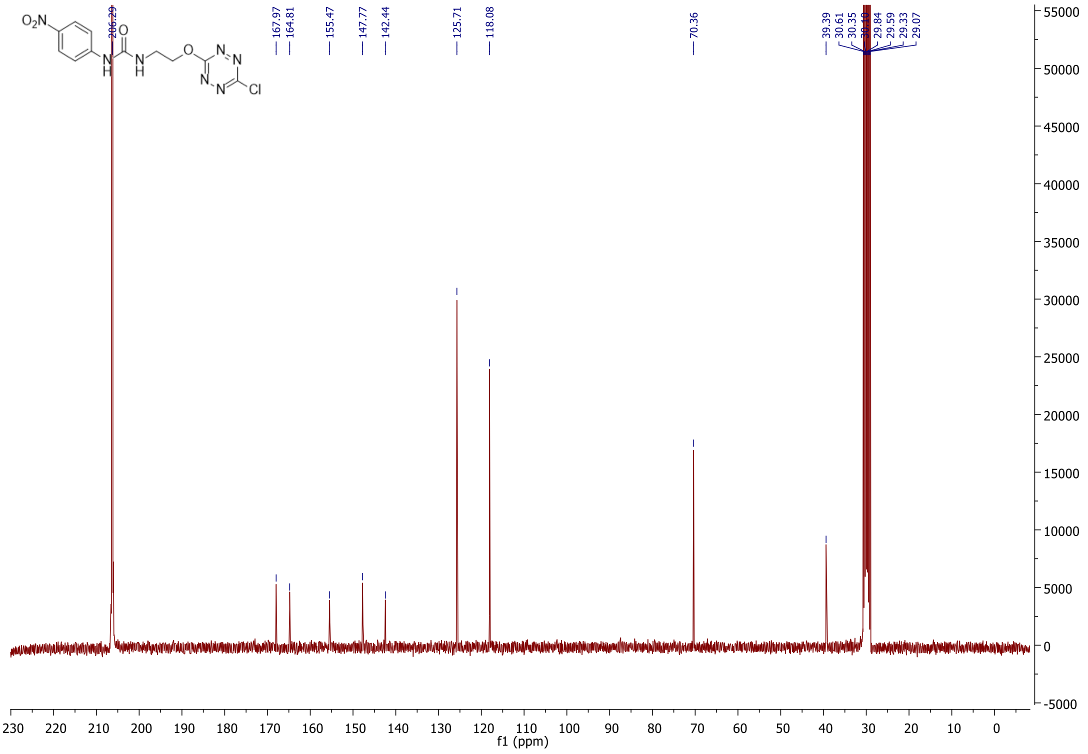

*Figure S26:^{13}C NMR (75 MHz) spectrum of **2** in Acetone d6*

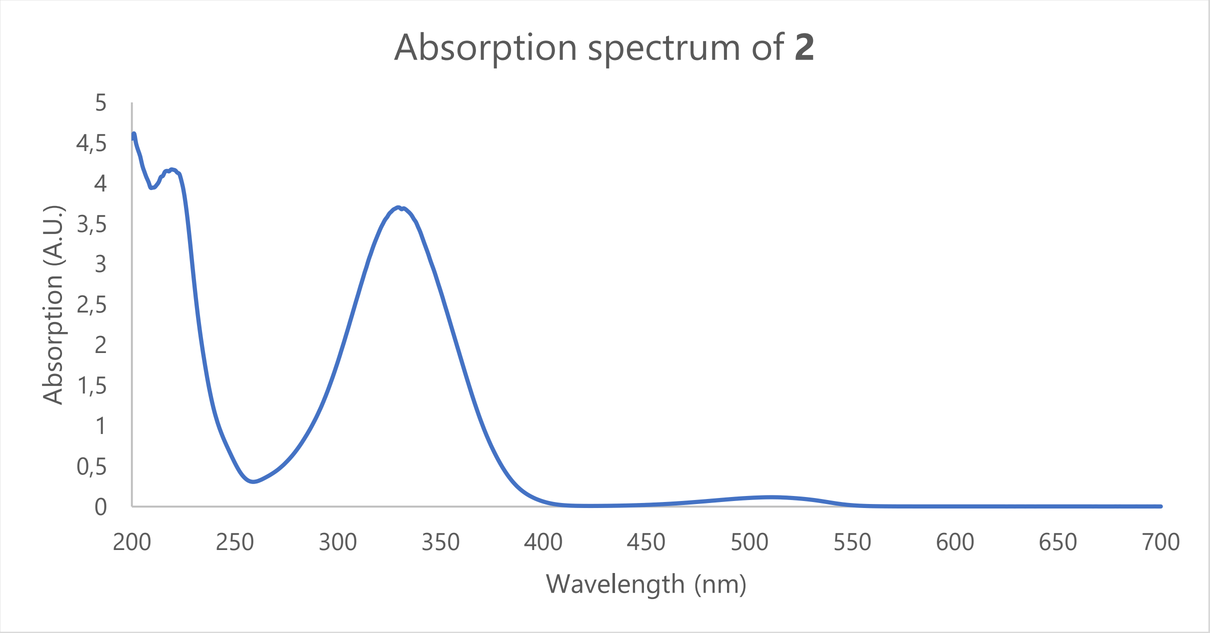

*Figure S27: Absorption spectrum of **2** in Acetonitrile*

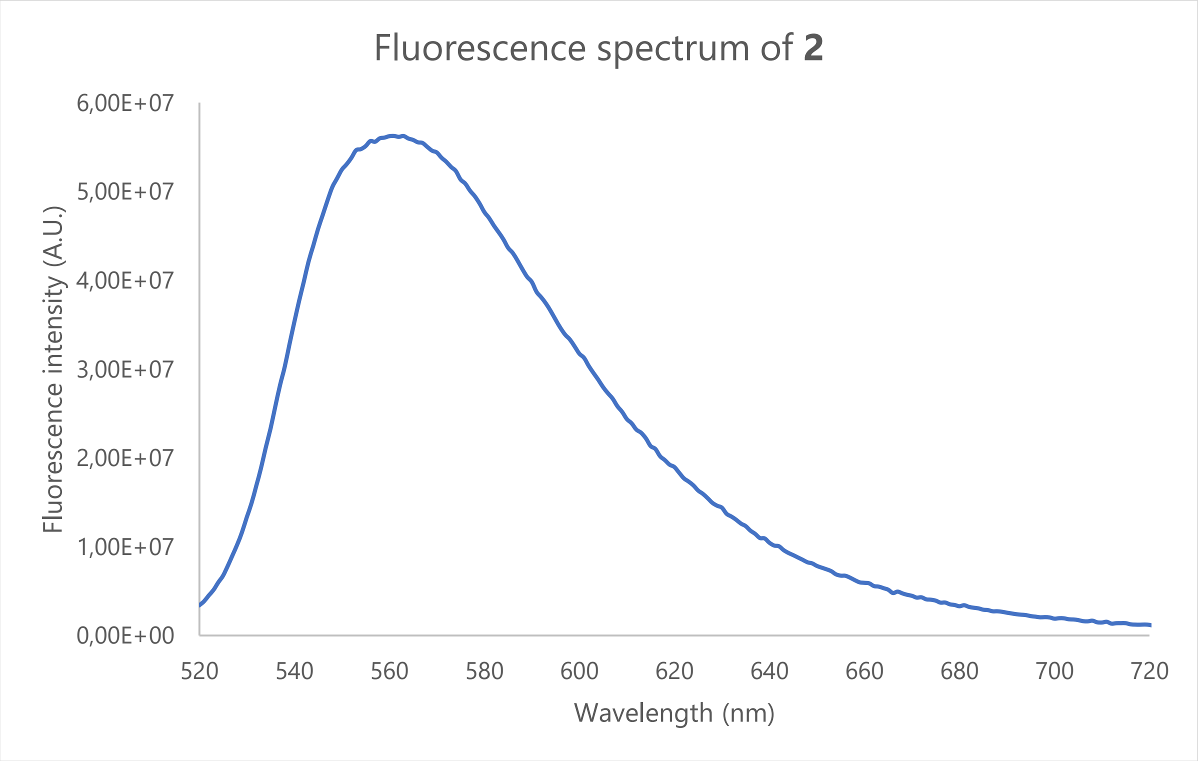

*Figure S28: Fluorescence spectrum of **2** in Acetonitrile*

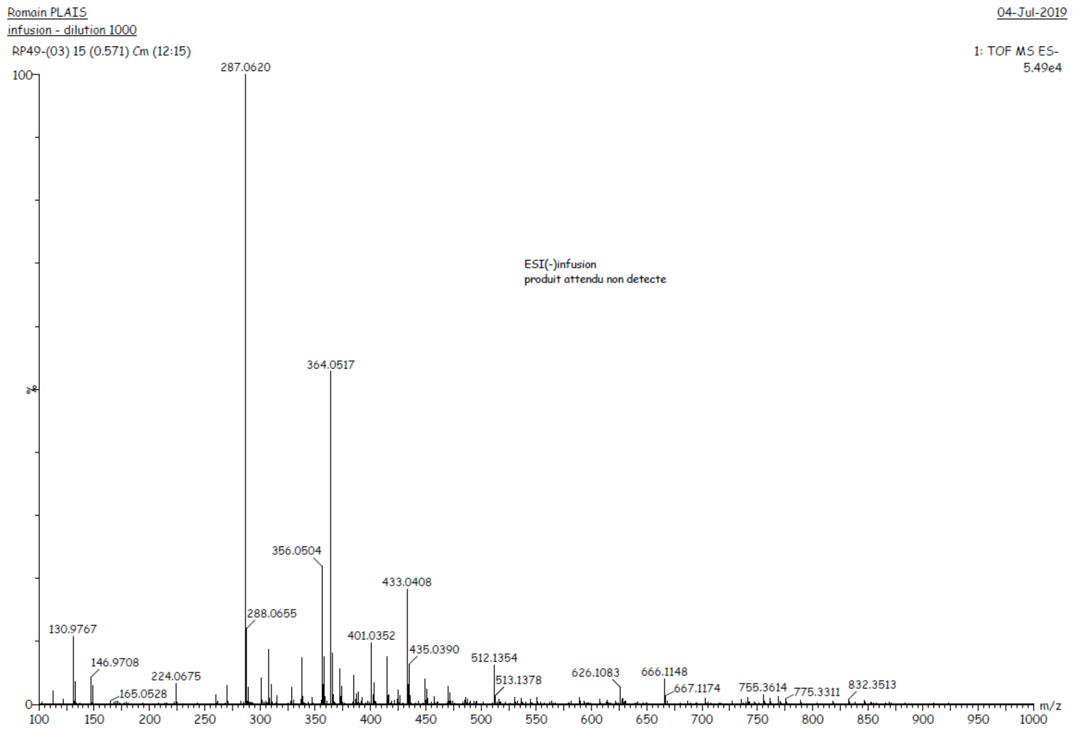

*Figure S29: Mass spectrum of **2** (observed as **2**-NO_3^-) (ESI-)*

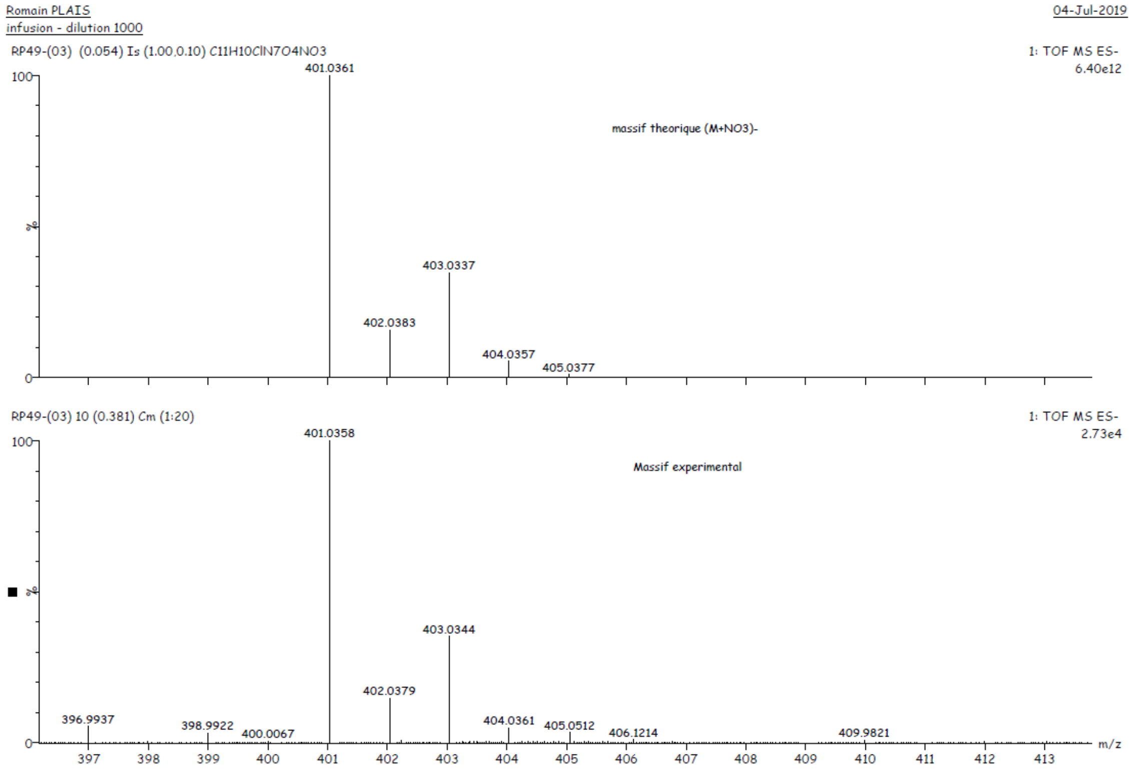

*Figure S30: Theoretical isotopic distribution vs. experimental isotopic distribution of **2**-NO_3^- (ESI-)*

Elemental Composition Report **Page 1**

Single Mass Analysis
Tolerance = 5.0 PPM / DBE: min = -1.5, max = 150.0
Element prediction: Off
Number of isotope peaks used for i-FIT = 3

Monoisotopic Mass, Even Electron Ions
1575 formula(e) evaluated with 9 results within limits (all results (up to 1000) for each mass)
Elements Used:
C: 0-100 H: 0-150 N: 0-10 O: 0-10 Cl: 0-1 I: 0-1

Romain PLAIS 04-Jul-2019
infusion - dilution 1000
RP49-(03) 10 (0.381) Cm (1:20) 1: TOF MS ES-
2.73e+004

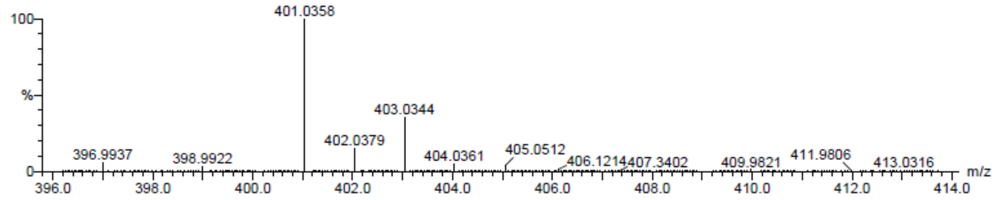

Mass	Calc. Mass	mDa	PPM	DBE	i-FIT	i-FIT (Norm)	Formula
Minimum:				-1.5			
Maximum:		5.0	5.0	150.0			
401.0358	401.0361	-0.3	-0.7	10.5	291.7	0.0	C11 H10 N8 O7 Cl
	401.0354	0.4	1.0	3.5	294.9	3.2	C10 H19 N6 O Cl I
	401.0340	1.8	4.5	-1.5	299.6	7.9	C9 H23 N2 O5 Cl I
	401.0342	1.6	4.0	23.5	303.1	11.5	C23 H6 N6 Cl
	401.0369	-1.1	-2.7	22.5	303.7	12.0	C27 H10 O2 Cl
	401.0343	1.5	3.7	15.5	310.7	19.1	C12 H5 N10 O7
	401.0370	-1.2	-3.0	14.5	311.5	19.8	C16 H9 N4 O9
	401.0362	-0.4	-1.0	7.5	311.9	20.2	C15 H18 N2 O3 I
	401.0351	0.7	1.7	27.5	312.5	20.8	C28 H5 N2 O2

*Figure S31: Single Mass Analysis of **2** (observed as **2**-NO_3^-) (TOF ESI-)*

3.8 Preparation of 1-(3,5-bis(trifluoromethyl)phenyl)-3-(3-((6-chloro-1,2,4,5-tetrazin-3-yl)oxy)propyl)urea **3**

General procedure B : 300mg of **S3** (0.91mmol), 137mg of dichlorotetrazine (0.91mmol), 30mL of DCM and 118µL of 2,4,6-collidine (0.96mmol). The mixture was stirred at rt for 2 hours and purified by flash chromatography, eluting with a gradient of Cyclohexane/EtOAc 8/2 to 6/4 to afford 215mg of **3** as a pink fluorescent solid. Yield: 53%.

^{1}H NMR (300MHz, Acetonitrile d3, 25°C, TMS) δ 7.98 (s, 2H, H_{arom}), 7.72 (broad s, 1H, -N-*H*), 7.52 (s, 1H, H_{arom}), 5.60 (broad s, 1H, -N-*H*), 4.71 (t, 2H, $^3J_{H-H}$= 6 Hz, -CH_2-), 3.42 (q, 2H, $^3J_{H-H}$= 6 Hz, -CH_2-), 2.12 (quint, 2H, $^3J_{H-H}$= 6 Hz, -CH_2-).

^{13}C NMR (75MHz, Acetone d6, 25°C, TMS) δ 168.0 (s, 1C, $C_{Tetrazine}$), 164.7 (s, 1C, $C_{Tetrazine}$), 155.8 (s, 1C, *C*=O), 143.6 (s, 1C, $C_{quaternary}$), 133.0-131.7 (q, 2C, $^2J_{C-F}$ = 33 Hz, *C*-CF_3), 129.9-119.1 (q, 2*C*, $^1J_{C-F}$ = 270 Hz, -CF_3), 118.4 (s, 2C, C_{arom}), 114,8 (2C, C_{arom}), 69.2 (1C, -CH_2-), 37.2 (1C, -CH_2-), 30.1 (1C, -CH_2-).

^{19}F NMR (282MHz, MeOD, 25°C) (not calibrated) δ 113.9 (6F, -CF_3).

UV-Visible (Acetonitrile) λ_{max} (ε)= 212 nm (3.72 AU), 249 nm (3.46 AU), 297 nm (0.64 AU), 324 nm (0.53 AU), 511 nm (0.12 AU).

Fluorescence (Acetonitrile) λ_{exc}= 511 nm, λ_{exc}= 560 nm

HRMS (ESI$^+$-TOF) *m/z [M+H]$^+$* calcd for $C_{14}H_{12}N_6O_2F_6Cl$ 445.0627, found 445.0614

Rf 0.17 (eluent: Cyclohexane/EtOAc 3/7)

Melting point 131.5-133°C

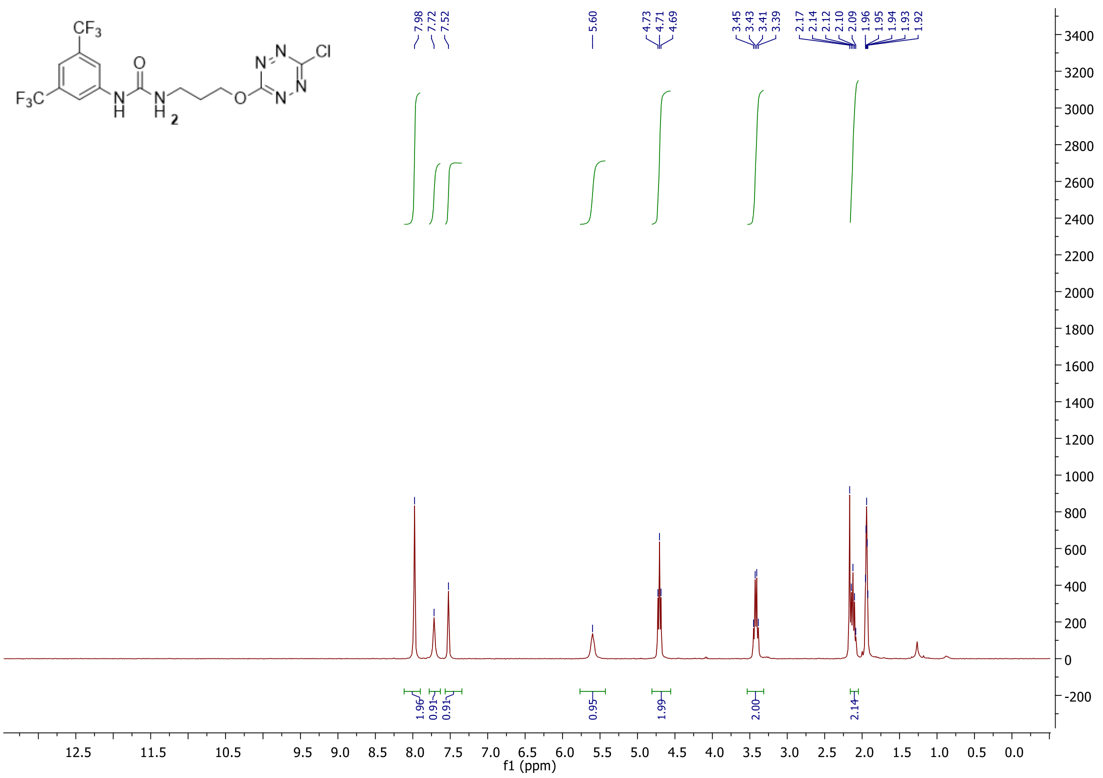

Figure S32: ^{1}H NMR (300 MHz) spectrum of **3** in Acetonitrile d3

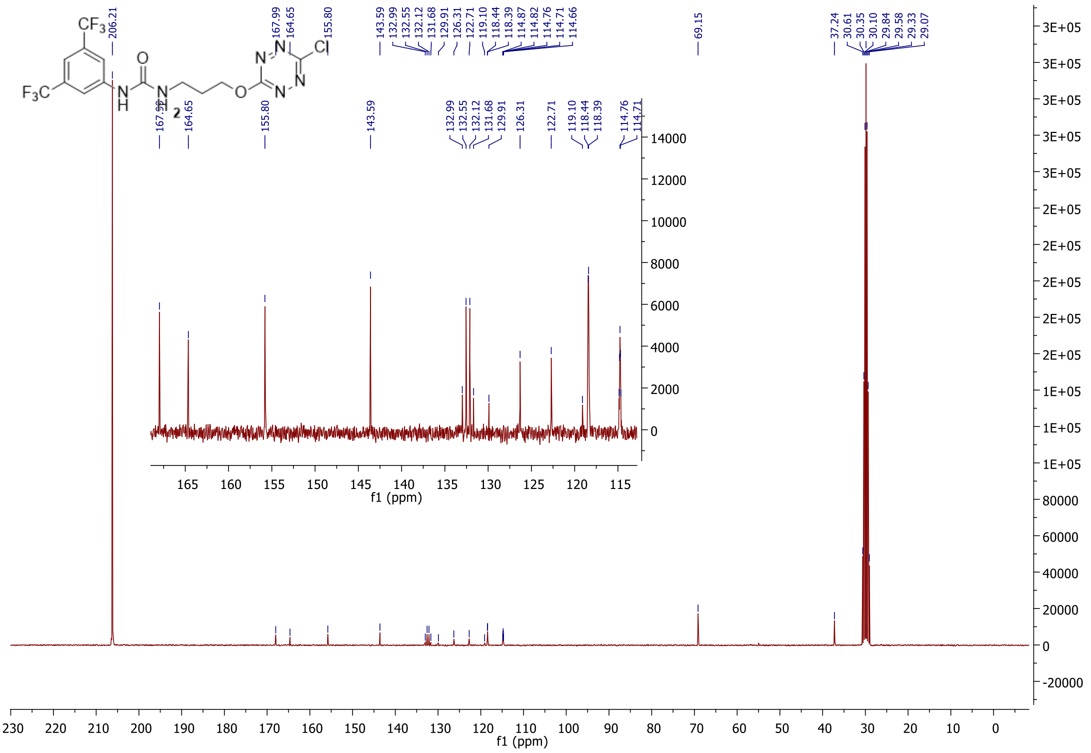

Figure S33: ^{13}C NMR (75 MHz) spectrum of **3** in Acetone d6

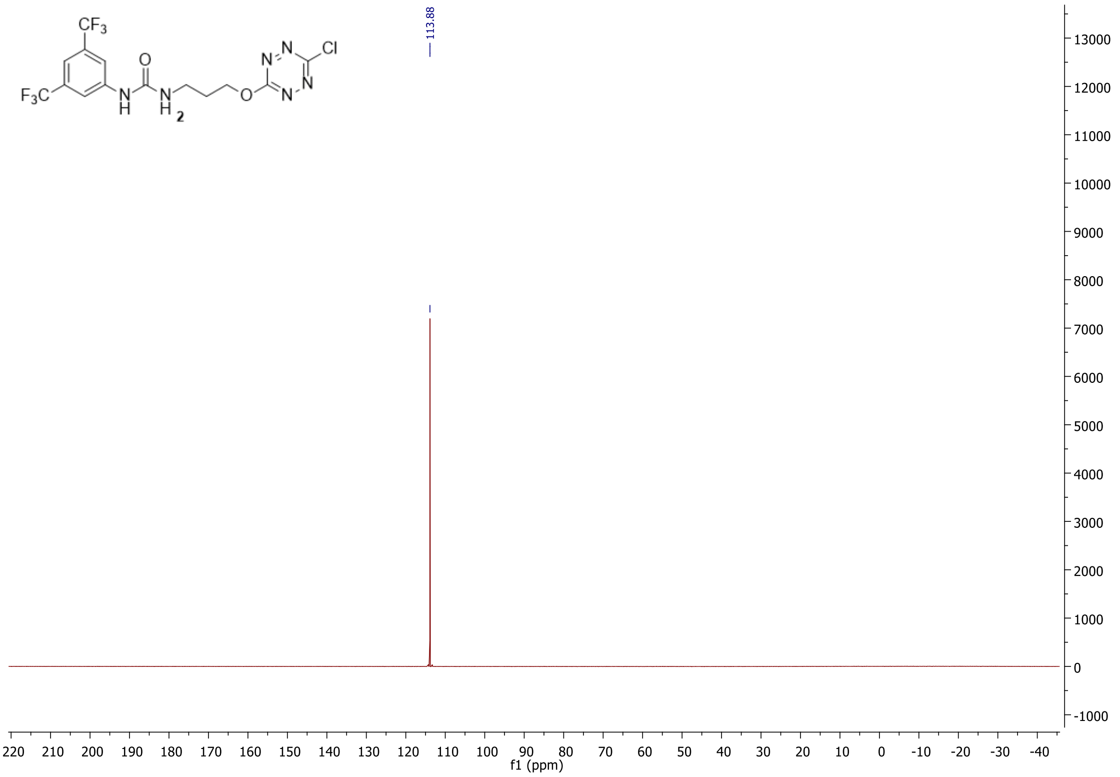

*Figure S34: ^{19}F NMR (282 MHz) spectrum of **3** in Acetone d6*

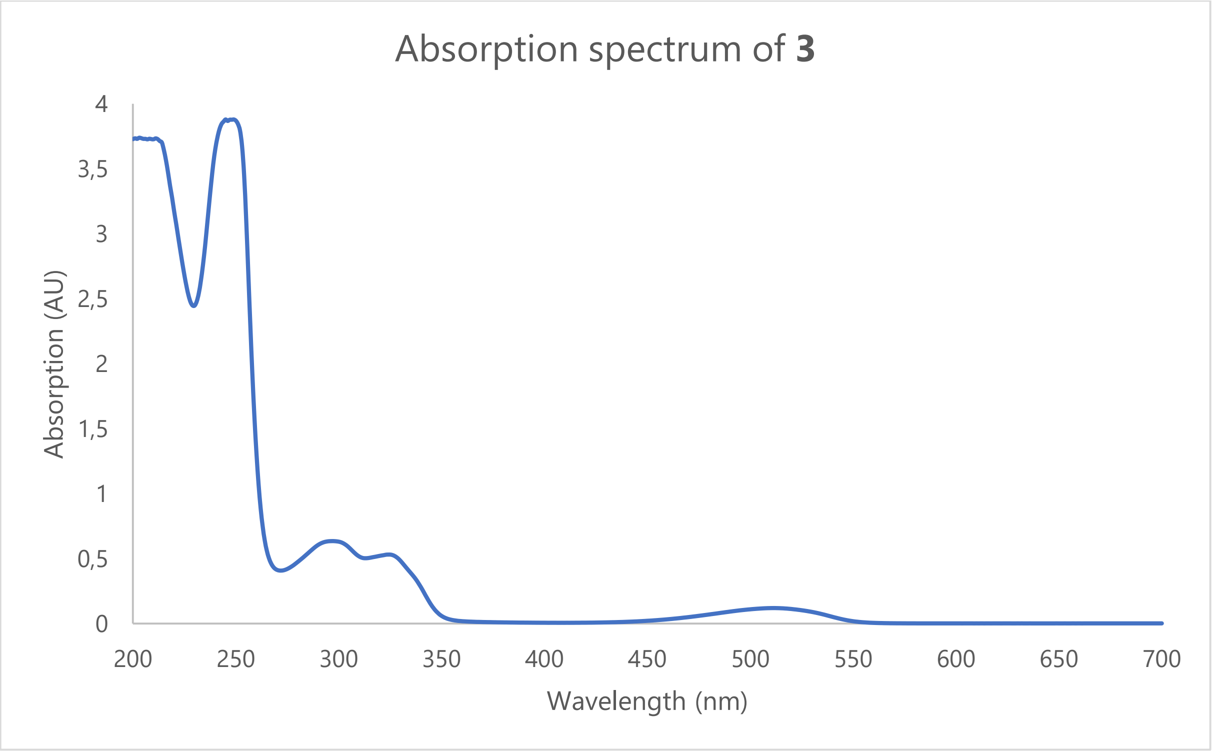

*Figure S35: Absorption spectrum of **3** in Acetonitrile*

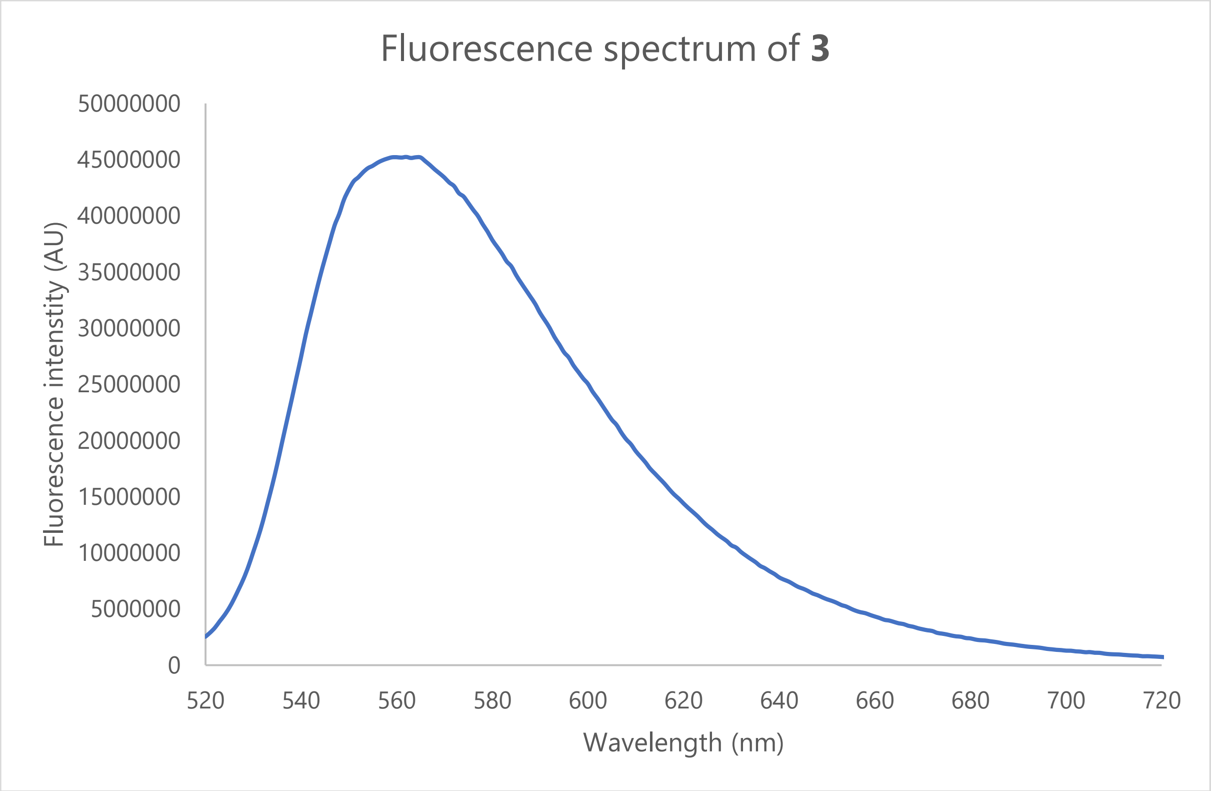

*Figure S36: Fluorescence spectrum of **3** in Acetonitrile*

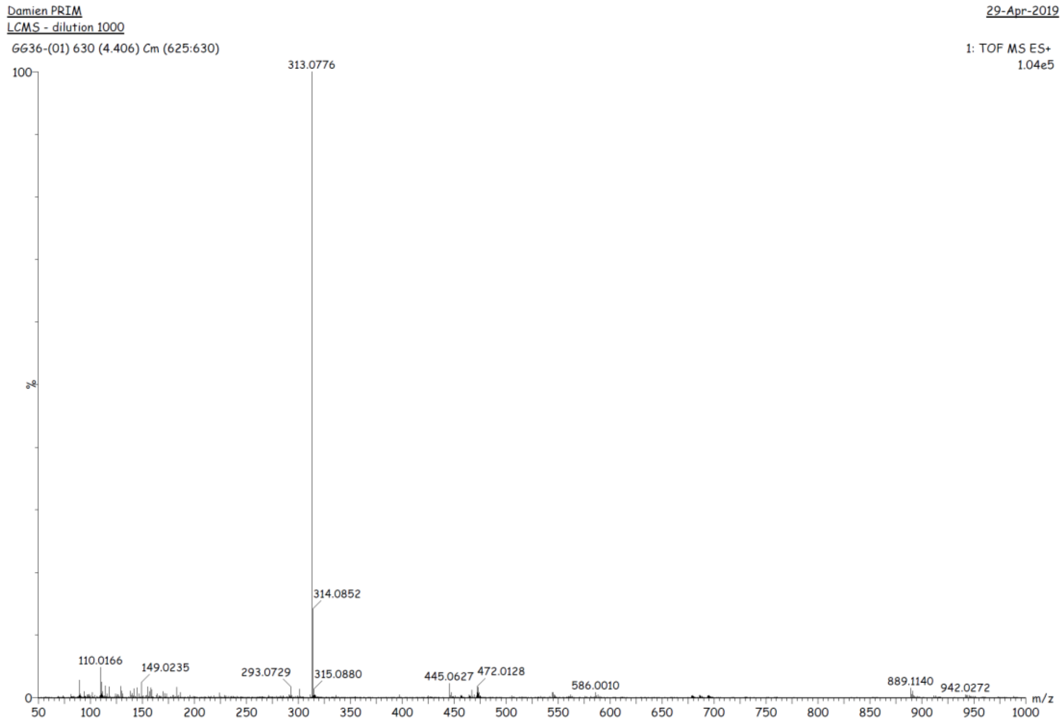

*Figure S37: Mass spectrum of **3***

Elemental Composition Report **Page 1**

Single Mass Analysis
Tolerance = 5.0 PPM / DBE: min = -1.5, max = 150.0
Element prediction: Off
Number of isotope peaks used for i-FIT = 3

Monoisotopic Mass, Even Electron Ions
6347 formula(e) evaluated with 41 results within limits (all results (up to 1000) for each mass)
Elements Used:
C: 0-100 H: 0-150 N: 0-10 O: 0-10 F: 0-6 Cl: 0-1
Damien PRIM 29-Apr-2019
LCMS - dilution 1000
GG36-(01) 630 (4.406) Cm (625:630) 1: TOF MS ES+
2.34e+003

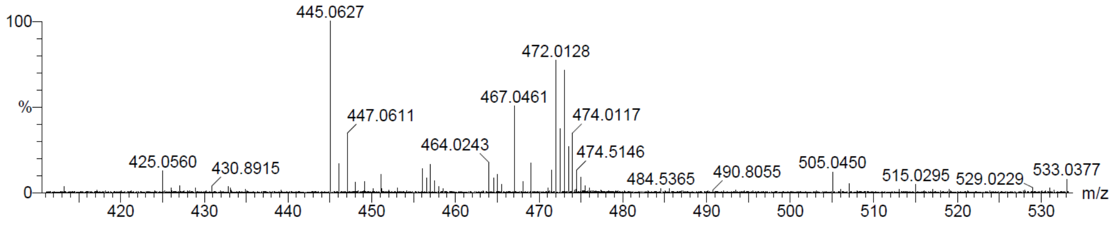

Mass	Calc. Mass	mDa	PPM	DBE	i-FIT	i-FIT (Norm)	Formula
Minimum:				-1.5			
Maximum:		5.0	5.0	150.0			
445.0627	445.0626	0.1	0.2	6.5	116.2	0.9	C15 H17 N2 O8 F3 Cl
	445.0637	-1.0	-2.2	2.5	116.8	1.5	C12 H18 N2 O9 F4 Cl
	445.0623	0.4	0.9	10.5	117.0	1.8	C13 H14 N8 O8 Cl
	445.0635	-0.8	-1.8	6.5	117.5	2.2	C10 H15 N8 O9 F Cl
	445.0614	1.3	2.9	8.5	118.2	2.9	C14 H12 N6 O2 F6 Cl
	445.0641	-1.4	-3.1	7.5	119.3	4.0	C18 H16 O4 F6 Cl
	445.0639	-1.2	-2.7	11.5	119.4	4.2	C16 H13 N6 O4 F3 Cl
	445.0630	-0.3	-0.7	11.5	121.0	5.7	C21 H15 O3 F5 Cl
	445.0614	1.3	2.9	10.5	121.0	5.7	C18 H16 N2 O7 F2 Cl
	445.0627	0.0	0.0	15.5	121.5	6.2	C19 H12 N6 O3 F2 Cl
	445.0648	-2.1	-4.7	-1.5	121.8	6.5	C9 H19 N2 O10 F5 Cl
	445.0646	-1.9	-4.3	2.5	122.1	6.9	C7 H16 N8 O10 F2 Cl
	445.0610	1.7	3.8	3.5	122.4	7.1	C8 H14 N8 O7 F4 Cl
	445.0618	0.9	2.0	15.5	123.2	7.9	C24 H14 O2 F4 Cl
	445.0631	-0.4	-0.9	22.5	123.9	8.7	C29 H14 O3 Cl
	445.0616	1.1	2.5	19.5	123.9	8.7	C22 H11 N6 O2 F Cl
	445.0622	0.5	1.1	-0.5	124.0	8.7	C5 H15 N8 O8 F5 Cl
	445.0643	-1.6	-3.6	18.5	124.1	8.8	C26 H15 O4 F Cl
	445.0607	2.0	4.5	19.5	125.7	10.5	C27 H13 O F3 Cl
	445.0616	1.1	2.5	11.5	131.2	16.0	C11 H10 N10 O9 F
	445.0605	2.2	4.9	15.5	131.4	16.1	C14 H9 N10 O8
	445.0628	-0.1	-0.2	7.5	131.4	16.1	C8 H11 N10 O10 F2
	445.0619	0.8	1.8	7.5	131.5	16.2	C13 H13 N4 O9 F4
	445.0630	-0.3	-0.7	3.5	131.6	16.3	C10 H14 N4 O10 F5
	445.0607	2.0	4.5	11.5	131.6	16.3	C16 H12 N4 O8 F3
	445.0621	0.6	1.3	16.5	131.7	16.4	C17 H8 N8 O4 F3
	445.0632	-0.5	-1.1	12.5	131.7	16.4	C14 H9 N8 O5 F4
	445.0632	-0.5	-1.1	14.5	131.8	16.5	C18 H13 N4 O10
	445.0609	1.8	4.0	20.5	131.8	16.6	C20 H7 N8 O3 F2
	445.0643	-1.6	-3.6	8.5	131.9	16.6	C11 H10 N8 O6 F5
	445.0615	1.2	2.7	0.5	132.0	16.7	C3 H11 N10 O9 F6
	445.0623	0.4	0.9	12.5	132.1	16.9	C19 H11 N2 O4 F6
	445.0612	1.5	3.4	16.5	132.2	17.0	C22 H10 N2 O3 F5
	445.0645	-1.8	-4.0	19.5	132.2	17.0	C19 H9 N8 O6
	445.0636	-0.9	-2.0	17.5	132.4	17.2	C20 H7 N6 F6
	445.0625	0.2	0.4	23.5	132.5	17.3	C27 H10 N2 O4 F
	445.0636	-0.9	-2.0	19.5	132.5	17.3	C24 H11 N2 O5 F2
	445.0613	1.4	3.1	27.5	132.6	17.4	C30 H9 N2 O3
	445.0647	-2.0	-4.5	15.5	132.6	17.4	C21 H12 N2 O6 F3
	445.0638	-1.1	-2.5	28.5	132.9	17.6	C28 H6 N6 F
	445.0640	-1.3	-2.9	24.5	133.3	18.0	C30 H9 F4

*Figure S38: Single Mass Analysis of **3** (TOF ES+)*

4. Mass Spectrometry Experiments

3D ion trap experiments

All spectra were recorded in the "Maximum Resolution mode"

MS analysis : ICC mode : "on" and acquisition time : auto.

MS/MS and MS^n analysis : ICC mode off / accumulation time 1 to 50 ms / Isolation window 1 to 12 Da depending on the precursor ion/ Fragmentation delay 40 ms.

Amplitude of fragmentation : if "smart frag off" : 0.20-1.0 depending on the ions; if "smart frag on" : amplitude 0.3 + smart frag 20%→180 % of the maximum amplitude.

Q-TOF experiments

Experiments carried out on the Xevo-G2S Q-TOf instrument (Waters) coupled to electrospray ionization in the negative mode. 10^{-5} M or 10^{-6} M mixtures of receptor/ NBu_4Cl (90/10 ACN/H2O) were introduced in the electrospray source by a syringe pump (3 µL/min).

Ion source parameters : capillary voltage 2.1-2.2 kV, sampling cone 60-80 V, source temperature 80 °C, desolvation gas temperature 150 °C, Cone gas : 20 $L \cdot hr^{-1}$; N_2 desolvation flow rate 570 $L \cdot hr^{-1}$.

Mass calibration : mass spectrum calibrated over the 20-600 mass range : calibrant NaF in the negative ion mode. Internal calibration then performed with the Leucin-Enkephalin used as lockspray mass (*m/z* 554.2615).

MS/MS : isolation window 1 mass unit (LM resolution parameter adjusted to 5) ; collision energy : from 2 to 15eV in the laboratory frame, depending on the system / collision gas : Ar

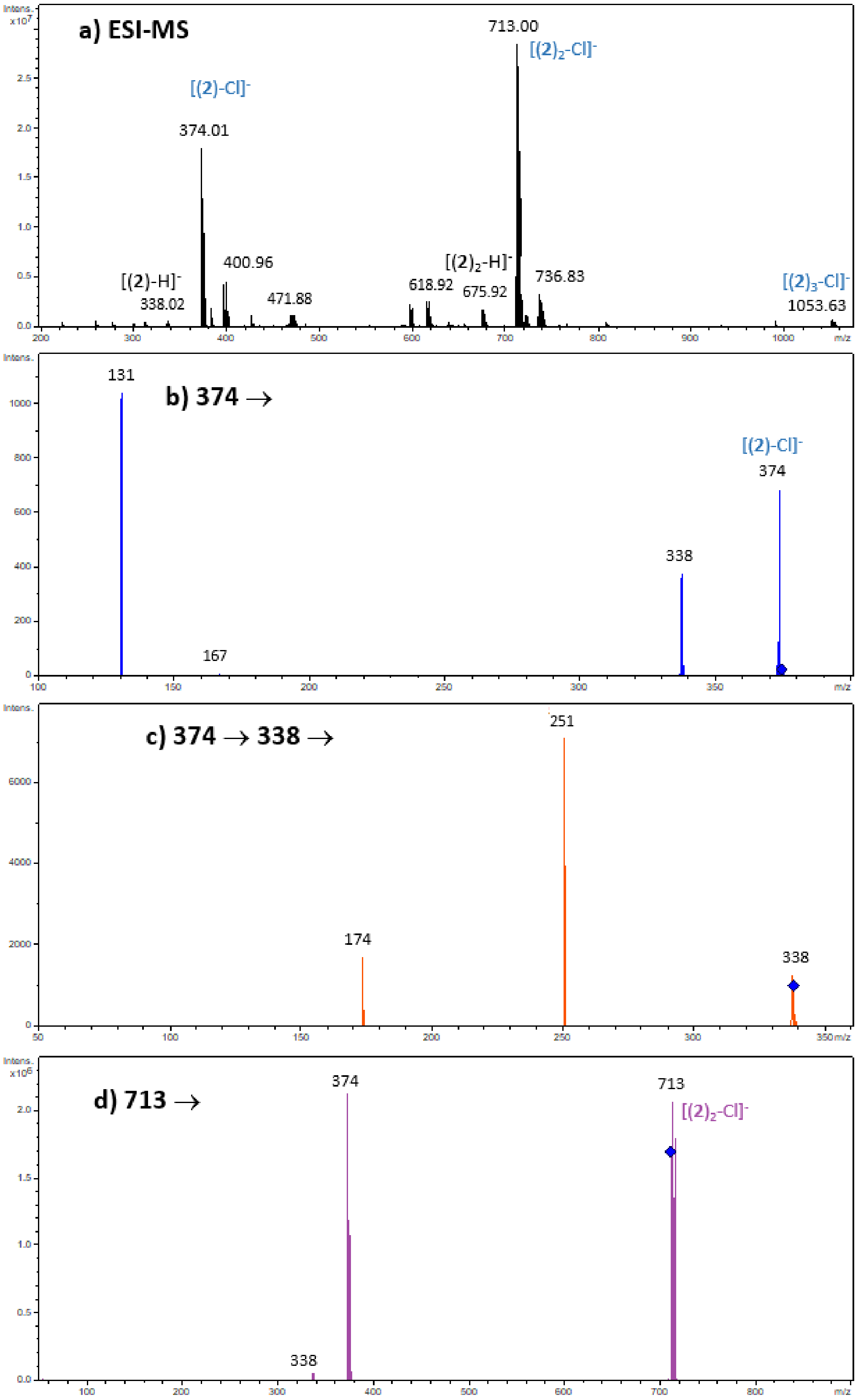

Figure S39: **a)** Electrospray mass spectrum of an equimolar (10^{-4} M) mixture of **2**/NBu_4Cl **b)** MS/MS spectrum of the [(**2**)+Cl]$^-$ ion (*m/z* 374) – **c)** MS^3 spectrum of the [(**2**)-H]$^-$ ion (*m/z* 338), d) MS/MS spectrum of the [(**2**)$_2$+Cl]$^-$ ion (*m/z* 713)

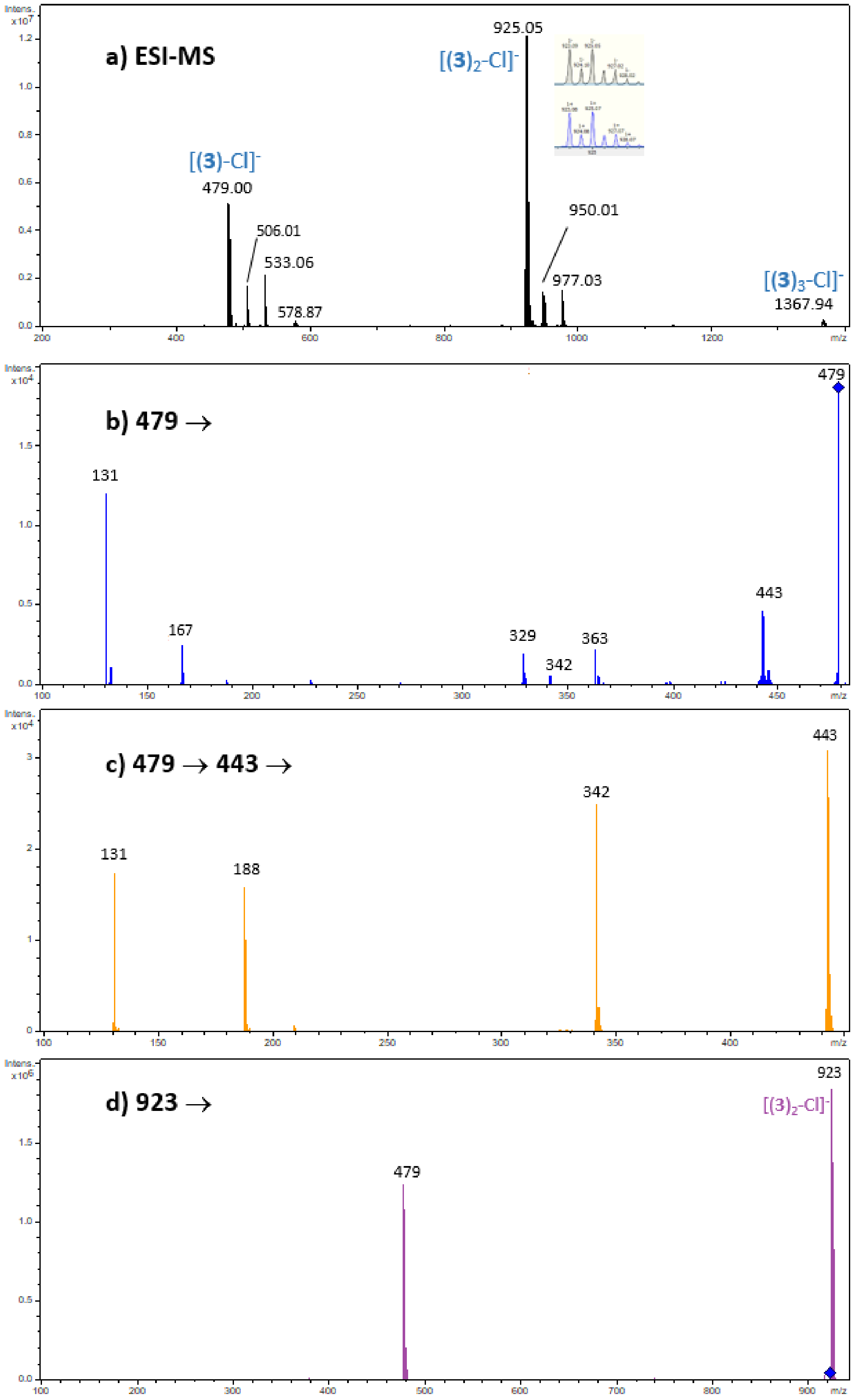

Figure S40: **a)** Electrospray mass spectrum of an equimolar (10^{-5} M) mixture of **3**/NBu_4Cl (in insert comparison between the experimental and theoretical isotopic distributions of the 1:2 adduct) **b)** MS/MS spectrum of the [(**3**)+Cl]$^-$ ion (*m/z* 479) – **c)** MS^3 spectrum of the [(**3**)-H]$^-$ ion (*m/z* 443), **d)** MS/MS spectrum of the [(**3**)$_2$+Cl]$^-$ ion (*m/z* 923)

Examples of proposed fragmentation mechanisms associated with scheme 2

- *m/z* 131 from [(**1**)+Cl]$^-$

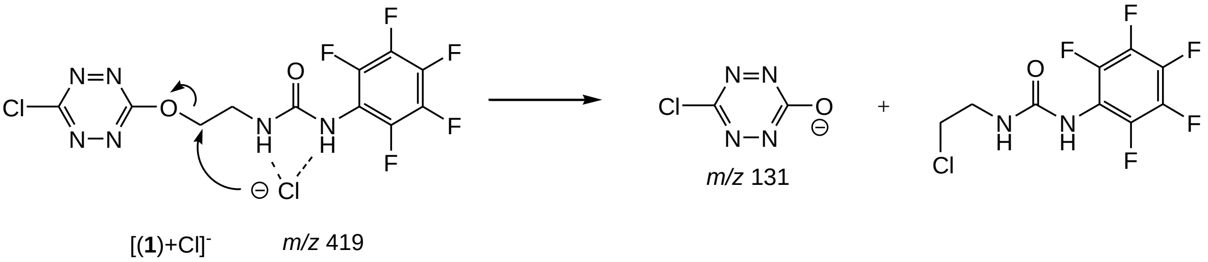

Scheme S1 Formation of [C_2ClN_4O]$^-$

- *m/z* 167 from [(**3**)+Cl]$^-$

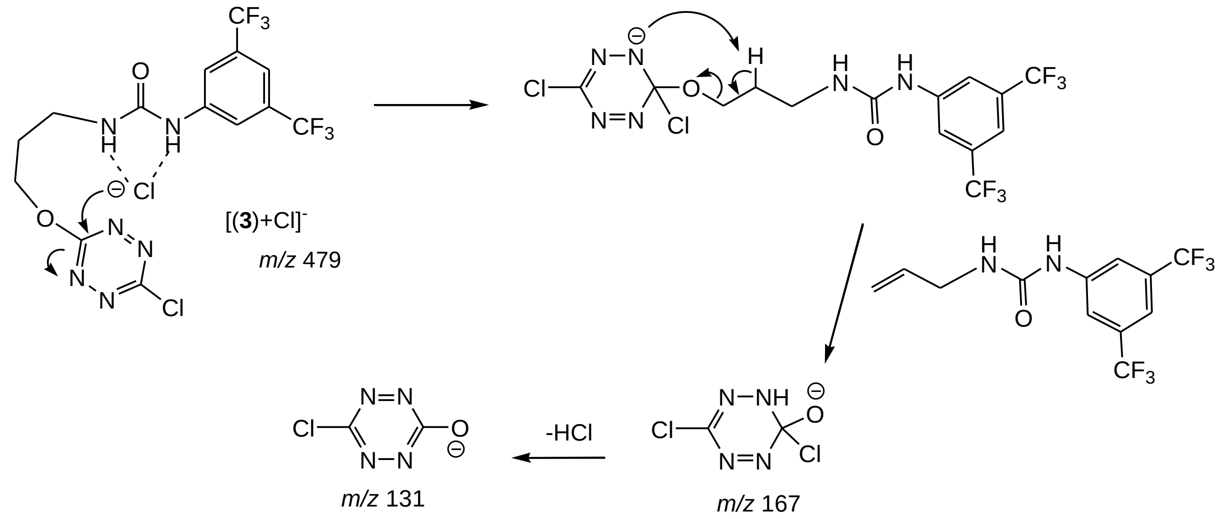

Scheme S2 Formation of [$C_2HCl_2N_4O$]$^-$

- *m/z* 338, 251 and 174 from [(**2**)+Cl]$^-$

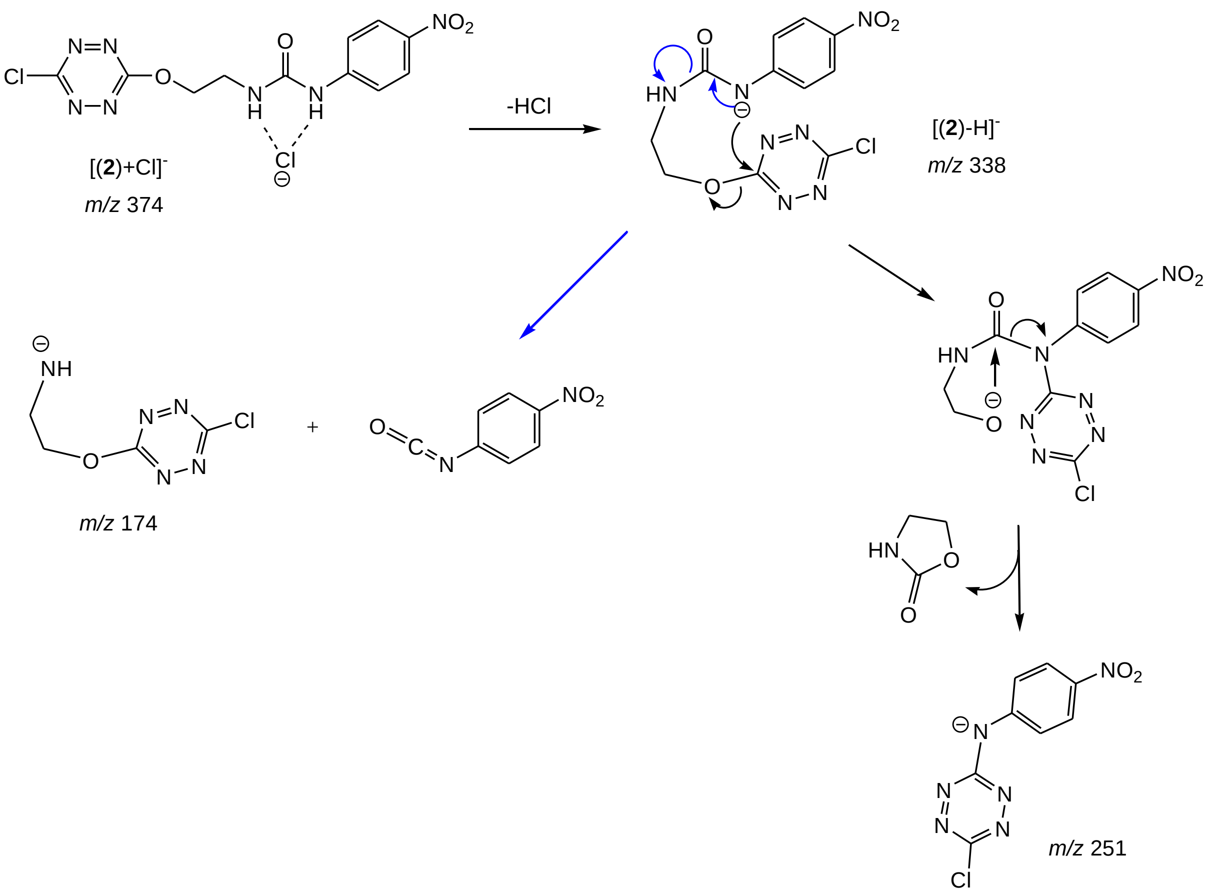

*Scheme S3 Formation of [(**2**)-H]$^-$, [$C_8H_4ClN_6O_2$]$^-$ (m/z 251) and [$C_5H_5ClN_5O$]$^-$ (m/z 174)*

QTOF accurate mass measurements

Table S1 : accurate mass measurement of the 1:1 complexes and their associated CID fragment ions

Ion /proposed fragment ions	*m/z* exp.	*m/z* calc.	Δ (*m/z*)
Receptor **1**			
[(**1**)+Cl]$^-$	418.9849	418.9855	-0.0006
$[C_2ClN_4O]^-$ *	130.9758	130.9766	-0.0008
$[C_2HCl_2N_4O]^-$ *	166.9526	166.9533	-0.0007
$[C_4H_5ClN_5O]^-$**	174.0185	174.0188	-0.0003
$[C_8ClF_5N_5]^-$	296.9767	295.9768	-0.0001
[(**1**)-H]$^-$	383.0090	383.0088	+0.0002
Receptor **2**			
[(**2**)+Cl]$^-$	374.0169	374.0177	-0.0008
$[C_8H_4ClN_6O_2]^-$	251.0087	251.0090	-0.0003
[(**2**)-H]$^-$	338.0404	338.0410	-0.0006
Receptor **3**			
[(**3**)+Cl]$^-$	479.0226	479.0230	-0.0004
$[C_5H_7ClN_5O]^-$*	188.0336	188.0345	-0.0009
$[C_{10}H_3ClF_6N_5]^-$	341.9990	341.9987	+0.0003
[(**3**)-H]$^-$	443.0457	443.0463	-0.0006

* also observed on MS/MS of [(**2**)+Cl]$^-$ and [(**3**)+Cl]$^-$

** also observed on MS/MS of [(**2**)+Cl]$^-$

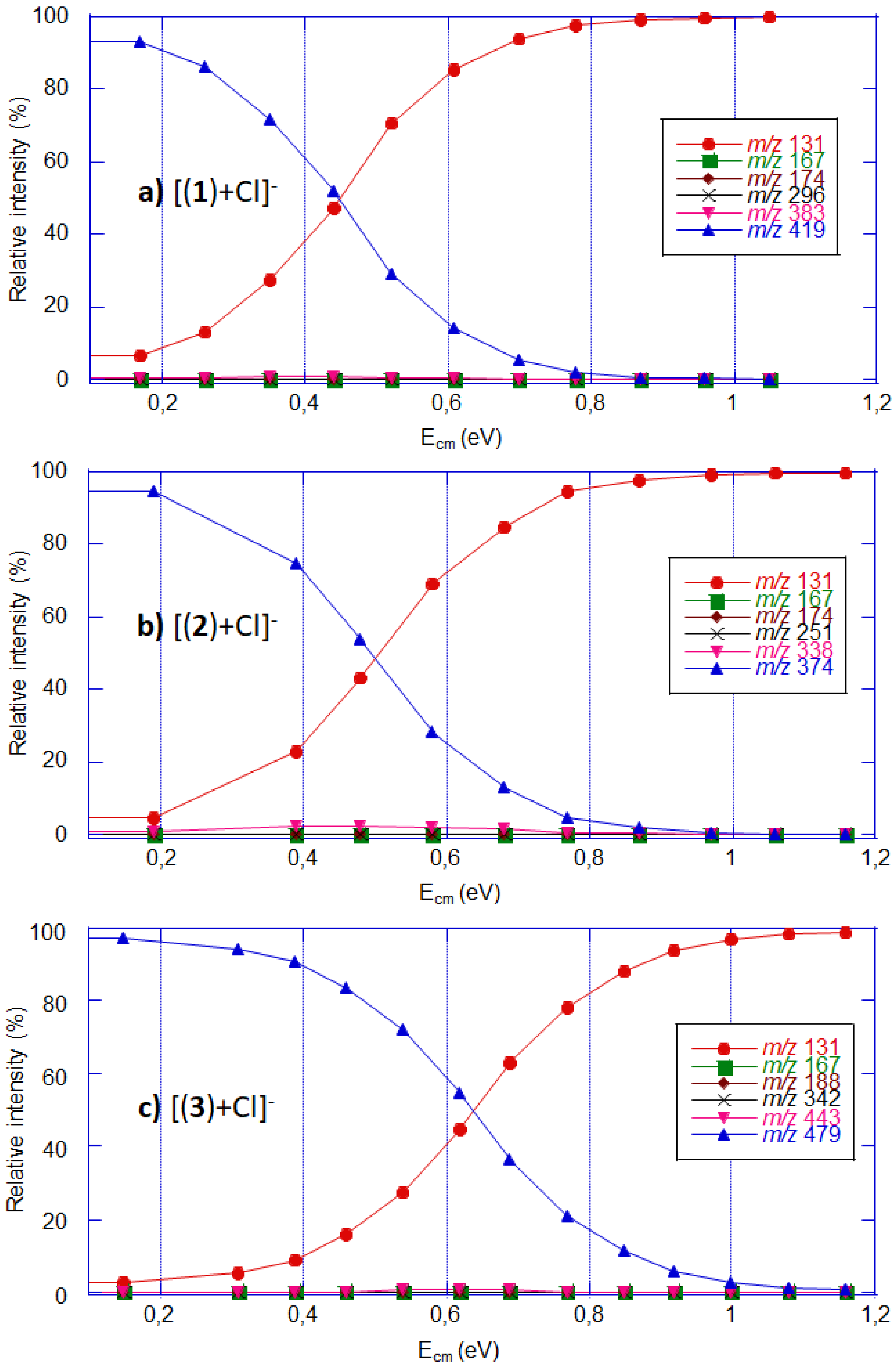

Figure S41: Intensity of fragment ions generated upon CID of **a)** [(**1**)+Cl]$^-$, **b)** [(**2**)+Cl]$^-$ and **c)** [(**2**)+Cl]$^-$ ions as a function of the center-of-mass collision energy (E_{cm}). The abundances correspond to the percentage of the total ion current for each ion.

5. NMR Titrations

5.1 Practical analysis procedure

Previously to each analysis, anion salts were solubilized into acetone and precipitated by addition of diethylether to remove water. Salts were then dried to remove residual solvents and stored in the dessicator until use.

2mL of a solution containing the anion receptor was prepared (3.5mmol/L). 500µL were placed into a new NMR tube. 1mL of stock solution was taken and desired amount of anionic guest as the tetrabutylammonium (NBu_4^+) salt was added.

^{1}H NMR spectra were calibrated to the residual proton solvent peak in MeCN-d_3 (δ = 1.94ppm) at 300 K. Plot stackings were made using MestReNova Version 6.0. Non-linear least-square curve fitting of the titration data were double checked to be a 1:1 binding model using a reported procedure [8] on Excel software and SPECFIT software.

5.2 Titration of **1** with NBu_4Cl

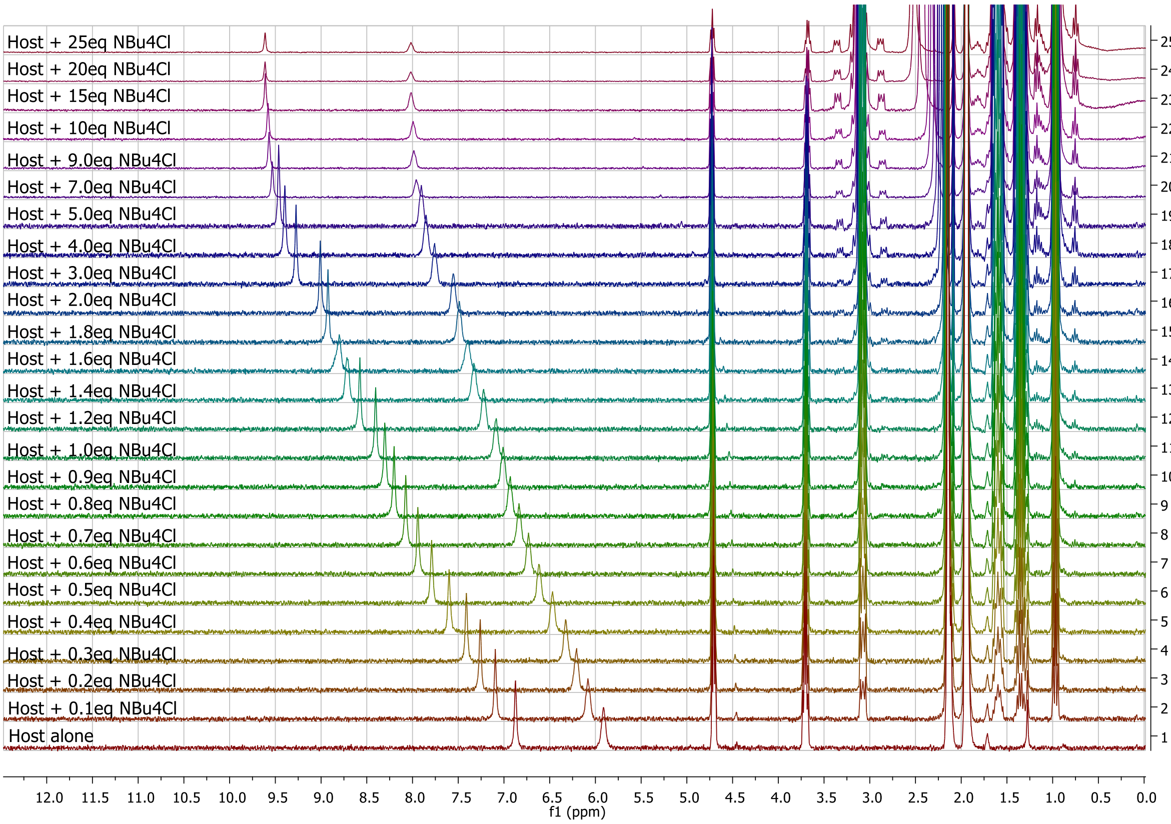

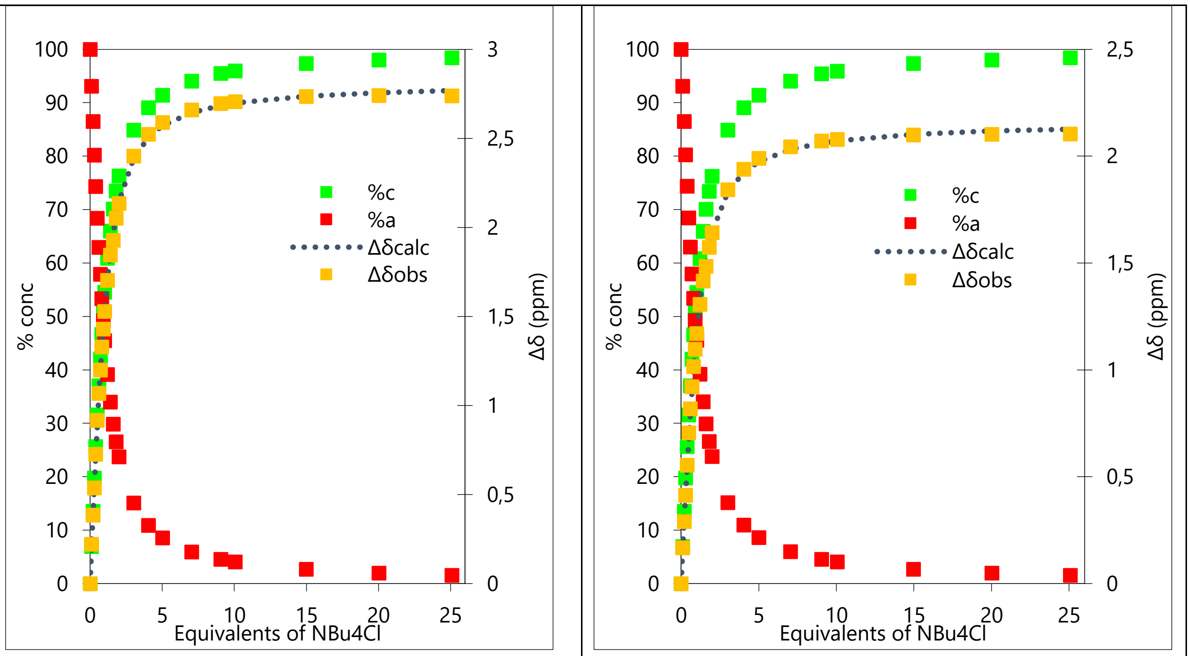

Ka = 738 L/mol
[ligand] = 0.0035 M
$\delta_{c,lim}$ = 9.69 ppm

Ka = 736 L/mol
[ligand] = 0.0035 M
$\delta_{c,lim}$ = 8.07 ppm

*Figure S42: ^{1}H NMR titration of **1** with tetrabutylammonium chloride (0 to 25 equivalents)*
%c = percentage of complex %a = percentage of free receptor Δδcalc = chemical shift calculated Δδobs = chemical shift observed

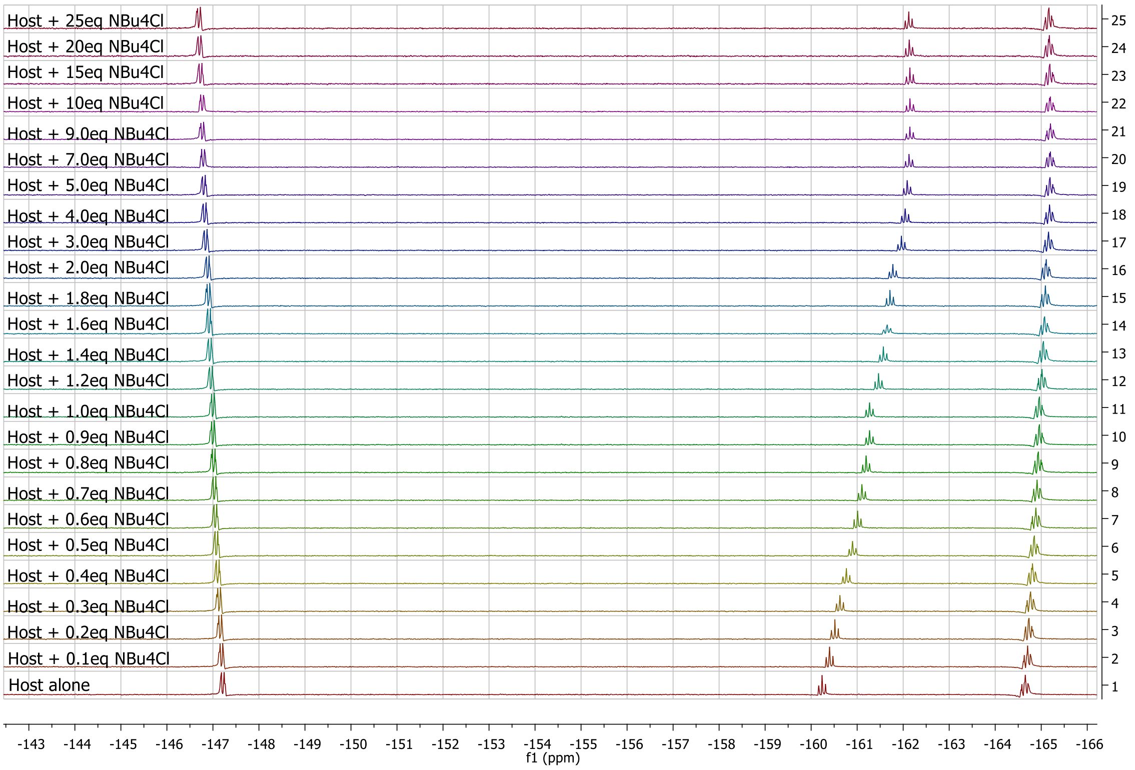

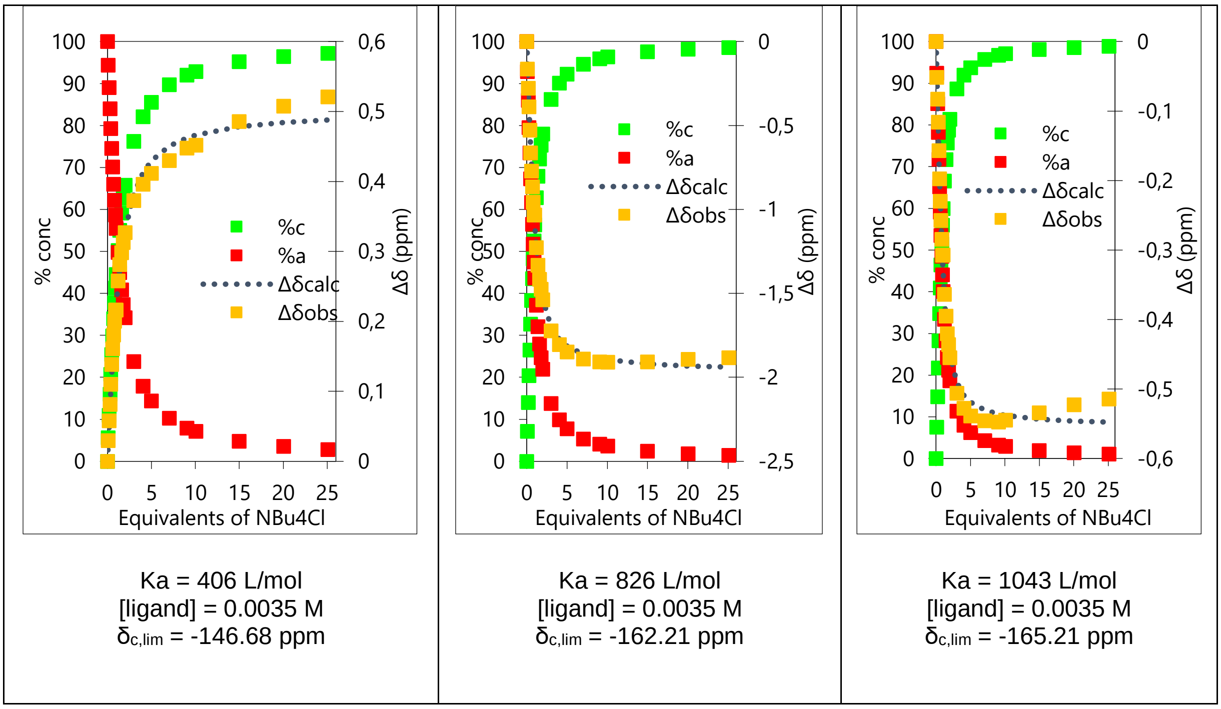

Figure S43: ^{19}F NMR titration of ***1*** *with tetrabutylammonium chloride (0 to 25 equivalents)*
%c = percentage of complex %a = percentage of free receptor Δδcalc = chemical shift calculated Δδobs = chemical shift observed

[PROGRAM]
Name = SPECFIT
Version = 3.0

[FILE]
Name = RP123+TBACL_RMN_FORMAT_SPECFIT.FAC
Path = C:\Users\Utilisateur\Desktop\
Date = 17-déc-20
Time = 14:44:51
Ncomp = 2
Nmeas = 27
Nwave = 5

[FACTOR ANALYSIS]
Tolerance = 1,000E-09
Max.Factors = 10
Num.Factors = 5
Significant = 5
Eigen Noise = 1,434E-06
Exp't Noise = 1,434E-06

#	Eigenvalue	Square Sum	Residual	Prediction
1	2,024E+06	2,960E+01	4,700E-01	Data Vector
2	2,959E+01	5,788E-03	6,597E-03	Data Vector
3	4,218E-03	1,570E-03	3,449E-03	Data Vector
4	1,470E-03	1,002E-04	8,744E-04	Data Vector
5	1,002E-04	2,672E-10	1,434E-06	Data Vector

[MODEL]
Date = 17-déc-20
Time = 14:45:05
Model = 0
Index = 3
Function = 1
Species = 3
Params = 3

[SPECIES]	[COLORED]	[FIXED]	[SPECTRUM]
1 0 0	False	False	
0 1 0	True	False	
1 1 0	True	False	

[SPECIES]	[FIXED]	[PARAMETER]		[ERROR]
1 0 0	True	0,00000E+00	+/-	0,00000E+00
0 1 0	True	0,00000E+00	+/-	0,00000E+00
1 1 0	False	2,87943E+00	+/-	1,06456E-02

[CONVERGENCE]
Iterations = 10
Convergence Limit = 1,000E-03
Convergence Found = 1,131E-04
Marquardt Parameter = 0,0
Sum(Y-y)^2 Residuals = 4,36589E-02
Std. Deviation of Fit(Y) = 1,80503E-02

[STATISTICS]
Experimental Noise = 1,434E-06
Relative Error Of Fit = 0,0147%
Durbin-Watson Factor = 0,7681
Goodness Of Fit, Chi^2 = 1,585E+08
Durbin-Watson Factor (raw data) = None
Goodness Of Fit, Chi^2 (raw data) = None

[COVARIANCE]
6,158E-04

[CORRELATION]
1,000E+00

[END FILE]

*Figure S44: Determination of binding constant using SPECFIT software for the ^{1}H and ^{19}F NMR titration of **1** with tetrabutylammonium chloride*

5.3 Titration of **2** with NBu_4Cl

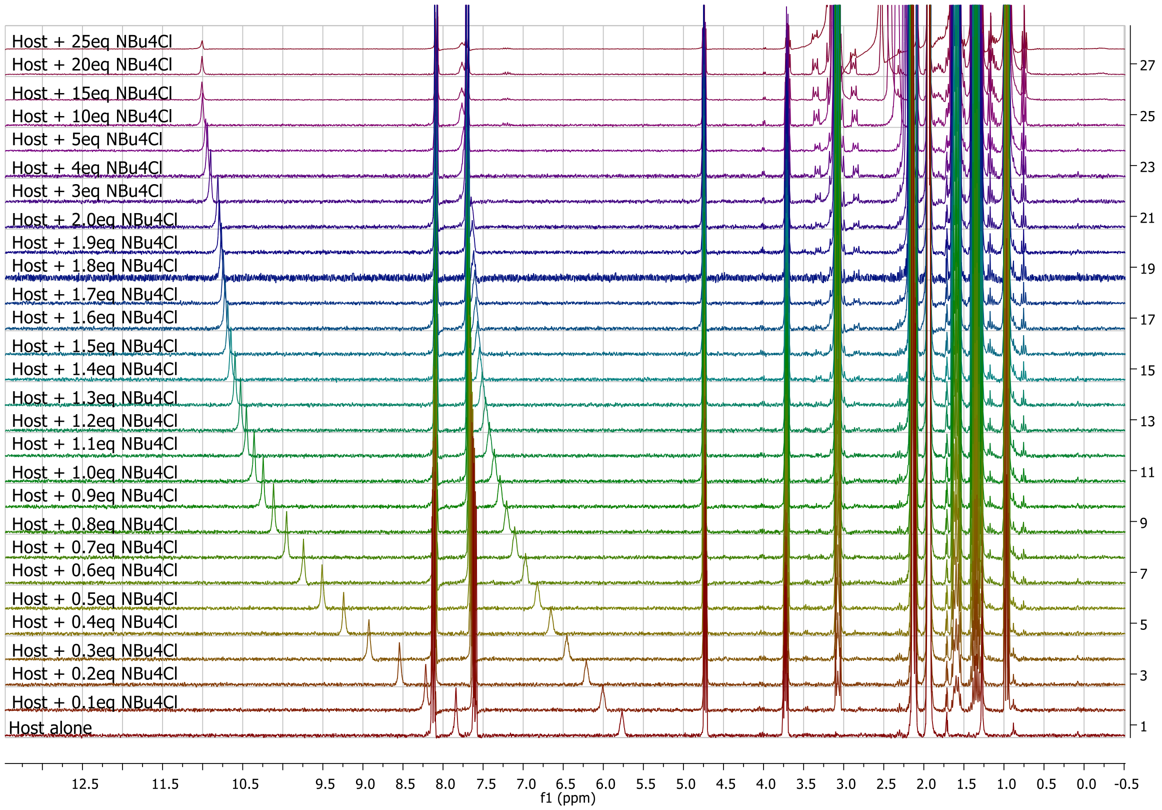

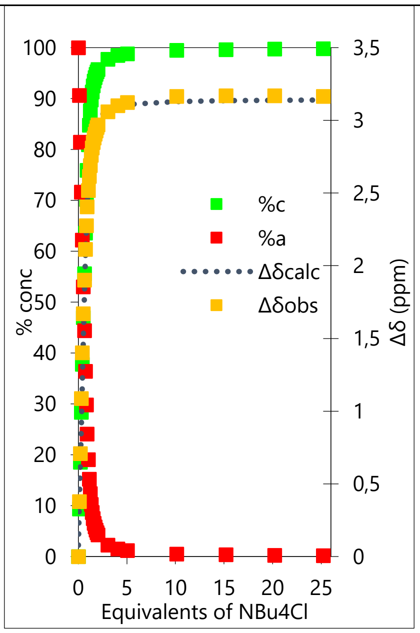

Ka = 6015 L/mol
[ligand] = 0.0035 M
$\delta_{c,lim}$ = 10.98 ppm

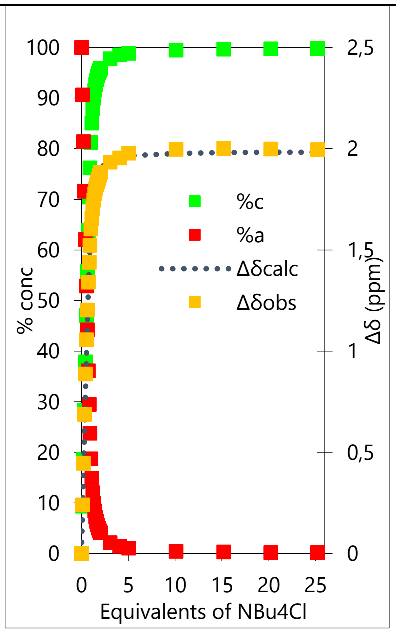

Ka = 6237 L/mol
[ligand] = 0.0035 M
$\delta_{c,lim}$ = 7.75 ppm

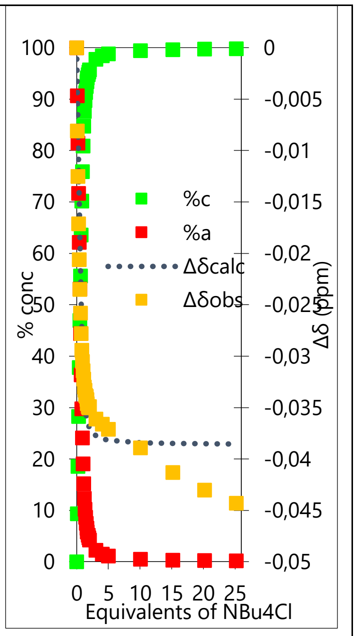

Ka = 6000 L/mol
[ligand] = 0.0035 M
$\delta_{c,lim}$ = 8.07 ppm

*Figure S45: ^{1}H NMR titration of **2** with tetrabutylammonium chloride (0 to 25 equivalents)*
%c = percentage of complex %a = percentage of free receptor Δδcalc = chemical shift calculated Δδobs = chemical shift observed

[PROGRAM]
Name = SPECFIT
Version = 3.0

[FILE]
Name = RP49+TBACL_RMN_FORMAT_SPECFIT.FAC
Path = C:\Users\Utilisateur\Desktop\
Date = 17-déc-20
Time = 14:17:24
Ncomp = 2
Nmeas = 28
Nwave = 3

[FACTOR ANALYSIS]
Tolerance = 1,000E-09
Max.Factors = 10
Num.Factors = 3
Significant = 3
Eigen Noise = 1,175E-07
Exp't Noise = 1,175E-07

#	Eigenvalue	Square Sum	Residual	Prediction
1	6,257E+03	9,390E+00	3,364E-01	Data Vector
2	9,390E+00	2,545E-04	1,762E-03	Data Vector
3	2,545E-04	1,118E-12	1,175E-07	Data Vector

[MODEL]
Date = 17-déc-20
Time = 14:17:38
Model = 0
Index = 3
Function = 1
Species = 3
Params = 3

[SPECIES]	[COLORED]	[FIXED]	[SPECTRUM]
1 0 0	False	False	
0 1 0	True	False	
1 1 0	True	False	

[SPECIES]	[FIXED]	[PARAMETER]		[ERROR]
1 0 0	True	0,00000E+00	+/-	0,00000E+00
0 1 0	True	0,00000E+00	+/-	0,00000E+00
1 1 0	False	3,69642E+00	+/-	3,96952E-02

[CONVERGENCE]
Iterations = 12
Convergence Limit = 1,000E-03
Convergence Found = 7,386E-05
Marquardt Parameter = 0,0
Sum(Y-y)^2 Residuals = 1,64503E-01
Std. Deviation of Fit(Y) = 4,45192E-02

[STATISTICS]
Experimental Noise = 1,175E-07
Relative Error Of Fit = 0,5124%
Durbin-Watson Factor = 0,2627
Goodness Of Fit, Chi^2 = 1,436E+11
Durbin-Watson Factor (raw data) = None
Goodness Of Fit, Chi^2 (raw data) = None

[COVARIANCE]
9,160E-03

[CORRELATION]
1,000E+00

[END FILE]

*Figure S46: Determination of binding constant using SPECFIT software for the ^{1}H NMR titration of **2** with tetrabutylammonium chloride*

5.4 Titration of **3** with NBu_4Cl

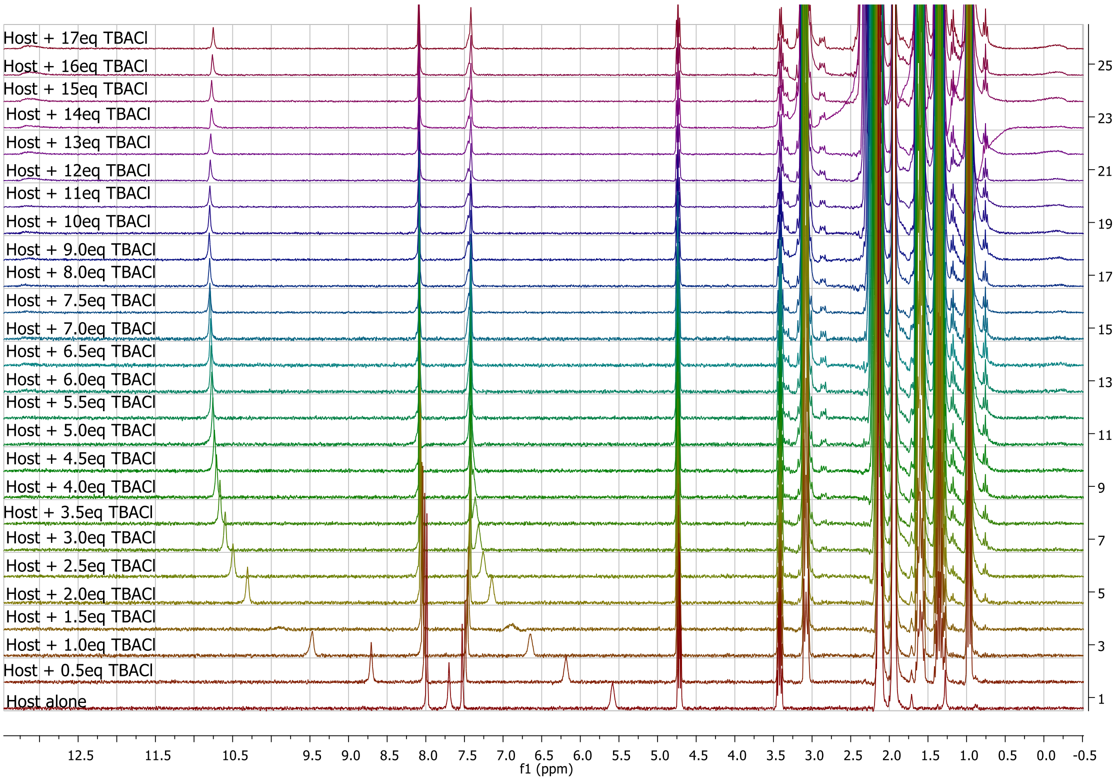

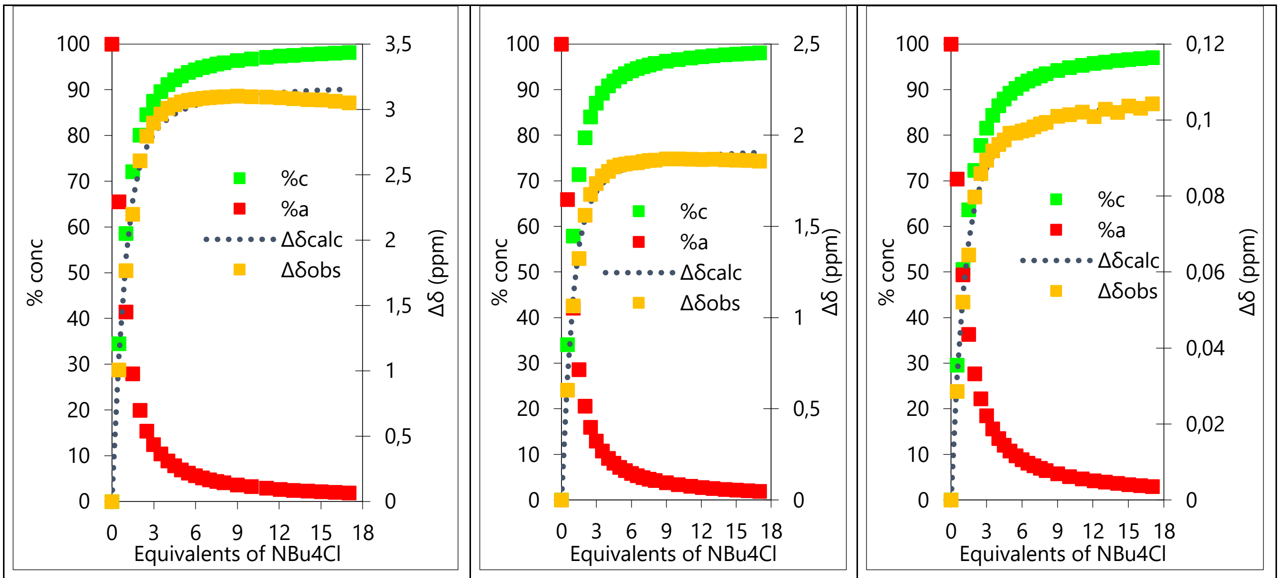

Ka = 946 L/mol<br>[ligand] = 0.0035 M<br>$\delta_{c,lim}$ = 10.91 ppm	Ka = 905 L/mol<br>[ligand] = 0.0035 M<br>$\delta_{c,lim}$ = 7.52 ppm	Ka = 579 L/mol<br>[ligand] = 0.0035 M<br>$\delta_{c,lim}$ = 8.09 ppm

*Figure S47: ^{1}H NMR titration of **3** with tetrabutylammonium chloride (0 to 18 equivalents)*
%c = percentage of complex %a = percentage of free receptor Δδcalc = chemical shift calculated Δδobs = chemical shift observed

```
[PROGRAM]
Name = SPECFIT
Version = 3.0

[FILE]
Name = GG36+TBACL_RMN_FORMAT_SPECFIT.FAC
Path = C:\Users\Utilisateur\Desktop\
Date = 17-déc-20
Time = 13:35:09
Ncomp = 2
Nmeas = 26
Nwave = 3

[FACTOR ANALYSIS]
Tolerance = 1,000E-09
Max.Factors = 10
Num.Factors = 3
Significant = 3
Eigen Noise = 8,057E-08
Exp't Noise = 8,057E-08
#  Eigenvalue  Square Sum  Residual    Prediction
1  5,913E+03   5,001E+00   2,549E-01   Data Vector
2  5,000E+00   1,303E-03   4,140E-03   Data Vector
3  1,303E-03   4,869E-13   8,057E-08   Data Vector

[MODEL]
Date = 17-déc-20
Time = 13:35:28
Model = 0
Index = 3
Function = 1
Species = 3
Params = 3

[SPECIES]        [COLORED]        [FIXED]          [SPECTRUM]
1 0 0            False            False
0 1 0            True             False
1 1 0            True             False

[SPECIES]        [FIXED]          [PARAMETER]           [ERROR]
1 0 0            True             0,00000E+00   +/-     0,00000E+00
0 1 0            True             0,00000E+00   +/-     0,00000E+00
1 1 0            False            2,99226E+00   +/-     2,66854E-02

[CONVERGENCE]
Iterations = 10
Convergence Limit = 1,000E-03
Convergence Found = 9,796E-05
Marquardt Parameter = 0,0
Sum(Y-y)^2 Residuals = 1,37631E-01
Std. Deviation of Fit(Y) = 4,22778E-02

[STATISTICS]
Experimental Noise = 8,057E-08
Relative Error Of Fit = 0,4823%
Durbin-Watson Factor = 0,4498
Goodness Of Fit, Chi^2 = 2,753E+11
Durbin-Watson Factor (raw data) = None
Goodness Of Fit, Chi^2 (raw data) = None

[COVARIANCE]
4,016E-03

[CORRELATION]
1,000E+00

[END FILE]
```

*Figure S48: Determination of binding constant using SPECFIT software for the ^{1}H NMR titration of **3** with tetrabutylammonium chloride*

6. Photophysical analysis and procedures

6.1 General practical analysis procedure

Previously to each analysis, anion salts were solubilized into acetone and precipitated by addition of diethylether to remove water. Salts were then dried to remove residual solvents and stored in the dessicator until use.

2.5mL of solution were added to a 1cm quartz glass cuvette. Aliquots of the solution containing the anion and the receptor are subsequently added to the sample cuvette for each measurement.

After blank subtraction, absorbance spectra were measured from 200 to 700nm. From the absorbance spectra were determined the absorbance maximum (510nm), that corresponds to the excitation wavelength for the emission spectra.

Emission spectra were measured from 520 nm ($\lambda_{abs,max}$+10) to 700nm using the wavelength determined before as excitation wavelength. All experiments were proceeded in temperature-controlled room at 300K.

Fluorescence decay data were analyzed using the Globals software package developed at the Laboratory for Fluorescence Dynamics at the University of Illinois at Urbana-Champaign, which includes reconvolution analysis and global non-linear least-squares minimization method.

Experimental measurements were plotted using Excel software. Determination of binding constants was done using a method developed by Valeur *et al.* [9] using non-linear least-squares minimization method.

$$Y = Y_0 + \frac{Y_{\text{lim}} - Y_0}{2}\left\{1 + \frac{c_M}{c_L} + \frac{1}{K_s c_L} - \left[\left(1 + \frac{c_M}{c_L} + \frac{1}{K_s c_L}\right)^2 - 4\frac{c_M}{c_L}\right]^{1/2}\right\}$$

Where :

Y : Measured intensity at fluorescence maximum

Y_0 : Measured intensity when no salt was added

Y_{lim} : Calculated intensity when an infinity of equivalents of salts are added

c_M : Anion concentration

c_L : Receptor concentration

K_s : Association constant of receptor/anion complex

6.2 Determination of quantum yields

	Compound **1**	Compound **2**	Compound **3**
Quantum yield	49%	30%	42%

Emission spectra of reference and compounds were recorded using the maximum absorption wavelength of the reference, Rhodamine-6G, as excitation wavelength. Fluorescence quantum yields Φ_F were determined using Rhodamine-6G as reference (Φ_F= 0.91 in ethanol). [12]

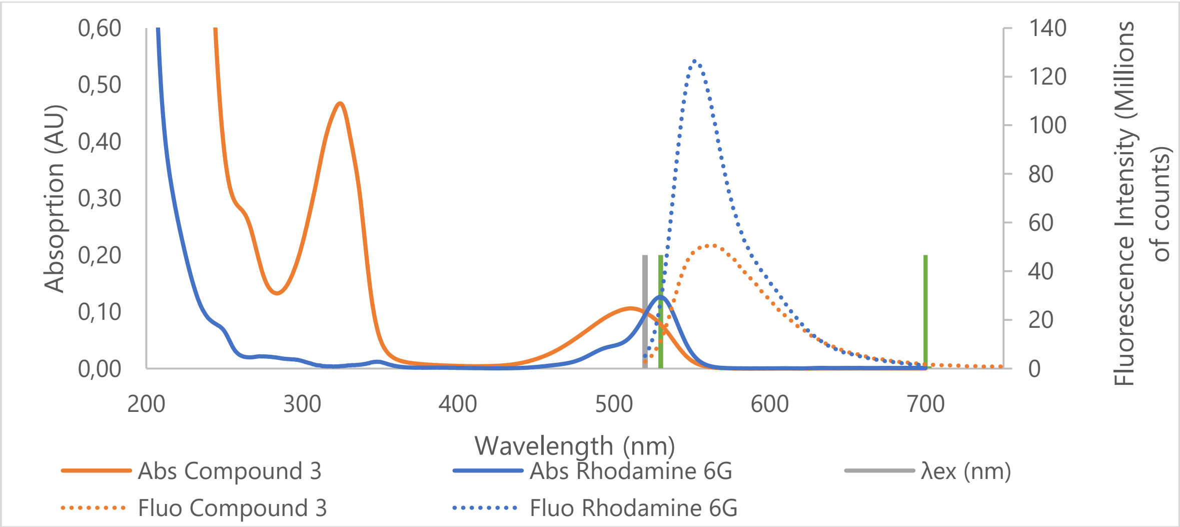

*Figure S49: Quantum yield measurement for **1** (here "compound 3" refers to **1**)*

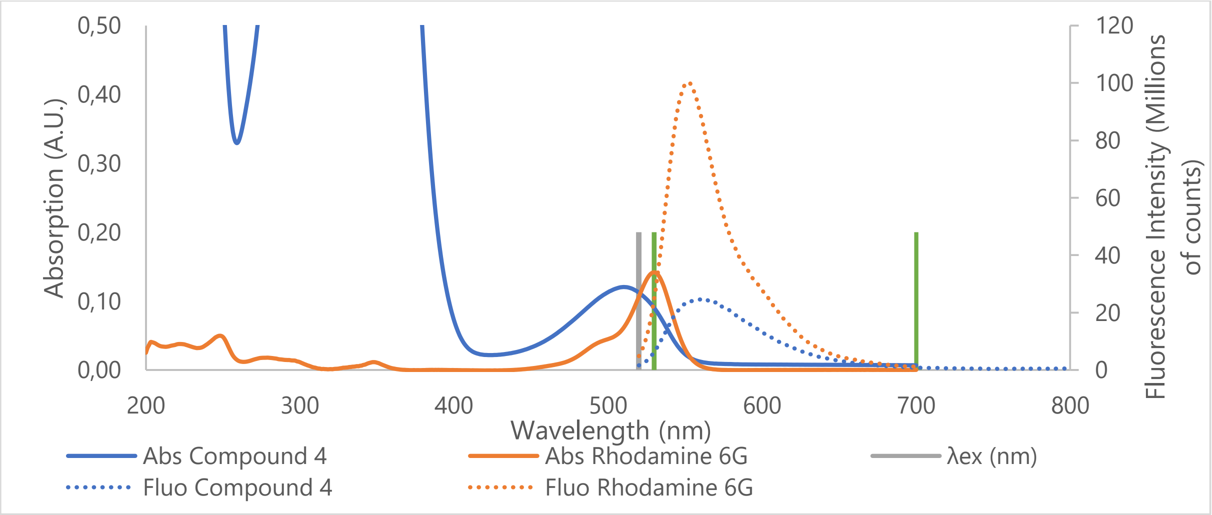

*Figure S50: Quantum yield measurement for **2** (here "compound 4' refers to **2**)*

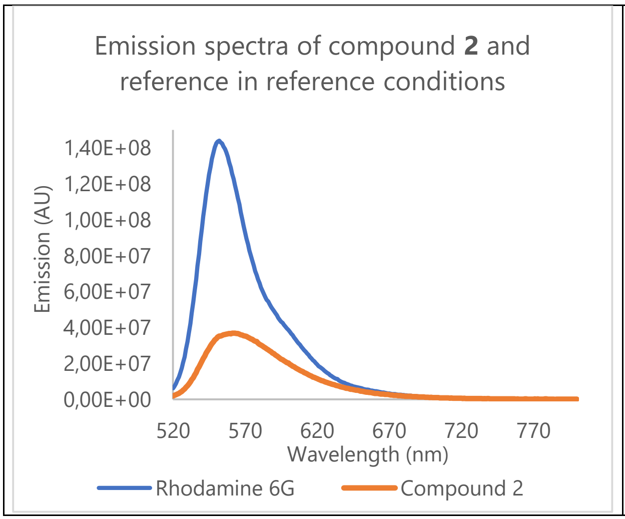

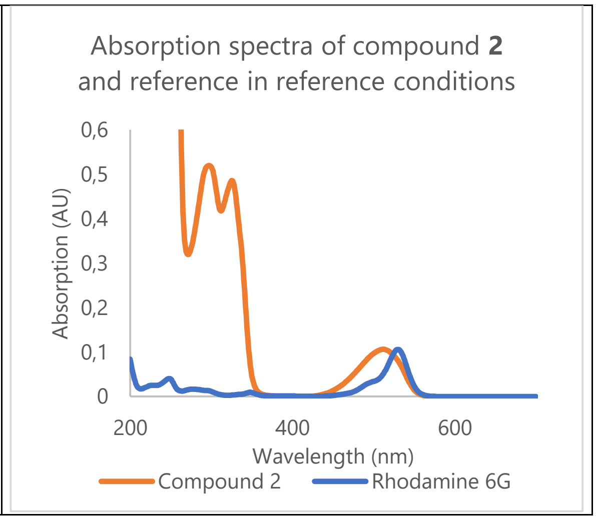

*Figure S51: Quantum yield measurement for **3** (here "compound 2" refers to **3**)*

6.3 Time dependant DFT analysis

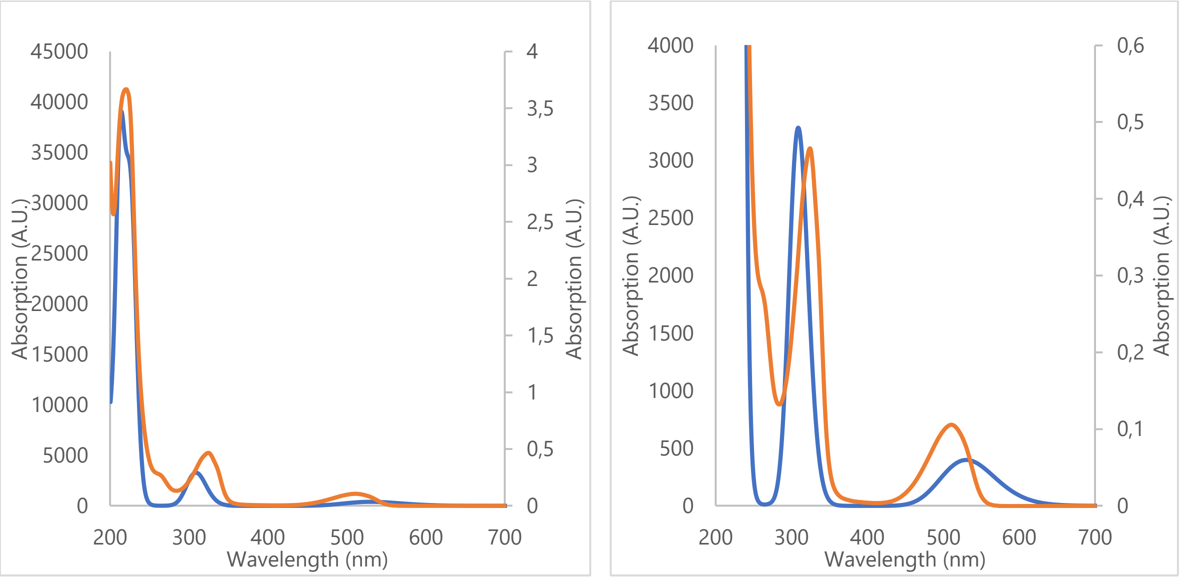

*Figure S52: Experimental (orange) and calculated (blue) absorption spectra of **1***

No.	Wavelength (nm) [a]	Osc. Strength	Major contribs	Attribution
**1**	**530 (511)**	**0.0055**	**H-1->LUMO (100%)**	**n->π* Tetrazine**
2	359	0.0	HOMO->LUMO (98%)	
3	316	0.0	H-2->LUMO (100%)	
**4**	**313**	**0.0136**	**H-4->LUMO (20%), H-3->LUMO (78%)**	**π Phenylurea ->π* Tetrazine CT**
**5**	**307 (324)**	**0.0328**	**H-4->LUMO (78%), H-3->LUMO (20%)**	**π->π* Tetrazine**
6	295	0.0	H-1->L+1 (99%)	
7	276	0.0002	H-5->LUMO (100%)	
8	251	0.0001	H-9->LUMO (90%)	
9	245	0.0	H-7->LUMO (86%)	
10	241	0.0006	H-2->L+2 (12%), HOMO->L+3 (78%)	
11	239	0.0016	H-6->LUMO (94%)	
12	238	0.0001	HOMO->L+1 (97%)	
13	237	0.0013	H-2->L+2 (33%), HOMO->L+3 (16%), HOMO->L+4 (46%)	

14	230	0.0003	H-2->L+3 (96%)	
**15**	**226 (265)**	**0.3904**	**HOMO->L+2 (86%)**	**π->π* Phenylurea**
16	226	0.0088	H-8->LUMO (89%)	
17	218	0.0002	H-2->L+1 (100%)	
18	216	0.0526	H-3->L+1 (93%)	
**19**	**212 (220)**	**0.4499**	**H-4->L+1 (92%)**	**π->π* Tetrazine**
20	206	0.0	H-13->LUMO (70%), H-12->LUMO (19%)	
21	202	0.0071	H-3->L+2 (70%), H-1->L+2 (18%)	
22	200	0.0037	H-3->L+2 (18%), H-1->L+2 (80%)	
**23**	**199 (~200)**	**0.0937**	**H-5->L+2 (33%), H-2->L+2 (22%), HOMO->L+4 (23%)**	**π->π* Phenylurea**
24	198	0.0005	H-5->L+1 (96%)	

[a] *Values in parenthesis are the corresponding experimental maxima*

*Figure S53: Calculated transitions of **1** (major transitions in bold)*

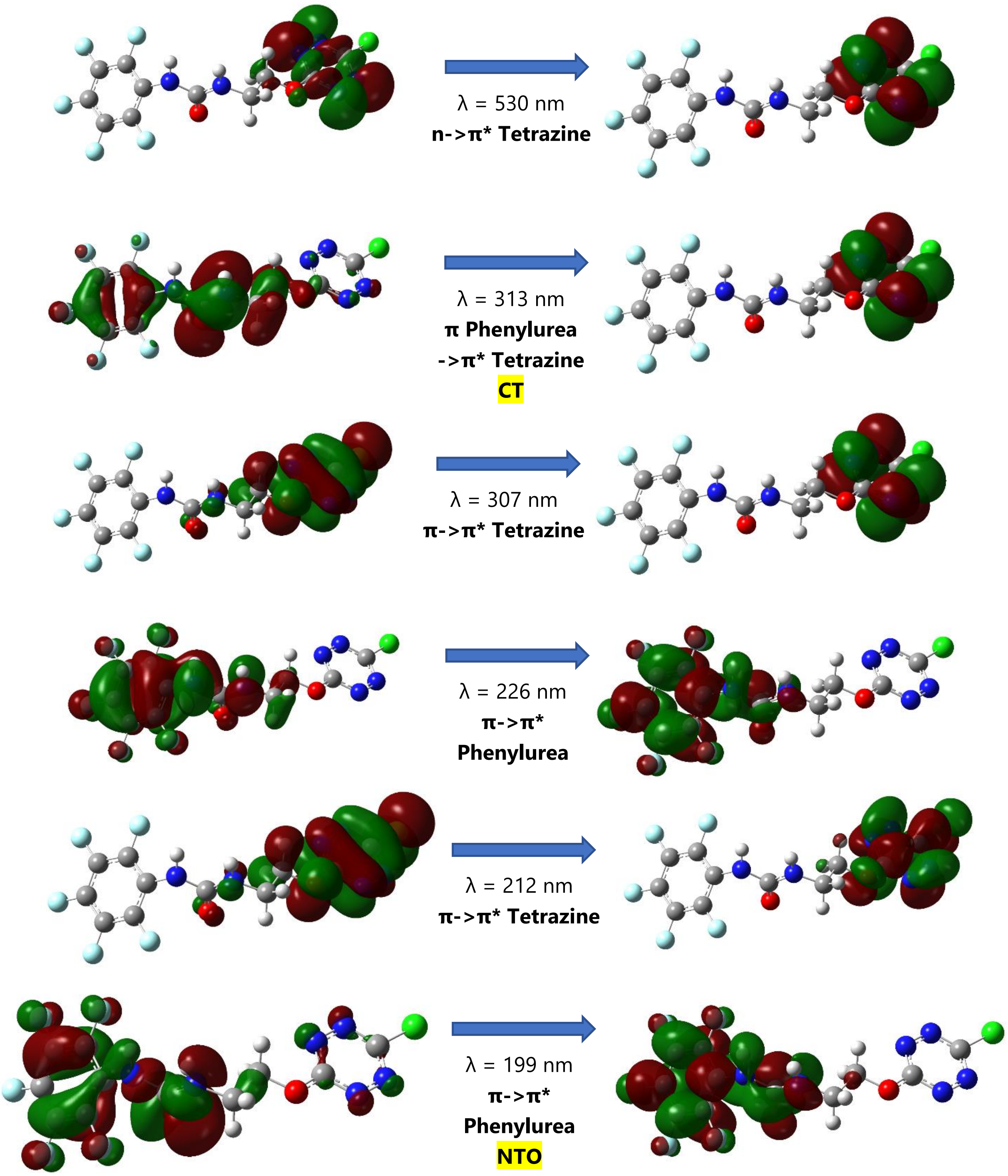

*Figure S54: Molecular orbitals involved in the main transitions of **1***

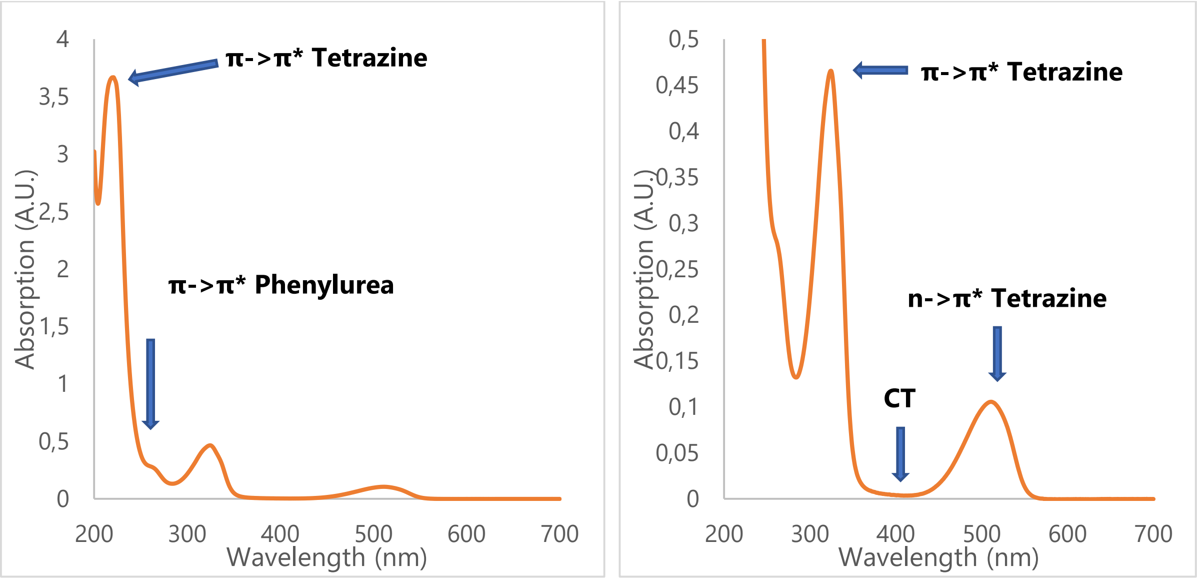

Figure S55: Attribution of principal electronic transitions involved into the different bands observed of ***1***

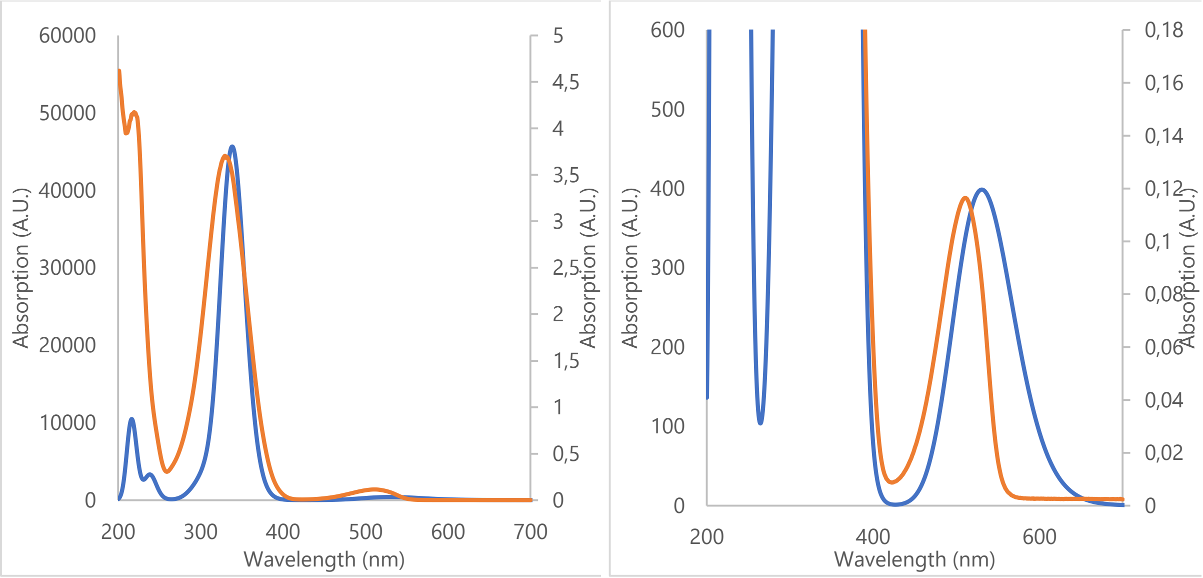

Figure S56: Experimental (orange) and calculated (blue) absorption spectra of ***2***

No.	Wavelength[a] (nm)	Osc. Strength	Major contribs	Attribution
**1**	**530 (510)**	**0.0055**	**H-1->LUMO (100%)**	**n->π* Tétrazine**
2	394	0.0	HOMO->LUMO (99%)	
**3**	**338 (329)**	**0.6253**	**HOMO->L+1 (99%)**	**π->π* Phénylurée**
4	320	0.0001	H-6->L+1 (97%)	
5	312	0.0193	H-4->LUMO (25%), H-3->LUMO (25%), H-2->LUMO (49%)	
**6**	**306 (329)**	**0.0274**	**H-4->LUMO (72%), H-2->LUMO (23%)**	**π->π* Tétrazine**
7	295	0.0	H-1->L+2 (99%)	
8	290	0.0107	H-3->L+1 (41%), H-2->L+1 (52%)	
9	290	0.0001	H-3->LUMO (71%), H-2->LUMO (28%)	
10	280	0.0002	H-5->LUMO (99%)	
11	276	0.0003	H-8->L+1 (72%), H-1->L+1 (13%)	
12	275	0.0001	H-8->L+1 (12%), H-1->L+1 (87%)	
13	264	0.0004	H-3->L+1 (49%), H-2->L+1 (40%)	
14	253	0.0004	H-5->L+1 (83%)	
15	253	0.0	HOMO->L+2 (99%)	
16	251	0.0001	H-12->LUMO (90%)	
17	245	0.0	H-10->LUMO (86%)	
18	245	0.0	H-6->LUMO (99%)	
19	240	0.0	H-4->L+1 (97%)	
**20**	**239 (219)**	**0.0445**	**HOMO->L+3 (87%)**	**π->π* Phénylurée**
21	227	0.0023	H-11->LUMO (81%), H-9->LUMO (14%)	
22	222	0.0	H-7->LUMO (100%)	
23	219	0.0	H-8->LUMO (100%)	
**24**	**216 (219)**	**0.1436**	**H-3->L+2 (29%), H-2->L+2 (61%)**	**π Phénylurée->π* Tétrazine CT**

[a] *Values in parenthesis are the corresponding experimental maxima*

*Figure S57: Calculated transitions of **2** (major transitions in bold)*

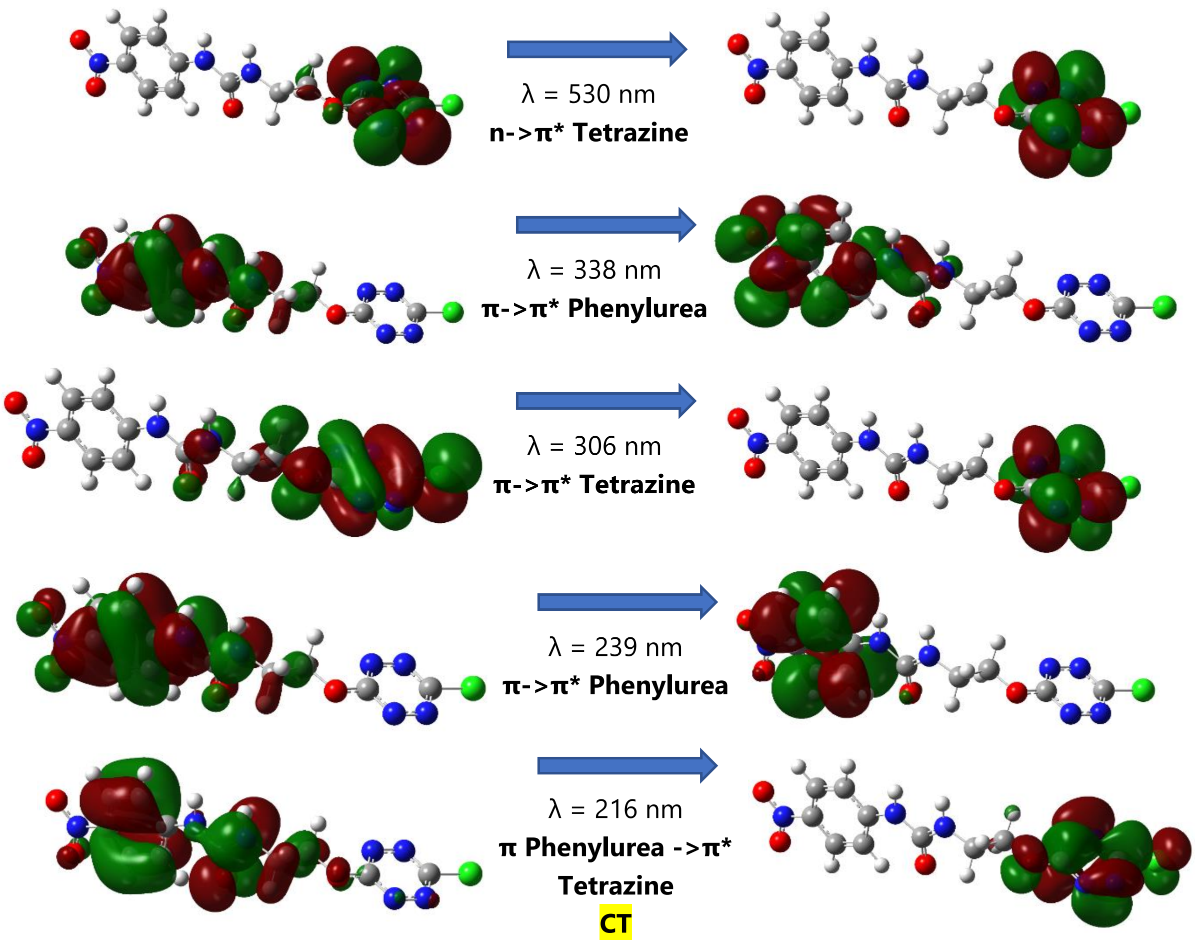

*Figure S58: Molecular orbitals involved in the main transitions of **2***

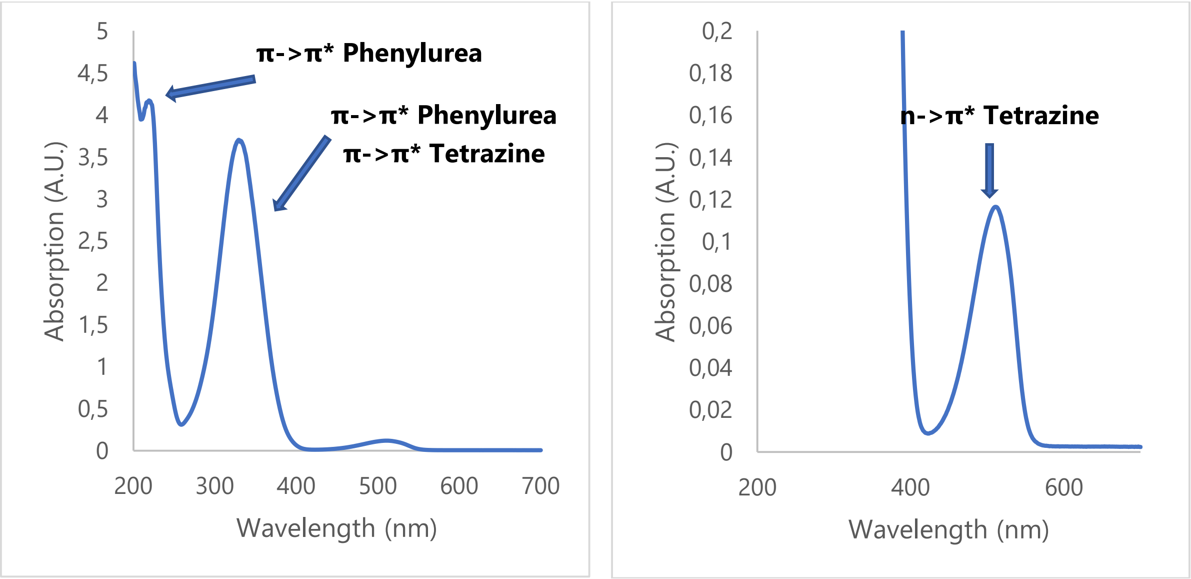

*Figure S59: Attribution of principal electronic transitions involved into the different bands observed of **2***

No.	Wavelength (nm)[a]	Osc. Strength	Major contribs	Attribution
**1**	**533 (511)**	**0.0052**	**H-1->LUMO (98%)**	**n->π* Tetrazine**
**2**	**396 (~400)**	**0.0019**	**HOMO->LUMO (99%)**	**π Phenylurea-> π* Tetrazine CT**
3	319	0.0161	H-3->LUMO (23%), H-2->LUMO (74%)	
**4**	**309 (324)**	**0.0307**	**H-3->LUMO (73%), H-2->LUMO (23%)**	**π->π* Tetrazine**
5	296	0.0	H-1->L+1 (97%)	
6	282	0.004	H-5->LUMO (35%), H-4->LUMO (63%)	
**7**	**275 (297)**	**0.0516**	**HOMO->L+2 (91%)**	**π->π* Phenylurea**
8	270	0.0003	H-5->LUMO (65%), H-4->LUMO (35%)	
9	253	0.0145	HOMO->L+1 (87%)	
10	252	0.004	H-10->LUMO (32%), H-9->LUMO (27%), H-8->LUMO (18%), HOMO->L+1 (11%)	
11	245	0.0002	H-7->LUMO (81%)	
**12**	**241 (249)**	**0.5419**	**HOMO->L+3 (93%)**	**π->π* Phenylurea**
13	228	0.0027	H-9->LUMO (31%), H-8->LUMO (48%), H-6->LUMO (16%)	
14	223	0.0196	H-2->L+2 (53%), H-1->L+2 (40%)	
15	220	0.0219	H-10->LUMO (36%), H-8->LUMO (17%), H-6->LUMO (18%), H-2->L+1 (14%)	
16	218	0.1126	H-2->L+1 (75%)	
17	217	0.0043	H-2->L+2 (37%), H-1->L+2 (59%)	
**18**	**213 (210)**	**0.3178**	**H-3->L+1 (72%)**	**π->π* Tetrazine**
19	213	0.0571	H-5->L+2 (28%), H-4->L+2 (39%), H-3->L+1 (12%)	
20	211	0.0259	H-10->LUMO (12%), H-9->LUMO (26%), H-6->LUMO (54%)	
21	209	0.0156	H-5->L+3 (11%), H-4->L+2 (10%), H-4->L+3 (19%), H-2->L+3 (30%), H-1->L+3 (17%)	
22	206	0.0023	HOMO->L+4 (91%)	
23	206	0.0007	H-14->LUMO (74%)	
24	205	0.0138	H-5->L+3 (12%), H-4->L+3 (19%), H-1->L+3 (55%)	

[a] *Values in parenthesis are the corresponding experimental maxima*

*Figure S60: Calculated transitions of **2** (major transitions in bold)*

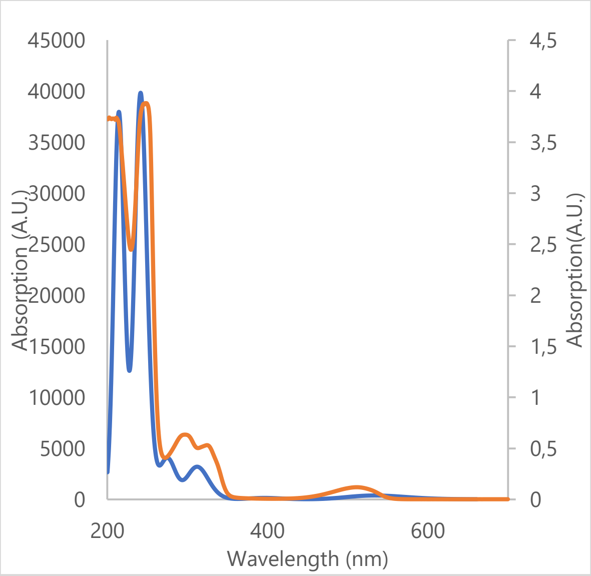

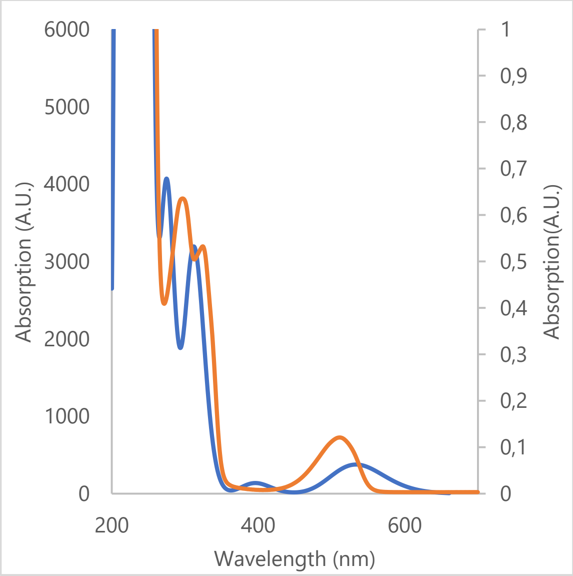

*Figure S61: Experimental (orange) and calculated (blue) absorption spectra of **3***

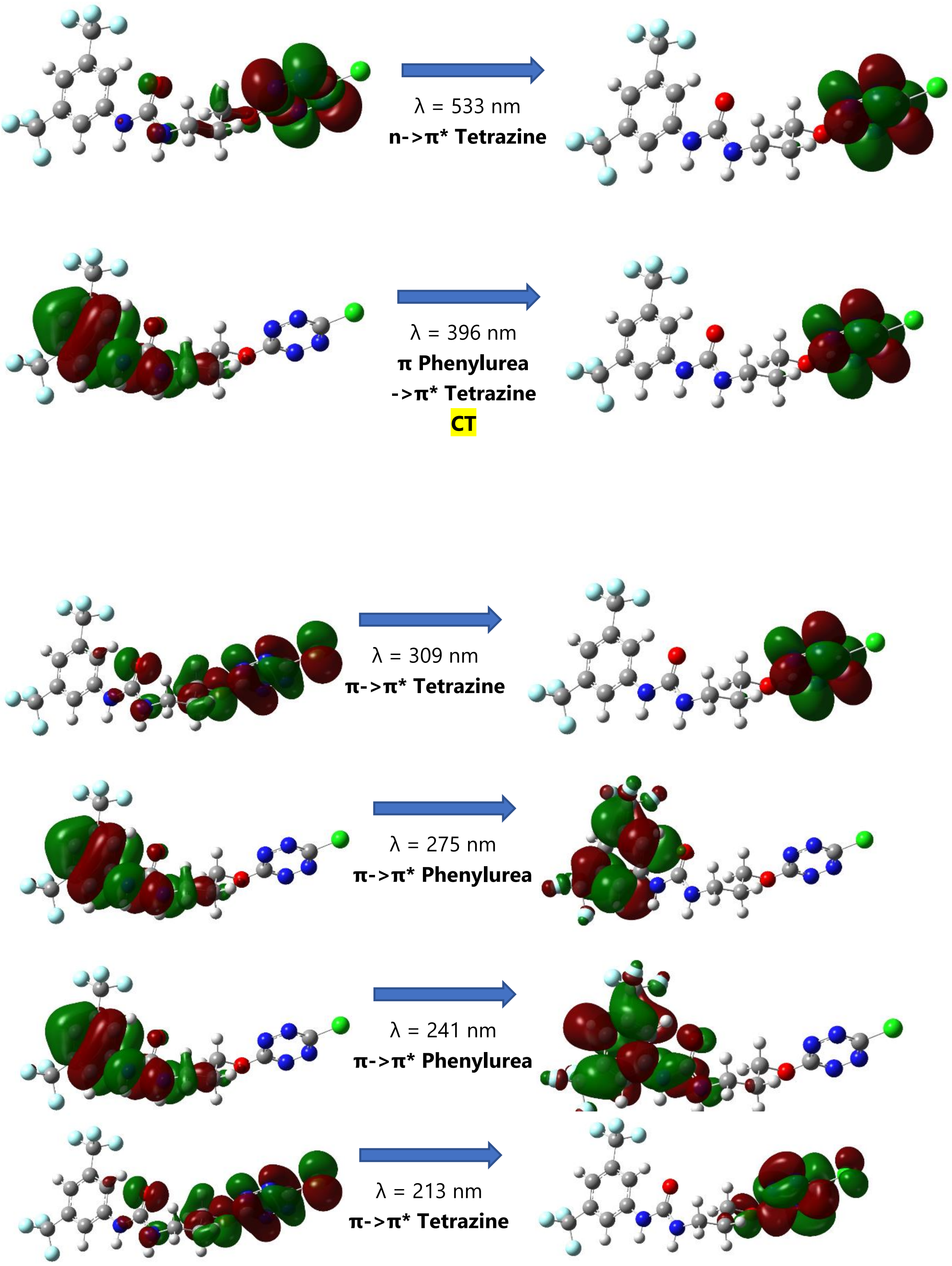

Figure S62: Molecular orbitals involved in the main transitions of ***3***

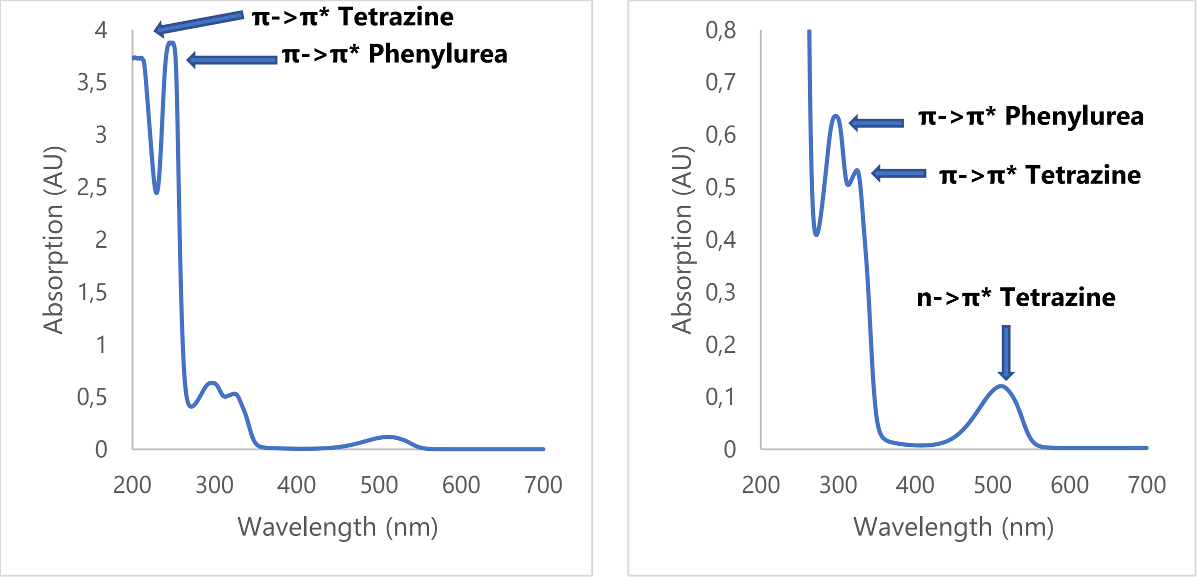

*Figure S63: Attribution of principal electronic transitions involved into the different bands observed of **3***

6.4 Titration of **1** with NBu_4Cl

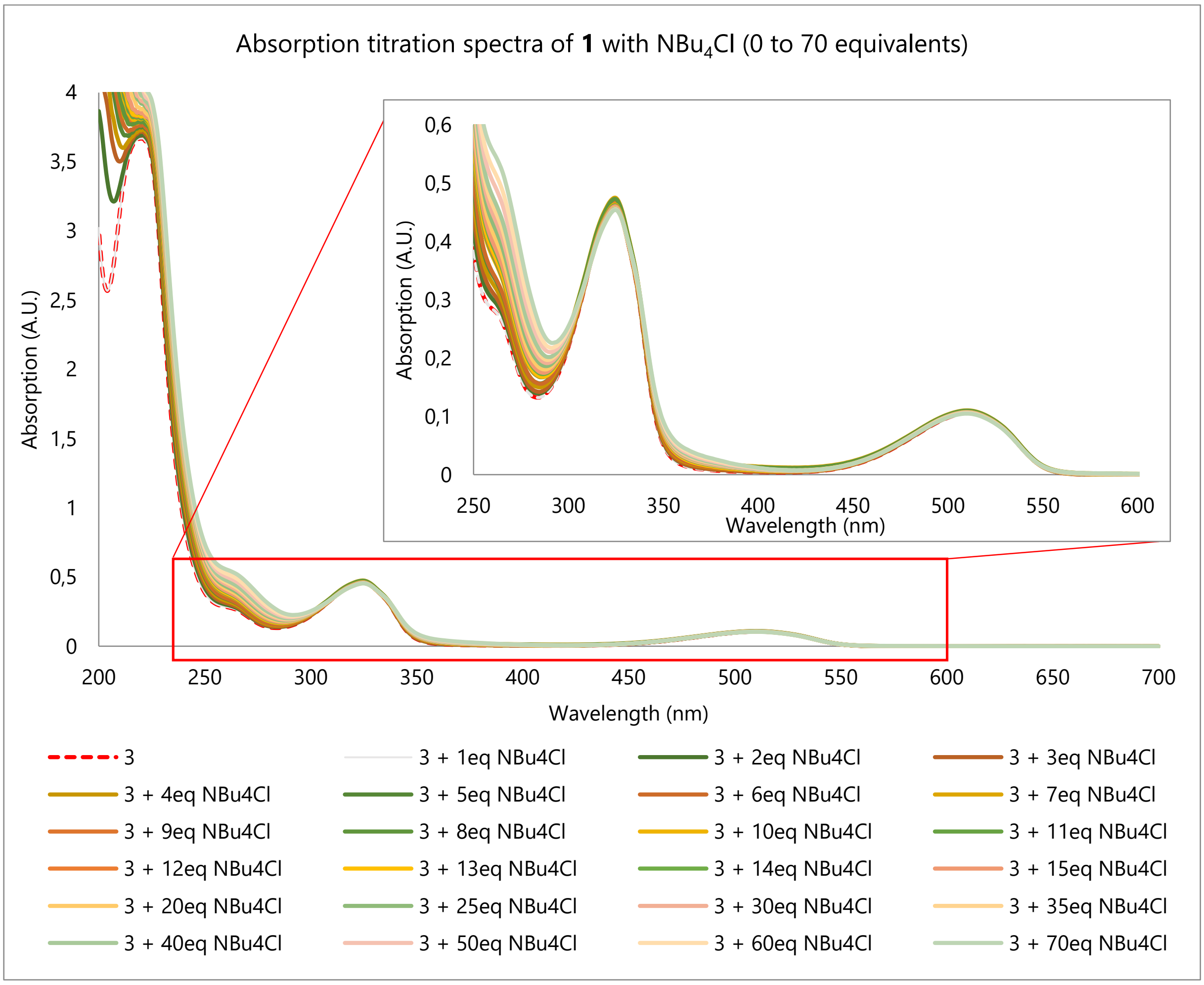

*Figure S64: Experimental UV-Visible spectra measured during the titration of **1** with NBu_4Cl (0 to 70 equivalents) (here 3 refers to 1)*

[PROGRAM]
Name = SPECFIT
Version = 3.0

[FILE]
Name = RP123+TBACL_ABS_FORMAT_SPECFIT.FAC
Path = C:\Users\Utilisateur\Desktop\
Date = 05-févr-21
Time = 12:10:49
Ncomp = 2
Nmeas = 24
Nwave = 501

[FACTOR ANALYSIS]
Tolerance = 1,000E-09
Max.Factors = 10
Num.Factors = 8
Significant = 4
Eigen Noise = 5,442E-03
Exp't Noise = 5,442E-03

#	Eigenvalue	Square Sum	Residual	Prediction
1	1,327E+04	3,629E+01	5,494E-02	Data Vector
2	3,280E+01	3,487E+00	1,703E-02	Data Vector
3	2,690E+00	7,969E-01	8,142E-03	Data Vector
4	4,409E-01	3,560E-01	5,442E-03	Data Vector
5	1,595E-01	1,965E-01	4,043E-03	Possibly Data
6	7,309E-02	1,234E-01	3,205E-03	Probably Noise
7	4,001E-02	8,340E-02	2,634E-03	Probably Noise
8	2,470E-02	5,870E-02	2,210E-03	Probably Noise

[MODEL]
Date = 05-févr-21
Time = 12:11:25
Model = 0
Index = 3
Function = 1
Species = 3
Params = 3

[SPECIES]	[COLORED]	[FIXED]	[SPECTRUM]
1 0 0	False	False	
0 1 0	True	False	
1 1 0	True	False	

[SPECIES]	[FIXED]	[PARAMETER]		[ERROR]
1 0 0	True	0,00000E+00	+/-	0,00000E+00
0 1 0	True	0,00000E+00	+/-	0,00000E+00
1 1 0	False	2,99263E+00	+/-	5,17402E-02

[CONVERGENCE]
Iterations = 10
Convergence Limit = 1,000E-03
Convergence Found = 1,787E-05
Marquardt Parameter = 0,0
Sum(Y-y)^2 Residuals = 7,27977E+00
Std. Deviation of Fit(Y) = 2,46066E-02

[STATISTICS]
Experimental Noise = 5,442E-03
Relative Error Of Fit = 2,3395%
Durbin-Watson Factor = 0,7131
Goodness Of Fit, Chi^2 = 2,045E+01
Durbin-Watson Factor (raw data) = None
Goodness Of Fit, Chi^2 (raw data) = None

[COVARIANCE]
1,601E-02

[CORRELATION]
1,000E+00

[END FILE]

*Figure S65 : Determination of binding constant using SPECFIT software for the absorption titration of **1** with NBu_4Cl*

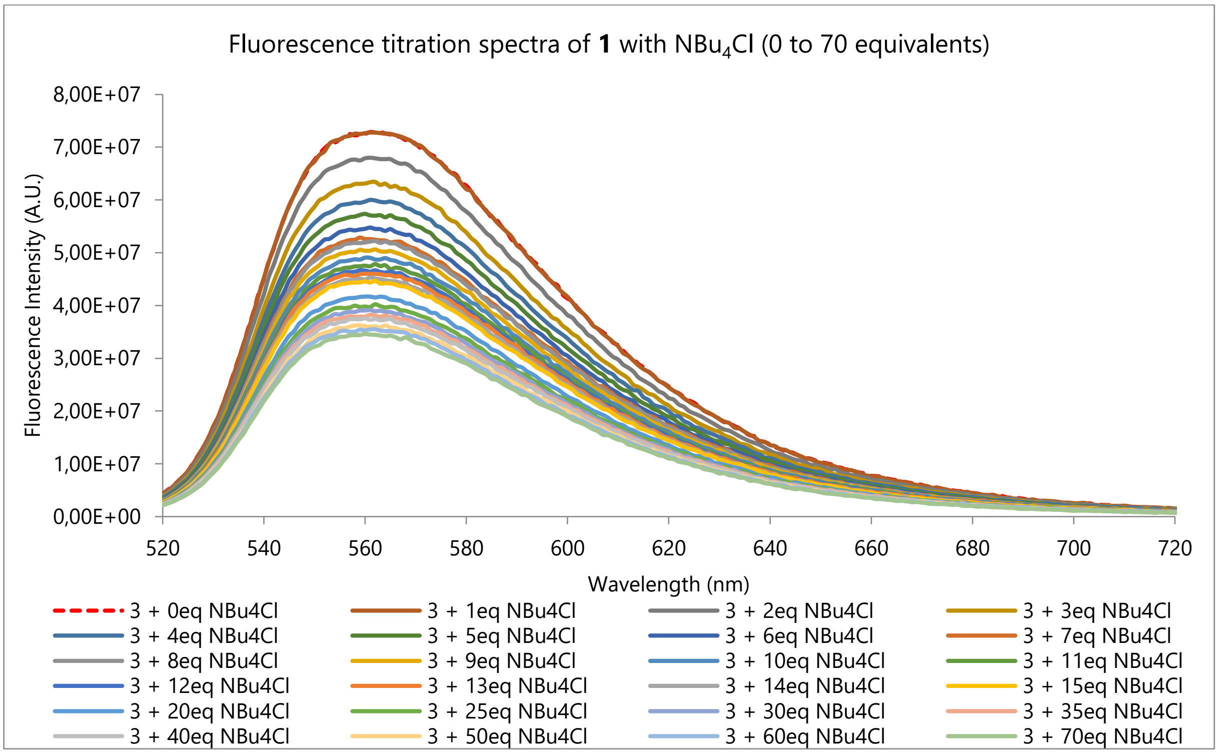

*Figure S66: Experimental fluorescence spectra measured during the titration of **1** with NBu_4Cl (0 to 70 equivalents) (here 3 refers to 1)*

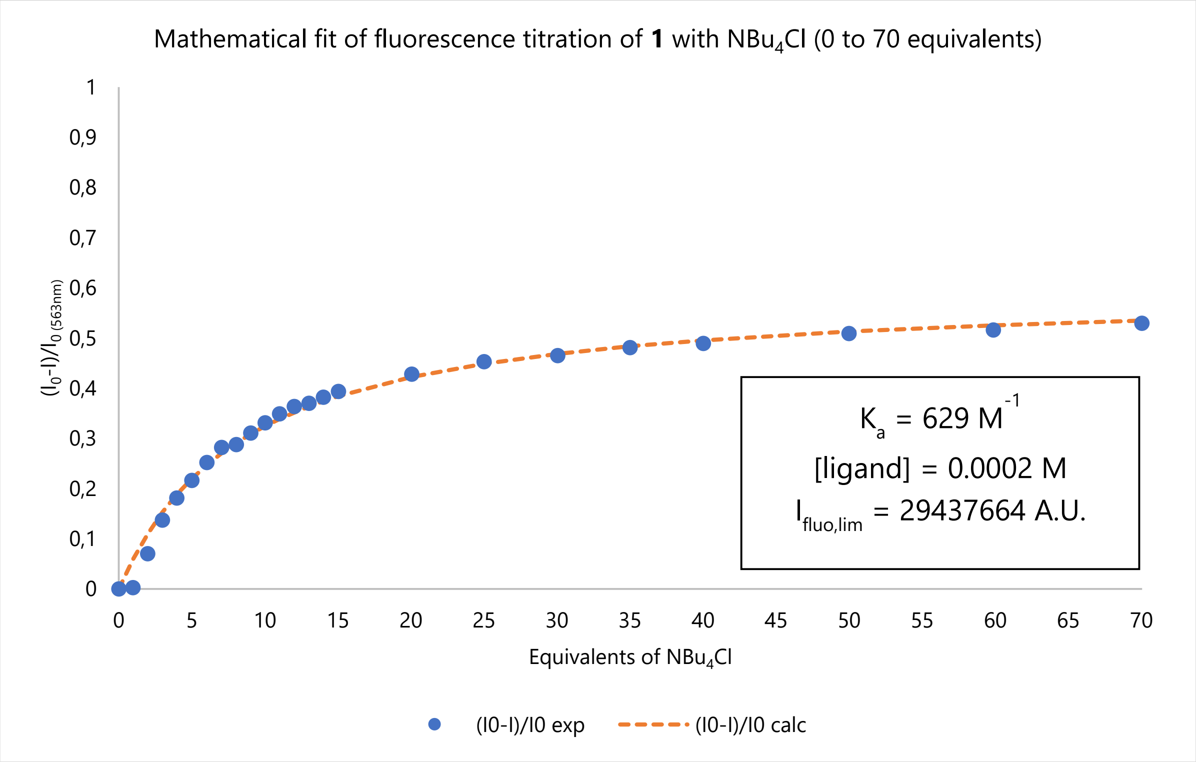

*Figure S67: Mathematical fit during the fluorescence titration of **1** with NBu_4Cl (0 to 70 equivalents) and determination of the association constant (here 3 refers to 1)*

```
[PROGRAM]
Name = SPECFIT
Version = 3.0

[FILE]
Name = RP123+TBACL_FLUO_FORMAT_SPECFIT.FAC
Path = C:\Users\Utilisateur\Desktop\
Date = 05-févr-21
Time = 12:18:34
Ncomp = 2
Nmeas = 24
Nwave = 281

[FACTOR ANALYSIS]
Tolerance = 1,000E-09
Max.Factors = 10
Num.Factors = 3
Significant = 2
Eigen Noise = 8,684E+04
Exp't Noise = 8,684E+04
#  Eigenvalue  Square Sum  Residual   Prediction
1  3,196E+18   1,887E+14   1,673E+05  Data Vector
2  1,378E+14   5,084E+13   8,684E+04  Data Vector
3  4,665E+12   4,618E+13   8,277E+04  Probably Noise

[MODEL]
Date = 05-févr-21
Time = 12:18:59
Model = 0
Index = 3
Function = 1
Species = 3
Params = 3

[SPECIES]         [COLORED]         [FIXED]           [SPECTRUM]
1 0 0             False             False
0 1 0             True              False
1 1 0             True              False

[SPECIES]         [FIXED]           [PARAMETER]            [ERROR]
1 0 0             True              0,00000E+00   +/-      0,00000E+00
0 1 0             True              0,00000E+00   +/-      0,00000E+00
1 1 0             False             2,88047E+00   +/-      1,91625E-02

[CONVERGENCE]
Iterations = 7
Convergence Limit = 1,000E-03
Convergence Found = 1,781E-06
Marquardt Parameter = 0,0
Sum(Y-y)^2 Residuals = 1,03482E+15
Std. Deviation of Fit(Y) = 3,91747E+05

[STATISTICS]
Experimental Noise = 8,684E+04
Relative Error Of Fit = 1,7996%
Durbin-Watson Factor = 1,5925
Goodness Of Fit, Chi^2 = 2,035E+01
Durbin-Watson Factor (raw data) = None
Goodness Of Fit, Chi^2 (raw data) = None

[COVARIANCE]
2,035E-03

[CORRELATION]
1,000E+00

[END FILE]
```

*Figure S68 : Determination of binding constant using SPECFIT software for the fluorescence titration of **1** with NBu_4Cl*

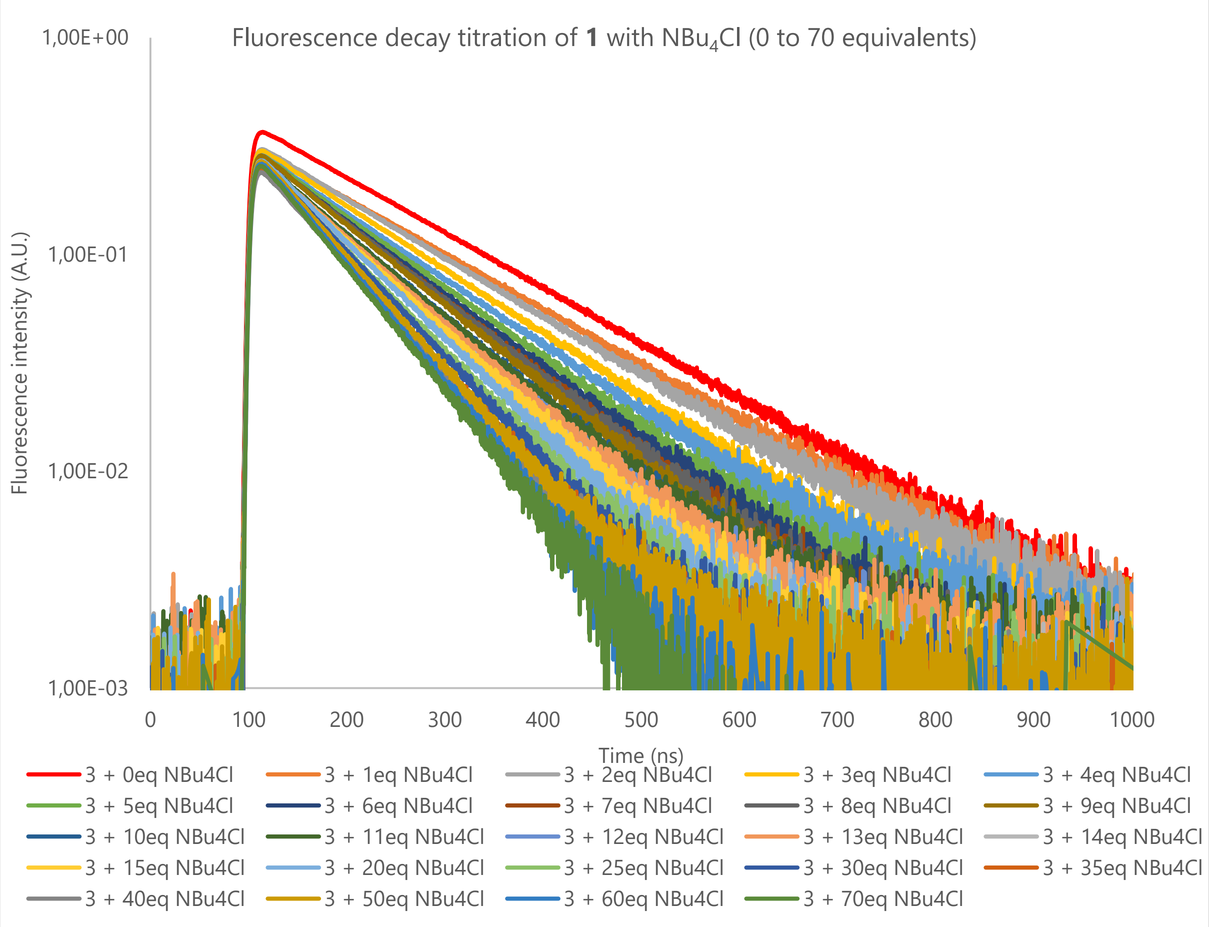

*Figure S69 : Fluorescence decay titration of **1** with NBu_4Cl (0 to 70 equivalents) – Logarithmic scale (here 3 refers to 1)*

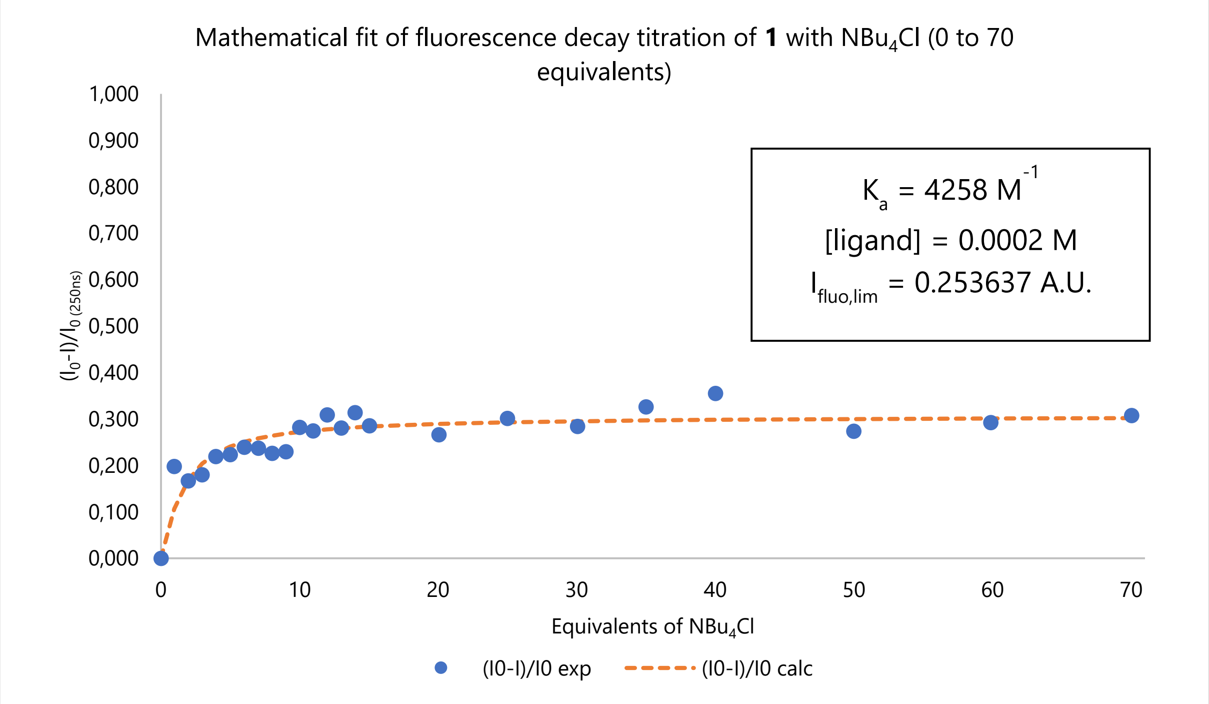

*Figure S70: Mathematical fit during the fluorescence decay titration of **1** with NBu_4Cl (0 to 70 equivalents) and determination of the association constant (here 3 refers to 1)*

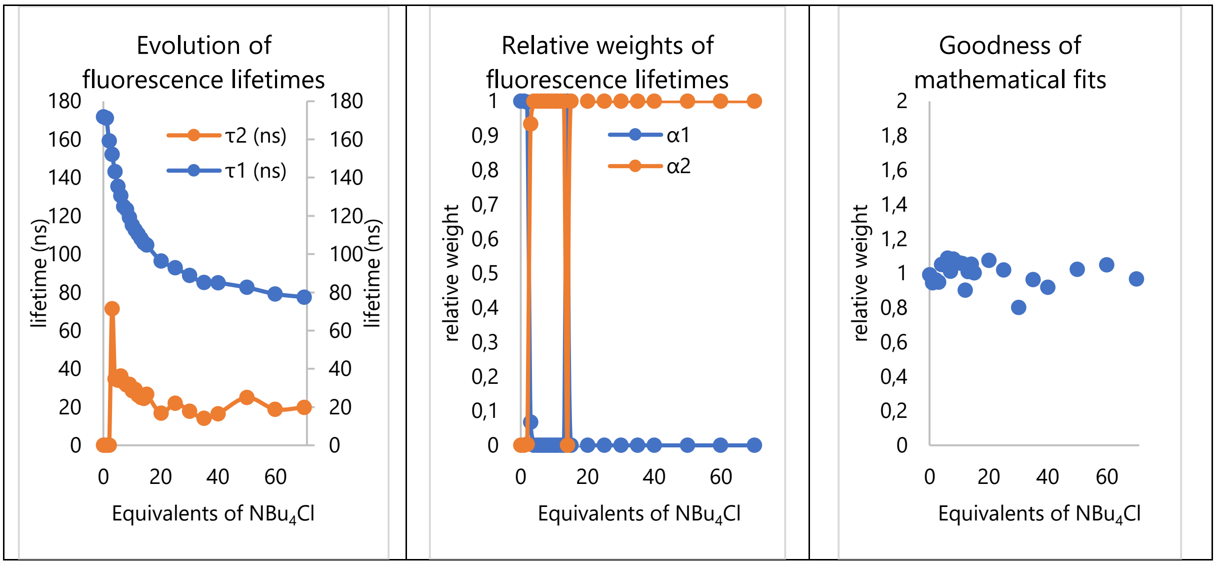

*Figure S71: Analysis of fluorescence decay titration of **1** with NBu_4Cl (0 to 70 equivalents)*

6.5 Titration of **2** with NBu_4Cl

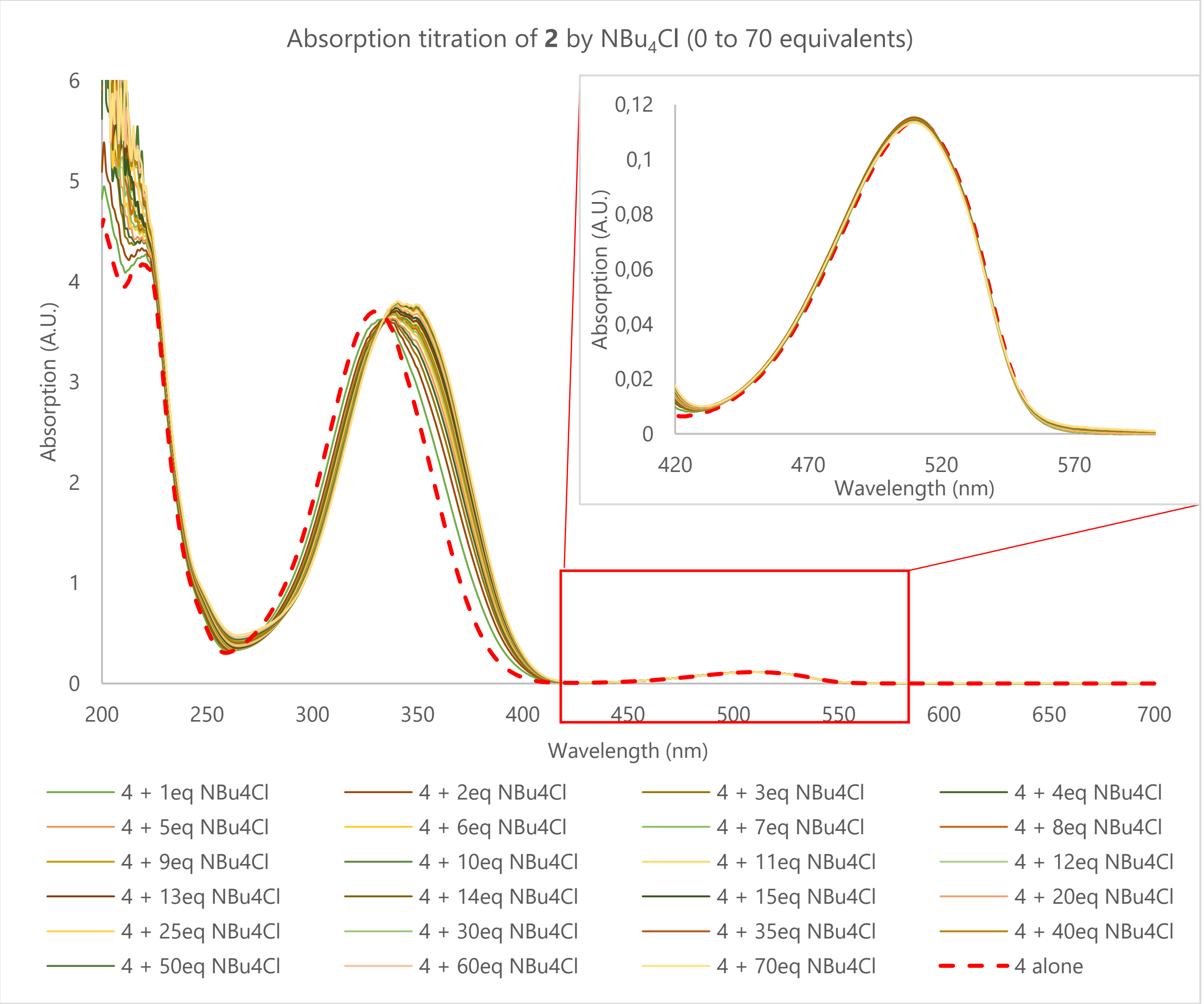

*Figure S72: Experimental UV-Visible spectra measured during the titration of **2** with NBu_4Cl (0 to 70 equivalents) (here 4 refers to 2)*

```
[PROGRAM]
Name = SPECFIT
Version = 3.0

[FILE]
Name = RP49+TBACL_ABS_FORMAT_SPECFIT.FAC
Path = C:\Users\Utilisateur\Desktop\
Date = 04-févr-21
Time = 14:23:35
Ncomp = 2
Nmeas = 24
Nwave = 501

[FACTOR ANALYSIS]
Tolerance = 1,000E-09
Max.Factors = 10
Num.Factors = 5
Significant = 1
Eigen Noise = 2,357E-01
Exp't Noise = 2,357E-01
#  Eigenvalue  Square Sum  Residual   Prediction
1  4,116E+04   6,678E+02   2,357E-01  Data Vector
2  2,773E+02   3,905E+02   1,802E-01  Possibly Data
3  1,271E+02   2,634E+02   1,480E-01  Probably Noise
4  8,959E+01   1,738E+02   1,202E-01  Probably Noise
5  5,463E+01   1,192E+02   9,958E-02  Probably Noise

[MODEL]
Date = 04-févr-21
Time = 14:23:55
Model = 0
Index = 3
Function = 1
Species = 3
Params = 3

[SPECIES]     [COLORED]     [FIXED]                [SPECTRUM]
1 0 0         False         False
0 1 0         True          False
1 1 0         True          False

[SPECIES]     [FIXED]       [PARAMETER]            [ERROR]
1 0 0         True          0,00000E+00   +/-      0,00000E+00
0 1 0         True          0,00000E+00   +/-      0,00000E+00
1 1 0         False         3,32400E+00   +/-      1,64108E-01

[CONVERGENCE]
Iterations = 9
Convergence Limit = 1,000E-03
Convergence Found = 2,278E-04
Marquardt Parameter = 0,0
Sum(Y-y)^2 Residuals = 3,62455E+02
Std. Deviation of Fit(Y) = 1,73628E-01

[STATISTICS]
Experimental Noise = 2,357E-01
Relative Error Of Fit = 9,3626%
Durbin-Watson Factor = 1,5883
Goodness Of Fit, Chi^2 = 5,428E-01
Durbin-Watson Factor (raw data) = None
Goodness Of Fit, Chi^2 (raw data) = None

[COVARIANCE]
2,108E-01

[CORRELATION]
1,000E+00

[END FILE]
```

Figure S73 : Determination of binding constant using SPECFIT software for the absorption titration of ***2*** *with NBu_4Cl*

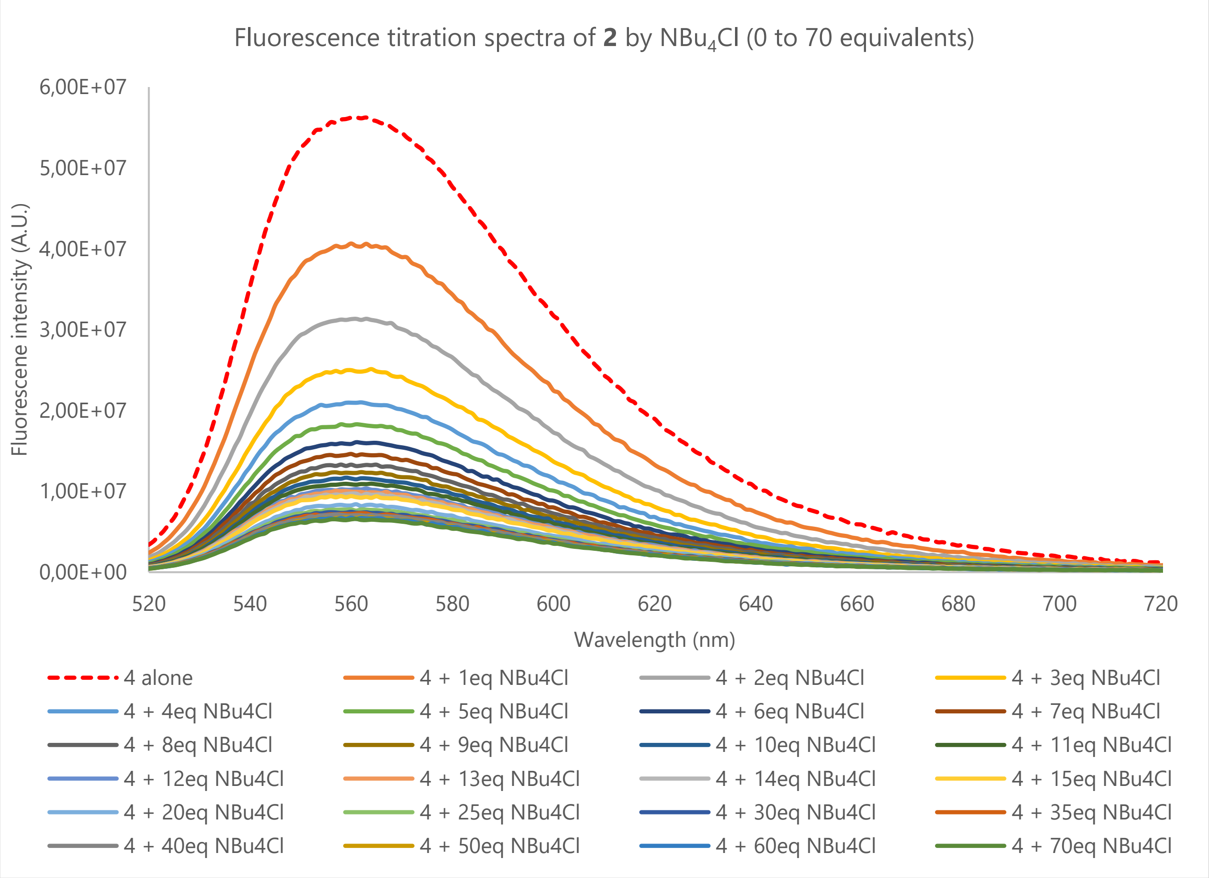

*Figure S74: Experimental fluorescence spectra measured during the titration of **2** with NBu_4Cl (0 to 70 equivalents) (here 4 refers to 2)*

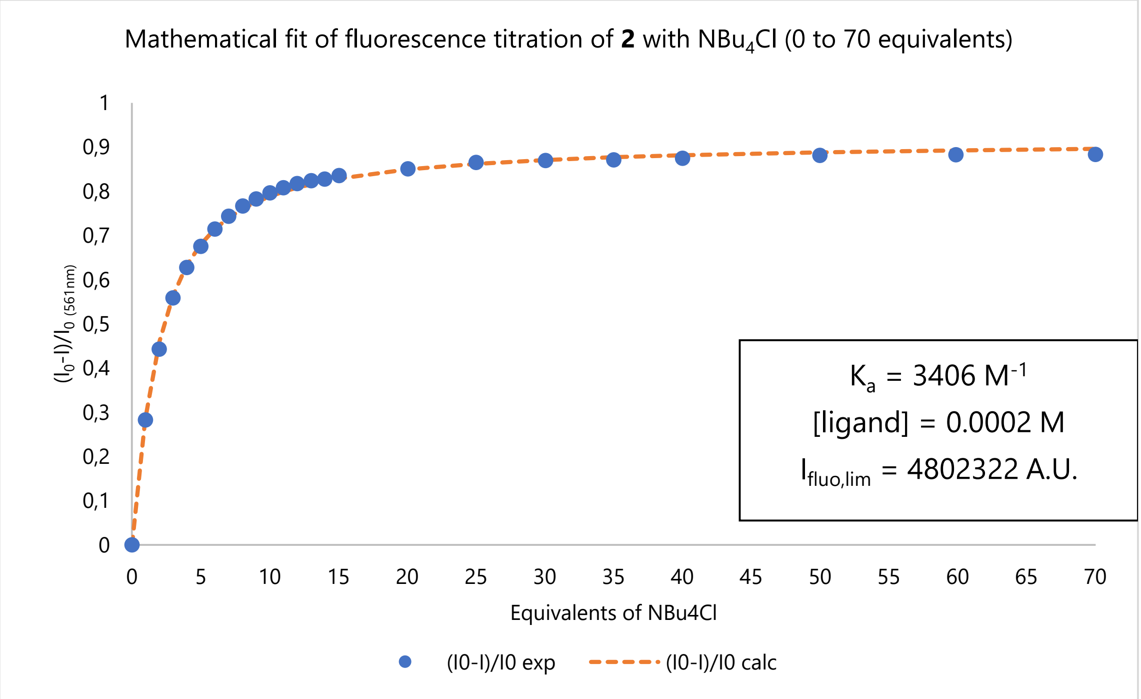

*Figure S75: Mathematical fit during the fluorescence titration of **2** with NBu_4Cl (0 to 70 equivalents) and determination of the association constant (here 4 refers to 2)*

```
[PROGRAM]
Name = SPECFIT
Version = 3.0

[FILE]
Name = RP49+TBACL_FLUO_FORMAT_SPECFIT.FAC
Path = C:\Users\Utilisateur\Desktop\
Date = 04-févr-21
Time = 14:37:00
Ncomp = 2
Nmeas = 24
Nwave = 281

[FACTOR ANALYSIS]
Tolerance = 1,000E-09
Max.Factors = 10
Num.Factors = 5
Significant = 2
Eigen Noise = 4,663E+04
Exp't Noise = 4,663E+04
#  Eigenvalue  Square Sum  Residual   Prediction
1  4,678E+17   5,502E+13   9,033E+04  Data Vector
2  4,036E+13   1,466E+13   4,663E+04  Data Vector
3  3,884E+12   1,078E+13   3,999E+04  Probably Noise
4  1,671E+12   9,107E+12   3,676E+04  Probably Noise
5  1,014E+12   8,093E+12   3,465E+04  Probably Noise

[MODEL]
Date = 04-févr-21
Time = 14:37:28
Model = 0
Index = 3
Function = 1
Species = 3
Params = 3

[SPECIES]      [COLORED]      [FIXED]             [SPECTRUM]
1 0 0          False          False
0 1 0          True           False
1 1 0          True           False

[SPECIES]      [FIXED]        [PARAMETER]         [ERROR]
1 0 0          True           0,00000E+00  +/-    0,00000E+00
0 1 0          True           0,00000E+00  +/-    0,00000E+00
1 1 0          False          3,54098E+00  +/-    7,48965E-03

[CONVERGENCE]
Iterations = 3
Convergence Limit = 1,000E-03
Convergence Found = 1,281E-07
Marquardt Parameter = 0,0
Sum(Y-y)^2 Residuals = 1,74638E+14
Std. Deviation of Fit(Y) = 1,60932E+05

[STATISTICS]
Experimental Noise = 4,663E+04
Relative Error Of Fit = 1,9323%
Durbin-Watson Factor = 0,3908
Goodness Of Fit, Chi^2 = 1,191E+01
Durbin-Watson Factor (raw data) = None
Goodness Of Fit, Chi^2 (raw data) = None

[COVARIANCE]
3,026E-04

[CORRELATION]
1,000E+00

[END FILE]
```

*Figure S76 : Determination of binding constant using SPECFIT software for the fluorescence titration of **2** with NBu_4Cl*

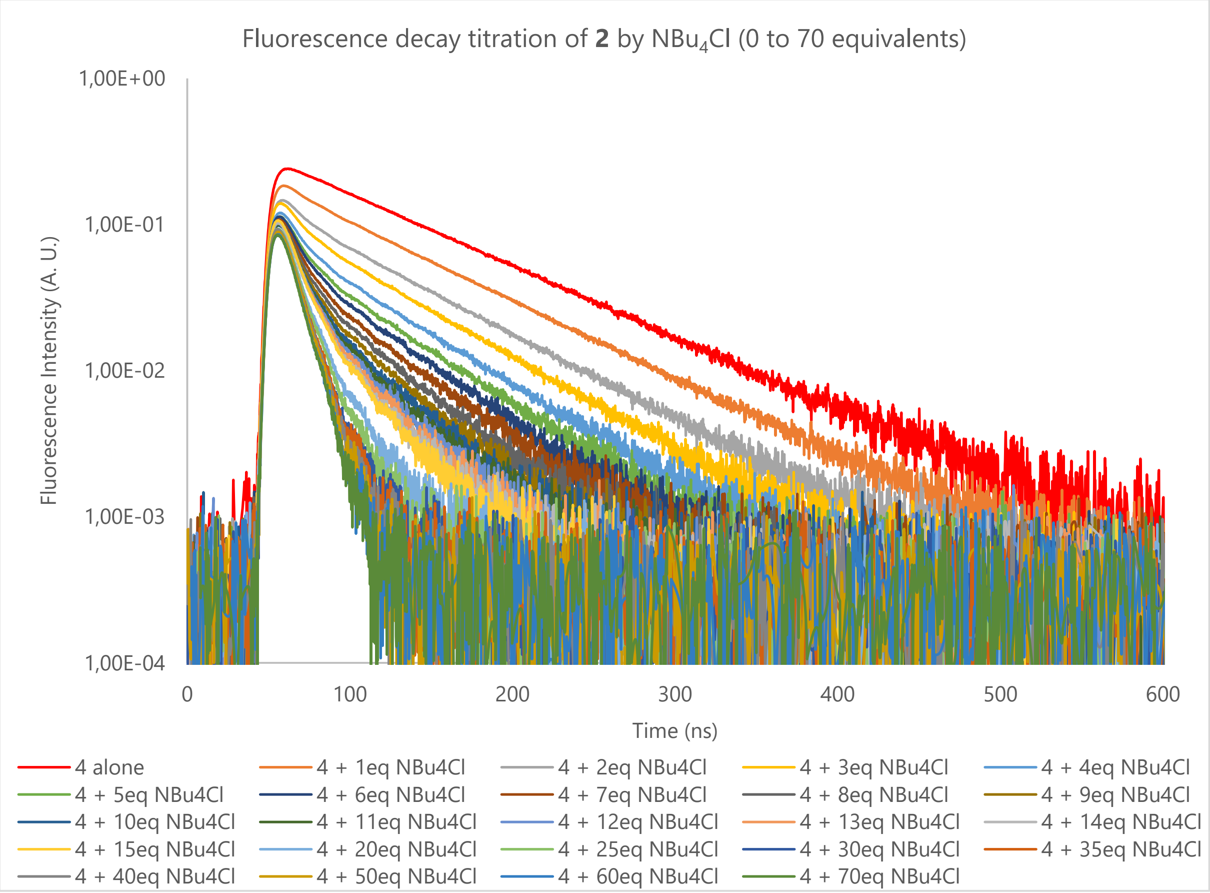

*Figure S77 : Fluorescence decay titration of **2** with NBu_4Cl (0 to 70 equivalents) – Logarithmic scale (here 4 refers to 2)*

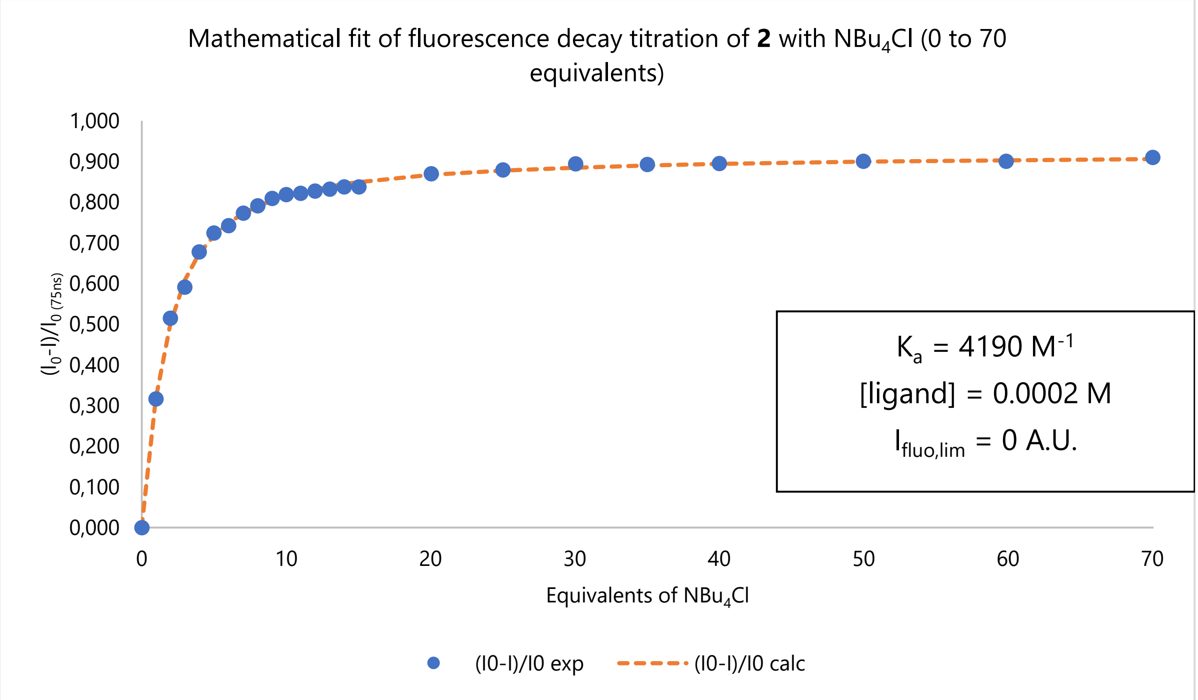

*Figure S78: Mathematical fit during the fluorescence decay titration of **2** with NBu_4Cl (0 to 70 equivalents) and determination of the association constant (here 4 refers to 2)*

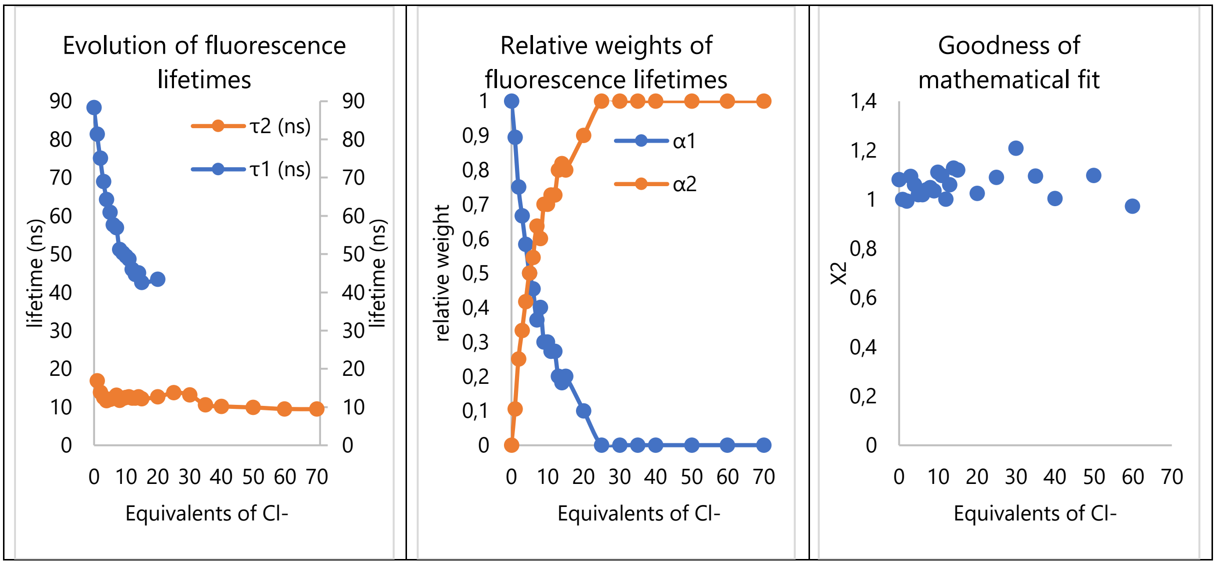

*Figure S79: Analysis of fluorescence decay titration of **2** with NBu_4Cl (0 to 70 equivalents)*

6.6 Titration of **3** with NBu_4Cl

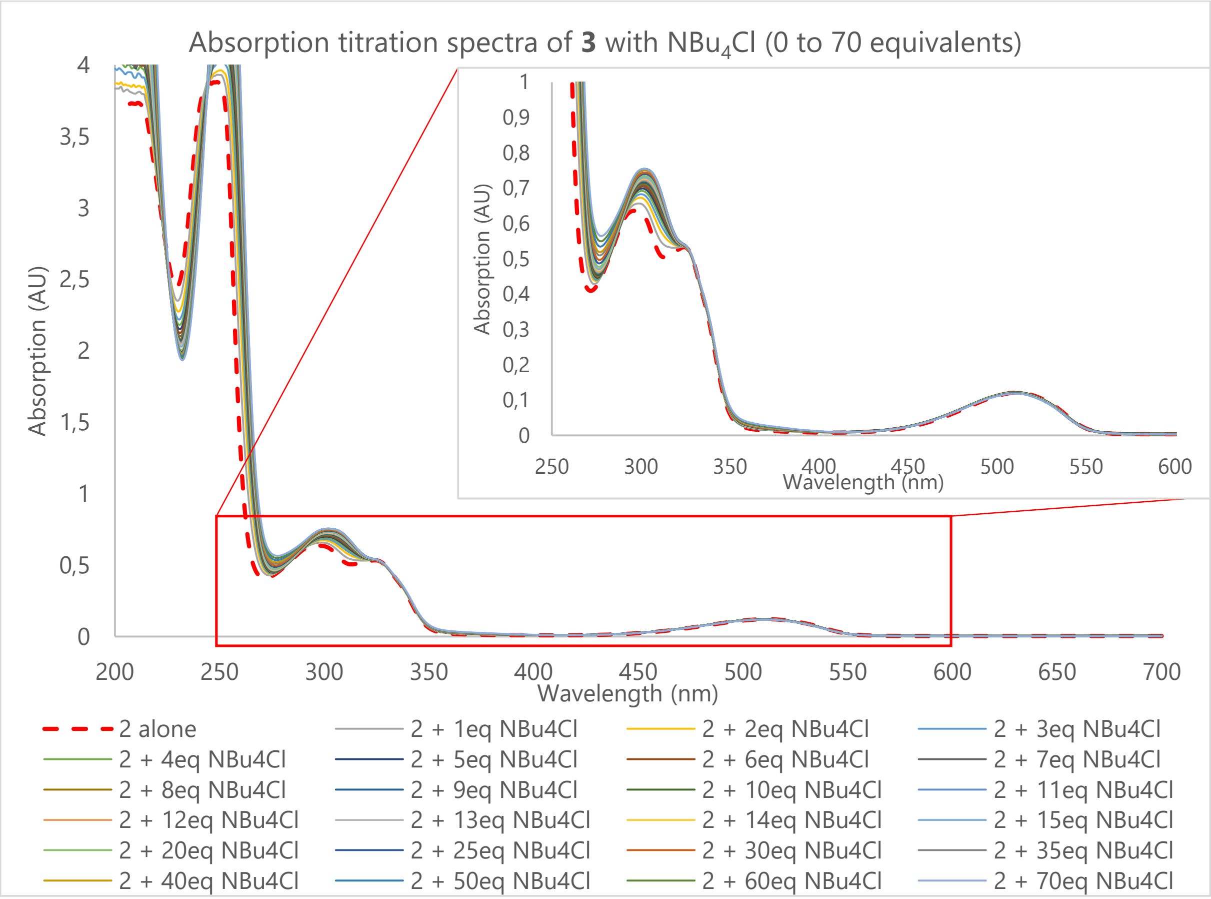

*Figure S80: Experimental UV-Visible spectra measured during the titration of **3** with NBu_4Cl (0 to 70 equivalents) (here 2 refers to 3)*

[PROGRAM]
Name = SPECFIT
Version = 3.0

[FILE]
Name = GG36+TBACL_ABS_FORMAT_SPECFIT.FAC
Path = C:\Users\Utilisateur\Desktop\
Date = 17-déc-20
Time = 13:12:56
Ncomp = 2
Nmeas = 24
Nwave = 501

[FACTOR ANALYSIS]
Tolerance = 1,000E-09
Max.Factors = 10
Num.Factors = 10
Significant = 7
Eigen Noise = 5,811E-03
Exp't Noise = 5,811E-03

#	Eigenvalue	Square Sum	Residual	Prediction
1	2,736E+04	1,076E+03	2,991E-01	Data Vector
2	9,257E+02	1,502E+02	1,118E-01	Data Vector
3	6,915E+01	8,104E+01	8,211E-02	Data Vector
4	4,789E+01	3,316E+01	5,252E-02	Data Vector
5	2,063E+01	1,253E+01	3,229E-02	Data Vector
6	1,154E+01	9,884E-01	9,069E-03	Data Vector
7	5,826E-01	4,057E-01	5,811E-03	Data Vector
8	2,475E-01	1,582E-01	3,629E-03	Possibly Data
9	8,580E-02	7,245E-02	2,456E-03	Probably Noise
10	2,627E-02	4,617E-02	1,960E-03	Probably Noise

[MODEL]
Date = 17-déc-20
Time = 13:13:53
Model = 0
Index = 3
Function = 1
Species = 3
Params = 3

[SPECIES]	[COLORED]	[FIXED]	[SPECTRUM]
1 0 0	False	False	
0 1 0	True	False	
1 1 0	True	False	

[SPECIES]	[FIXED]	[PARAMETER]		[ERROR]
1 0 0	True	0,00000E+00	+/-	0,00000E+00
0 1 0	True	0,00000E+00	+/-	0,00000E+00
1 1 0	False	1,33672E+00	+/-	3,16845E-01

[CONVERGENCE]
Iterations = 3
Convergence Limit = 1,000E-03
Convergence Found = 6,709E-05
Marquardt Parameter = 0,0
Sum(Y-y)^2 Residuals = 4,57003E+02
Std. Deviation of Fit(Y) = 1,94963E-01

[STATISTICS]
Experimental Noise = 5,811E-03
Relative Error Of Fit = 12,7812%
Durbin-Watson Factor = 0,7824
Goodness Of Fit, Chi^2 = 1,126E+03
Durbin-Watson Factor (raw data) = 0,7826
Goodness Of Fit, Chi^2 (raw data) = None

[COVARIANCE]
1,154E+00

[CORRELATION]
1,000E+00

[END FILE]

*Figure S81: Determination of binding constant using SPECFIT software for the UV-Visible titration of **3** with NBu_4Cl*

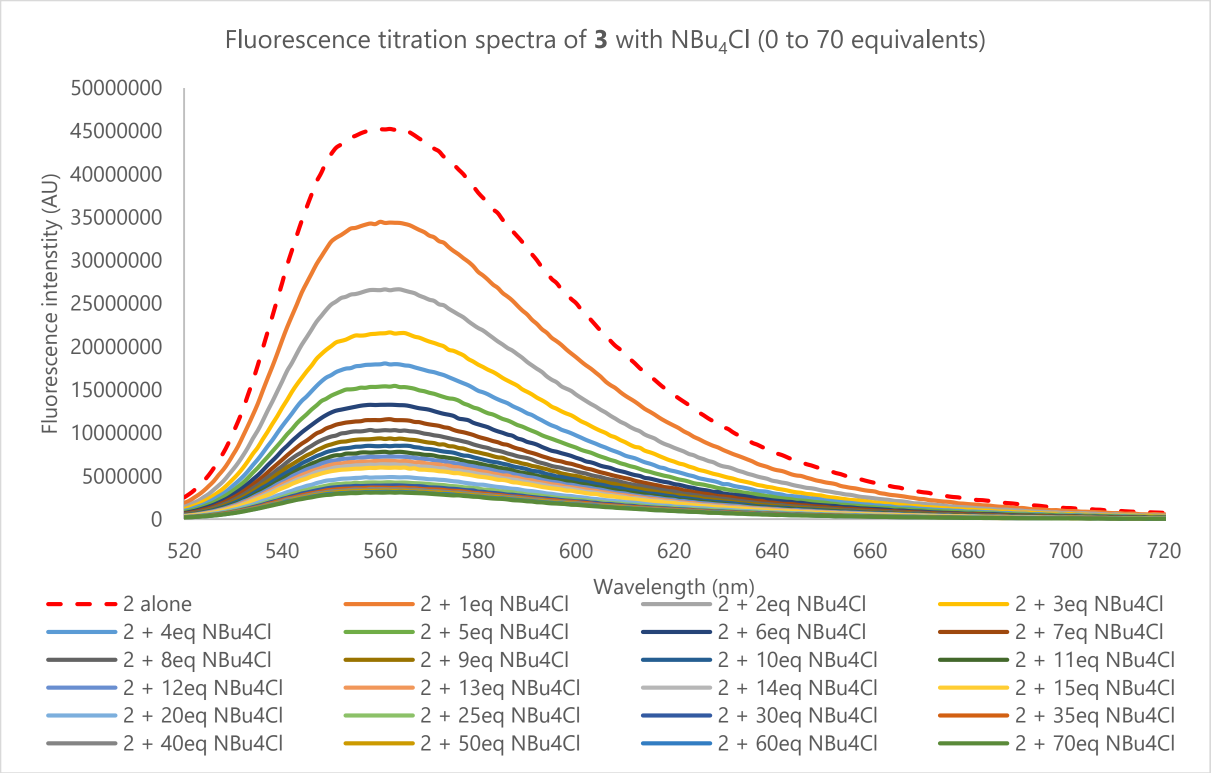

*Figure S82: Experimental fluorescence spectra measured during the titration of **3** with NBu_4Cl (0 to 70 equivalents) (here 2 refers to 3)*

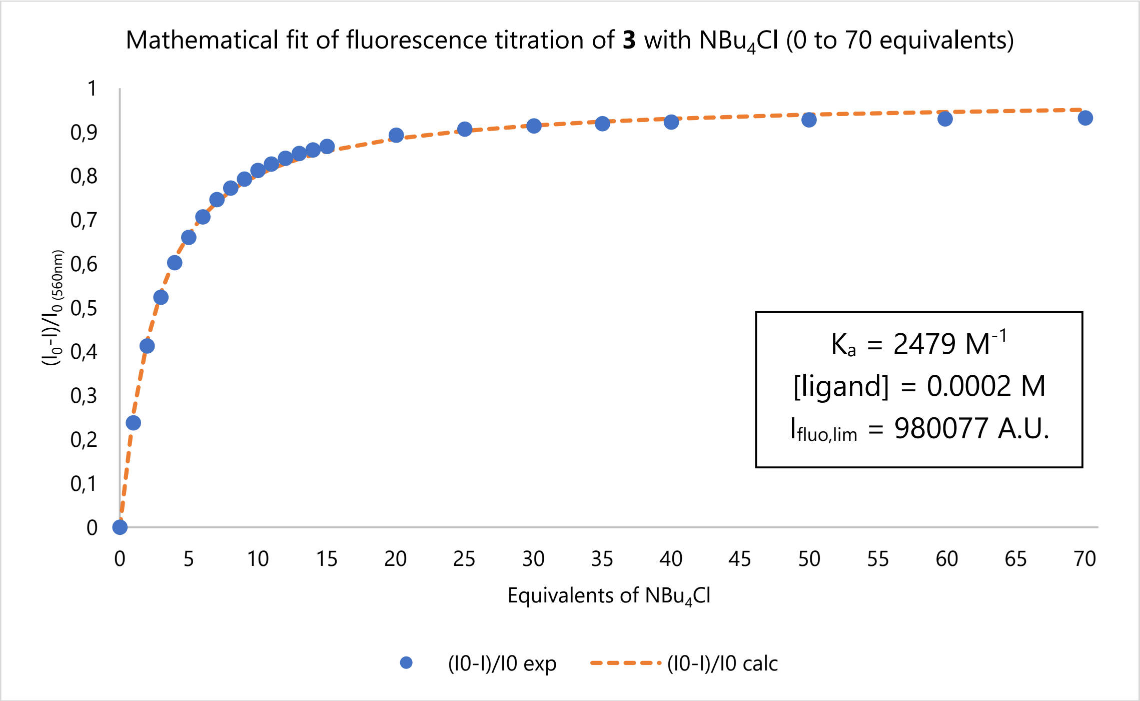

*Figure S83: Mathematical fit during the fluorescence titration of **3** with NBu_4Cl (0 to 70 equivalents) and determination of the association constant*

```
[PROGRAM]
Name = SPECFIT
Version = 3.0

[FILE]
Name = GG36+TBACL_FLUO_FORMAT_SPECFIT.FAC
Path = C:\Users\Utilisateur\Desktop\
Date = 17-déc-20
Time = 13:20:50
Ncomp = 2
Nmeas = 24
Nwave = 281

[FACTOR ANALYSIS]
Tolerance = 1,000E-09
Max.Factors = 10
Num.Factors = 5
Significant = 2
Eigen Noise = 1,758E+04
Exp't Noise = 1,758E+04
#  Eigenvalue  Square Sum  Residual   Prediction
1  2,976E+17   6,357E+12   3,070E+04  Data Vector
2  4,273E+12   2,084E+12   1,758E+04  Data Vector
3  3,610E+11   1,723E+12   1,599E+04  Probably Noise
4  2,509E+11   1,472E+12   1,478E+04  Probably Noise
5  1,981E+11   1,274E+12   1,375E+04  Probably Noise

[MODEL]
Date = 17-déc-20
Time = 13:26:49
Model = 0
Index = 3
Function = 1
Species = 3
Params = 3

[SPECIES]      [COLORED]      [FIXED]        [SPECTRUM]
1 0 0          False          False
0 1 0          True           False
1 1 0          True           False

[SPECIES]      [FIXED]        [PARAMETER]         [ERROR]
1 0 0          True           0,00000E+00   +/-   0,00000E+00
0 1 0          True           0,00000E+00   +/-   0,00000E+00
1 1 0          False          3,41071E+00   +/-   9,48765E-03

[CONVERGENCE]
Iterations = 4
Convergence Limit = 1,000E-03
Convergence Found = 1,128E-04
Marquardt Parameter = 0,0
Sum(Y-y)^2 Residuals = 2,22323E+14
Std. Deviation of Fit(Y) = 1,81579E+05

[STATISTICS]
Experimental Noise = 1,758E+04
Relative Error Of Fit = 2,7341%
Durbin-Watson Factor = 0,2801
Goodness Of Fit, Chi^2 = 1,067E+02
Durbin-Watson Factor (raw data) = None
Goodness Of Fit, Chi^2 (raw data) = None

[COVARIANCE]
4,878E-04

[CORRELATION]
1,000E+00

[END FILE]
```

*Figure S84 : Determination of binding constant using SPECFIT software for the fluorescence titration of **3** with NBu_4Cl*

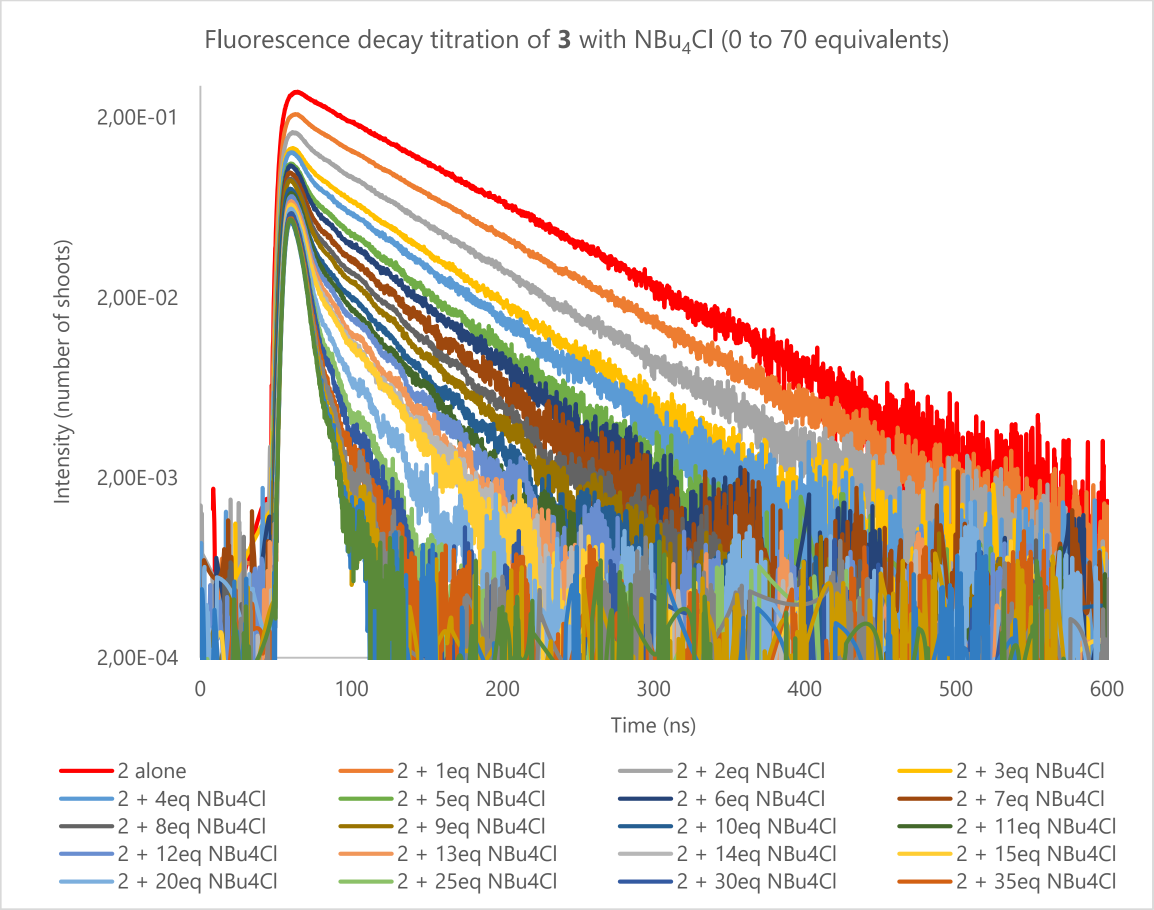

*Figure S85 : Fluorescence decay titration of **3** with NBu_4Cl (0 to 70 equivalents) – Logarithmic scale (here 2 refers to 3)*

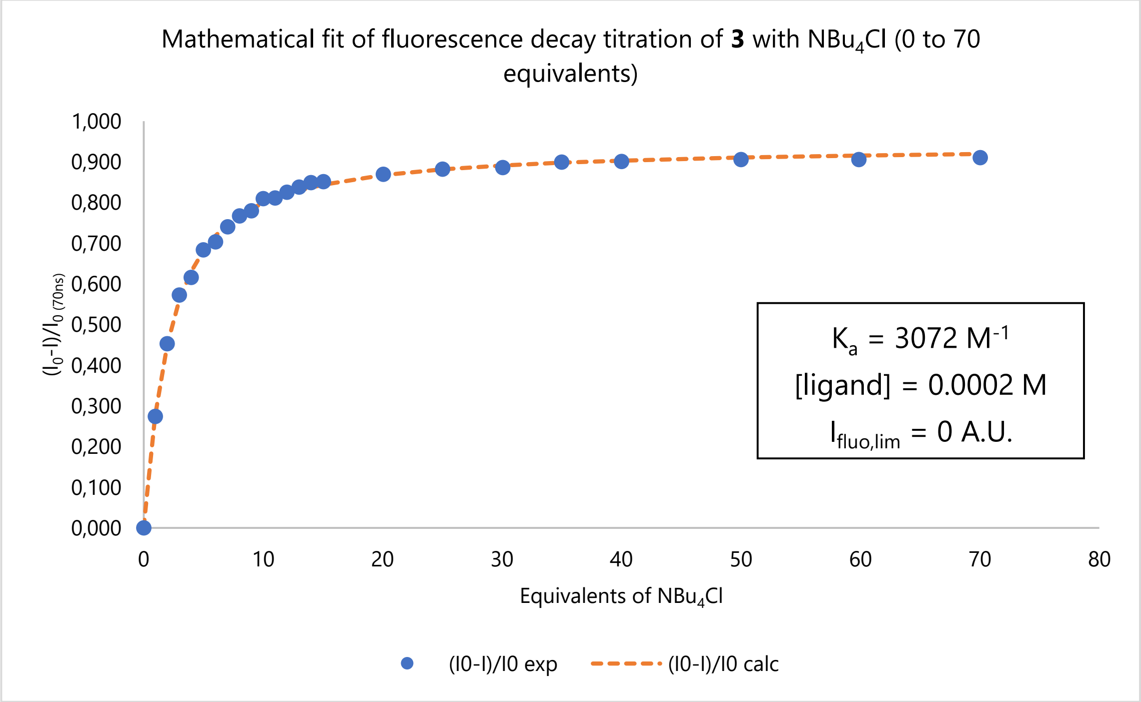

*Figure S86: Mathematical fit during the fluorescence decay titration of **3** with NBu_4Cl (0 to 70 equivalents) and determination of the association constant (here 2 refers to 3)*

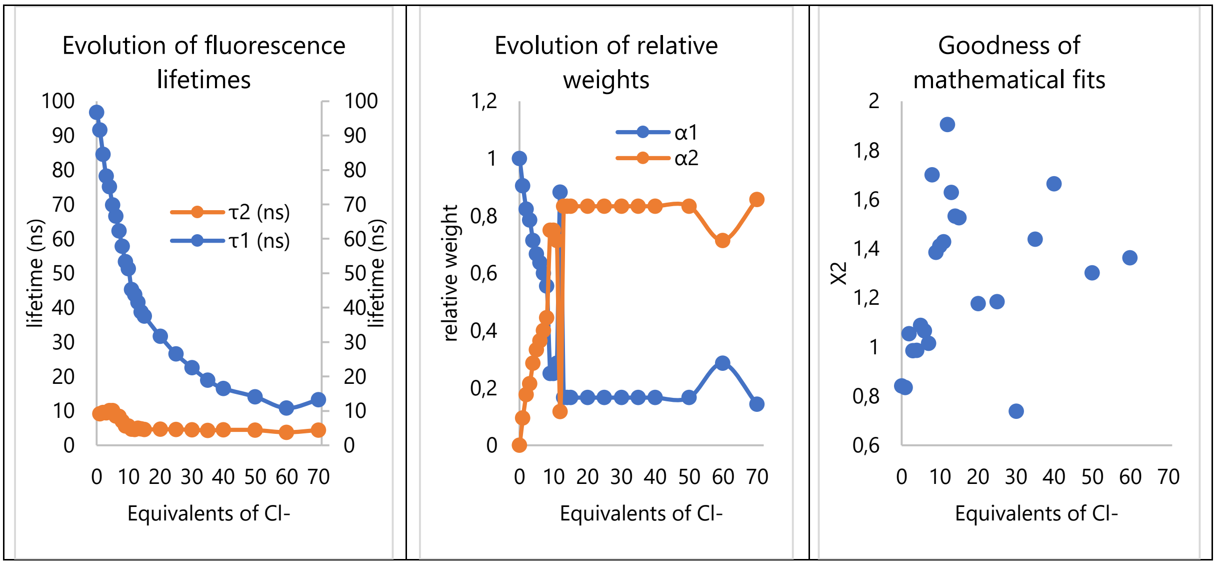

*Figure S87: Analysis of fluorescence decay titration of **3** with NBu_4Cl (0 to 70 equivalents)*

6.7 Stern-Volmer analysis

The Stern-Volmer equation:

$$\frac{I_0}{I} = 1 + K_{SV}[Q] \qquad \text{eq 1}$$

applies when the complex is not fluorescent. However in our case it presents a residual fluorescence so the equation must be modified to account for it and determine the fluorescence intensity of the ligand at each point.

The fluorescence intensity can be decomposed in:

$$I = I_L + I_C \qquad \text{eq 2}$$

where I_L is the free ligand fluorescence intensity et I_C is the fluorescence of the complex. I_C can be expressed as:

$$I_c = x_C I_{res} \qquad \text{eq 3}$$

where x_C is the molar fraction of the complex relative to the total ligand concentration and I_{res} the residual fluorescence at the final point of the titration. The ligand fluorescence intensity can thus be expressed as

$$I_L = I - x_C I_{res} \qquad \text{eq 4}$$

The molar fraction of the complex x_C can be calculated from the expression of the association constant K:

$$K = \frac{[C]}{[L]\times[M]} \qquad \text{eq 5}$$

Using mass conservation laws the concentration of complex can be written as:

$$K[C]^2 - (K[M]_0 + K[L]_0 + 1)[C] + K[M]_0[L]_0 = 0 \quad \text{eq 6}$$

where $[M]_0$ and $[L]_0$ are the total concentration of salt and ligand respectively. The solution of this equation is:

$$[C] = \frac{(K[M]_0+K[L]_0+1)-\sqrt{\Delta}}{2K} \qquad \text{eq 7}$$

where Δ is:

$$\Delta = (K[M]_0 + K[L]_0 + 1)^2 - 4K^2[M]_0[L]_0 \qquad \text{eq 8}$$

The value of K was taken equal to the one obtain by the SPECFIT analysis of the fluorescence spectra ($K_{A,\text{ fluo SPECFIT}}$ in table XXX)

The plot $\frac{I_0}{I_L}$ as a function of the salt concentration are given in Figure S88

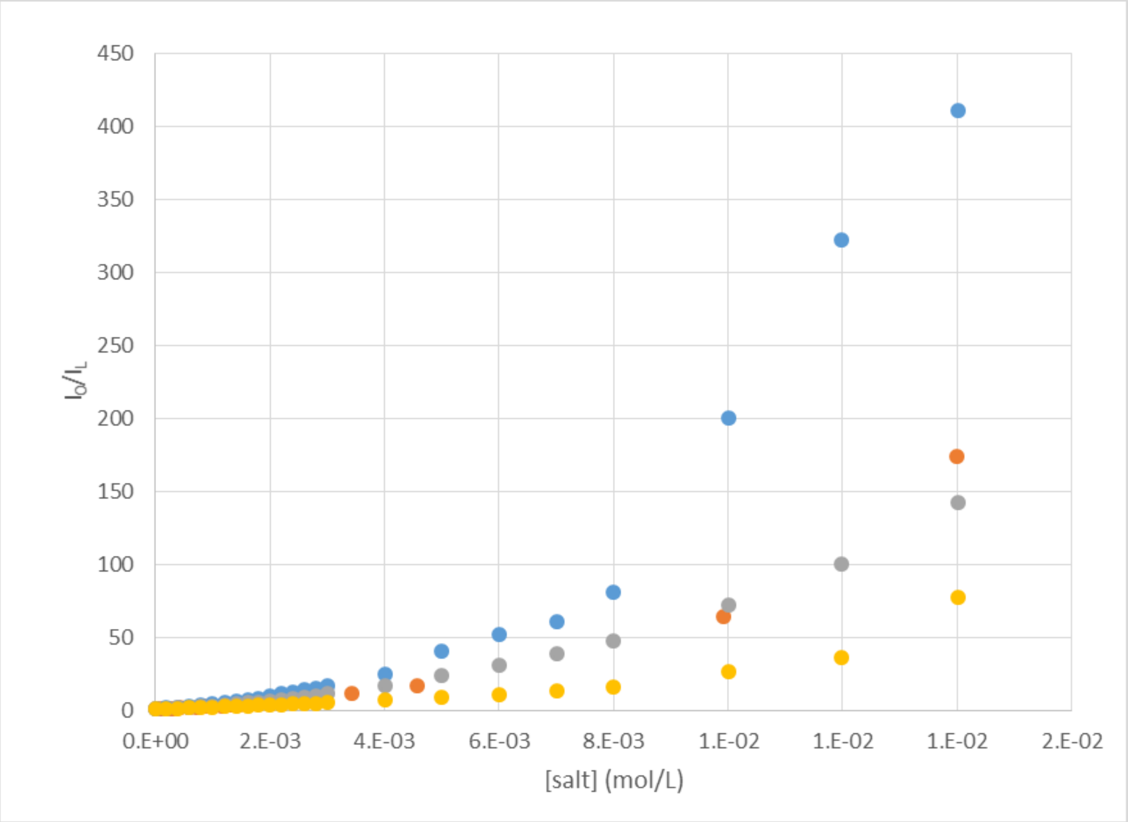

*Figure S88: Modified Stern-Volmer plot for compounds **1** (grey), **2** (blue), **3** (yellow) and **7** (orange).*

7. Bibliography

[1] Y. H. Gong, F. Miomandre, R. Méallet-Renault, S. Badré, L. Galmiche, J. Tang, P. Audebert, G. Clavier, *European J. Org. Chem.* **2009**, *2009*, 6121–6128.

[2] H. E. Gottlieb, V. Kotlyar, A. Nudelman, *J. Org. Chem.* **1997**, *62*, 7512–7515.

[3] D. Jacquemin, E. A. Perpete, I. Ciofini, C. Adamo, *Acc. Chem. Res.* **2009**, *42*, 326–334.

[4] R. Plais, G. Gouarin, A. Gaucher, V. Haldys, A. Brosseau, G. Clavier, J.-Y. Salpin, D. Prim,

ChemPhysChem **2020**, *21*, 1249–1257.

[5] E. R. Johnson, S. Keinan, P. Mori-Sánchez, J. Contreras-García, A. J. Cohen, W. Yang, *J. Am. Chem. Soc.* **2010**, *132*, 6498–6506.

[6] T. Lu, F. Chen, *J. Comput. Chem.* **2012**, *33*, 580–592.

[7] W. Humphrey, A. Dalke, K. Schulten, *J. Mol. Graph.* **1996**, *14*, 33–38.

[8] G. González-Gaitano, G. Tardajos, *J. Chem. Educ.* **2004**, *81*, 270–274.

[9] B. Valeur, *Molecular Fluorescence: Principles and Applications, First Edition*, Wiley-VCH, **2001**.

[10] H. Gampp, M. Maeder, C. J. Meyer, A. D. Zuberbühler, *Talanta* **1985**, *32*, 95–101.

[11] H. Gampp, M. Maeder, C. J. Meyer, A. D. Zuberbühler, *Talanta* **1985**, *32*, 257–264.

[12] C. Würth, M. Grabolle, J. Pauli, M. Spieles, U. Resch-Genger, *Nat. Protoc.* **2013**, *8*, 1535–1550.
